# A Transcriptional Mechanism Integrating Inputs from Extracellular Signals to Activate Hippocampal Stem Cells

**DOI:** 10.1016/j.neuron.2014.08.004

**Published:** 2014-09-03

**Authors:** Jimena Andersen, Noelia Urbán, Angeliki Achimastou, Ayako Ito, Milesa Simic, Kristy Ullom, Ben Martynoga, Mélanie Lebel, Christian Göritz, Jonas Frisén, Masato Nakafuku, François Guillemot

**Affiliations:** 1 Division of Molecular Neurobiology, MRC National Institute for Medical Research, Mill Hill, London NW7 1AA, UK; 2 Division of Developmental Biology, Cincinnati Children’s Hospital Research Foundation, Cincinnati, OH 45229-3039, USA; 3 Department for Cell and Molecular Biology, Karolinska Institute, 17177 Stockholm, Sweden

## Abstract

The activity of adult stem cells is regulated by signals emanating from the surrounding tissue. Many niche signals have been identified, but it is unclear how they influence the choice of stem cells to remain quiescent or divide. Here we show that when stem cells of the adult hippocampus receive activating signals, they first induce the expression of the transcription factor Ascl1 and only subsequently exit quiescence. Moreover, lowering *Ascl1* expression reduces the proliferation rate of hippocampal stem cells, and inactivating *Ascl1* blocks quiescence exit completely, rendering them unresponsive to activating stimuli. *Ascl1* promotes the proliferation of hippocampal stem cells by directly regulating the expression of cell-cycle regulatory genes. *Ascl1* is similarly required for stem cell activation in the adult subventricular zone. Our results support a model whereby *Ascl1* integrates inputs from both stimulatory and inhibitory signals and converts them into a transcriptional program activating adult neural stem cells.

## Highlights

• Ascl1 is expressed specifically by activated stem cells of the hippocampus
• Activating signals induce first Ascl1 expression and subsequently quiescence exit
• Hippocampal stem cells expressing low levels of Ascl1 have reduced activity
• Stem cells lacking *Ascl1* remain permanently quiescent and unresponsive to stimuli

## Teaser

Multiple extracellular signals regulate the activity of stem cells in the adult hippocampus. Andersen et al. show here that induction of the proneural protein Ascl1 in response to activation signals is absolutely required for stem cells to exit quiescence.

## Introduction

Adult stem cells maintain tissue function and integrity throughout the lifetime of an organism. They produce mature progenies to replace short-lived cells and repair tissue damage while maintaining their numbers through self-renewing divisions (Simons and Clevers, 2011). Many tissue stem cells are relatively quiescent, which delays their attrition and minimizes the accumulation of deleterious mutations (Orford and Scadden, 2008). The transit of stem cells between quiescent and activated states is not well understood in most systems. Elucidating the mechanisms that control the activation of tissue stem cells is an important goal in stem cell biology.

A variety of extracellular signals present in stem cell niches have been shown to influence the activity of tissue stem cells (Fuchs et al., 2004, Goldstein and Horsley, 2012, Kuang et al., 2008). For example, BMP signaling induces quiescence, while Wnts promote proliferation of skin and blood stem cells (Blank et al., 2008, Fuchs et al., 2004). However, the cell-intrinsic mechanisms that mediate the activity of extrinsic signals and promote stem cell quiescence or proliferation are poorly characterized. Niche signals might act by inducing the expression or activity of transcription factors that in turn regulate the large number of genes differentially expressed between quiescent and active stem cells (Lien et al., 2011, Martynoga et al., 2013, Venezia et al., 2004). Transcription factors have indeed been shown to regulate stem cell activity in various tissues by controlling their proliferation, survival, or differentiation (Akala and Clarke, 2006, Goldstein and Horsley, 2012). However, it is not known in most instances how these factors are regulated (Niu et al., 2011, Osorio et al., 2008).

In the adult mammalian nervous system, neural stem cells (NSCs) are found mostly in two regions of the anterior brain, the dentate gyrus (DG) of the hippocampus and the ventricular-subventricular zone (V-SVZ) lining the lateral ventricles, where stem cells produce new neurons that integrate into neuronal circuits of the hippocampus and olfactory bulb, respectively (Fuentealba et al., 2012, Ming and Song, 2011). Most adult NSCs are quiescent and rest in G_0_, with only a small fraction progressing through the cell cycle at any time. NSC divisions result in the generation of transit-amplifying cells or intermediate progenitor cells (IPCs) that undergo a limited number of rapid divisions before they exit the cell cycle and differentiate into neurons (Ming and Song, 2011, Ponti et al., 2013). Clonal analysis in the adult mouse hippocampus in vivo has provided evidence that hippocampal NSCs, also called radial glia-like cells (RGLs), are multipotent and can generate both neurons and astrocytes, and that they use two modes of divisions to self-renew. Some RGLs divide asymmetrically to generate a new RGL and an IPC or an astrocyte, while others divide symmetrically into two new RGLs (Bonaguidi et al., 2011).

A particularly important feature of hippocampal neurogenesis is its regulation by a variety of physiological stimuli (Ming and Song, 2011). Neurogenesis in the hippocampus declines sharply with age, due in part to a reduction of the fraction of RGLs that divide, and it is suppressed by stress and depression (Lee et al., 2011, Ming and Song, 2011). Conversely, an enriched environment, task learning, or seizures stimulate hippocampal neurogenesis, in part by stimulating RGL divisions (Kronenberg et al., 2003, Ming and Song, 2011). Some of the extracellular signals that regulate RGL activity have been identified (Ming and Song, 2011). In particular, the BMP and Notch signaling pathways maintain RGLs in a quiescent state (Ables et al., 2010, Ehm et al., 2010, Mira et al., 2010), while the Wnt and IGF-1 pathways, among others, promote RGL divisions and stimulate neurogenesis (Bracko et al., 2012, Jang et al., 2013, Lie et al., 2005, Qu et al., 2010). Little is known, however, of how the activity of physiological stimuli or extrinsic signals is transduced inside RGLs to control their divisions. The orphan nuclear receptor Tlx is required to maintain RGLs in proliferation (Niu et al., 2011, Qu et al., 2010, Zhang et al., 2008), but how Tlx expression and activity are regulated has not been addressed.

The proneural transcription factor achaete-scute homolog 1 (Ascl1/Mash1) is an important regulator of neurogenesis in the embryonic nervous system. It is expressed by dividing progenitors and promotes their proliferation, specification, and differentiation into neurons (Bertrand et al., 2002, Castro et al., 2011). Moreover, ectopic expression of *Ascl1* can reprogram various cell types into neurons (Berninger et al., 2007, Yang et al., 2011). Ascl1 is also expressed in the DG and V-SVZ of the adult rodent brain, but its function there has not been examined. Ascl1 adult expression is mostly confined to IPCs (Lugert et al., 2012, Parras et al., 2004, Pastrana et al., 2009), but recent genetic lineage-tracing experiments have established that it is also present in self-renewing stem cells in both the V-SVZ and hippocampus (Kim et al., 2011). Consistent with this finding, Ascl1 was found expressed by a small subset of cycling stem cells in both neurogenic zones (Breunig et al., 2007, Kim et al., 2011). Here we show that Ascl1 expression is rapidly induced by neurogenic signals in hippocampal RGLs, and that *Ascl1* has a crucial role in RGL activation in both DG and V-SVZ. Ascl1 is specifically expressed in activated adult stem cells and is specifically required for the exit of stem cells from quiescence.

## Results

### Ascl1 Is Expressed by Activated Stem Cells in the Adult Hippocampus

**Figure 1:**
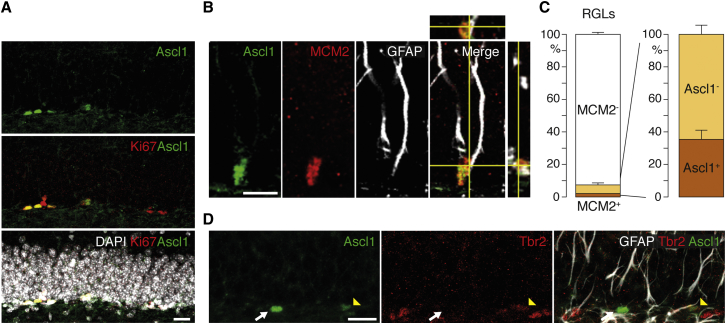
Expression of Ascl1 by Proliferating Stem and Progenitor Cells in the Adult Hippocampus (A) Colocalization of Ascl1 protein (green) and proliferation marker Ki67 (red) shows that Ascl1 is expressed in proliferating cells in the dentate gyrus (DG) of a P60 mouse. Counterstained by DAPI (white). (B) Ascl1 (green), MCM2 (a marker of cell cycle and G_0_ to early G_1_ transition, red), and neural stem cell marker GFAP (white) are colocalized in an activated radial glia-like stem cell (RGL). The z stack of the merged confocal picture is shown along the x axis (top) and the y axis (right). (C) The percentage of activated (MCM2^+^) and Ascl1-expressing (Ascl1^+^) RGLs in the DG of P60 mice show that Ascl1 is expressed in a third of activated RGLs. n = 3. (D) Labeling for Ascl1 (green), the intermediate progenitor cell (IPC) marker Tbr2 (red), and GFAP (white) in a P60 DG show that Ascl1 is expressed in both Tbr2^+^ (yellow arrowhead) and Tbr2^−^ (white arrow) GFAP^−^ IPCs. Scale bars, 20 μm in (A) and (D) and 10 μm in (B). Values represent mean values, and error bars represent SDs.

To study the function of *Ascl1* in hippocampal neurogenesis, we first characterized its expression in the adult hippocampus. Labeling of 2-month-old mouse brains with a monoclonal antibody against Ascl1 showed that in the hippocampus, Ascl1-expressing cells are restricted to the subgranular zone (SGZ) of the DG (Figure 1A). Double labeling for Ascl1 and the cell proliferation marker Ki67 showed that most Ascl1-expressing cells are proliferating (Figure 1A). Double labeling for Ascl1 and markers of progenitor cells of the DG neurogenic lineage showed that Ascl1 is expressed by three distinct progenitor cell populations. It is expressed by a small subset of RGLs, identified by their radial morphology and expression of GFAP. Most Ascl1-positive RGLs are activated, as 83.3% ± 16.7% of them express the cell cycle and cell activation marker MCM2 (Machida et al., 2005, Niu et al., 2011) (Figures 1B and 1C). Ascl1-positive RGLs represent 35.6% ± 3.2% of activated (MCM2^+^) RGLs and 2.0% ± 0.7% of all RGLs (Figure 1C). Ascl1 is also expressed by nonradial GFAP^+^ cells in the SGZ, which are also considered to be hippocampal NSCs (Lugert et al., 2010, Suh et al., 2007), and by IPCs, characterized by their SGZ location, proliferative state, and lack of GFAP expression (Figure 1D). Ascl1-positive IPCs represent 13.5% ± 4.1% of all IPCs and are subdivided into two subsets that differ in the expression of the IPC marker Tbr2 (Kempermann et al., 2004, Ming and Song, 2011). Ascl1 is not expressed by more mature cells in the lineage, including doublecortin (DCX)^+^ neuroblasts and NeuN^+^ granule neurons (Figure S1; data not shown). Together, these data agree with previous reports showing that Ascl1 expression is restricted to the earliest stages of the neurogenic lineage of the adult DG, including proliferating RGLs (Breunig et al., 2007, Kim et al., 2011) and early IPCs (Lugert et al., 2010, Lugert et al., 2012), and that it is downregulated before IPCs begin to express neuronal markers and exit the cell cycle.

### Ascl1 Expression in Hippocampal Stem Cells Is Induced by Neurogenic Stimuli

**Figure 2:**
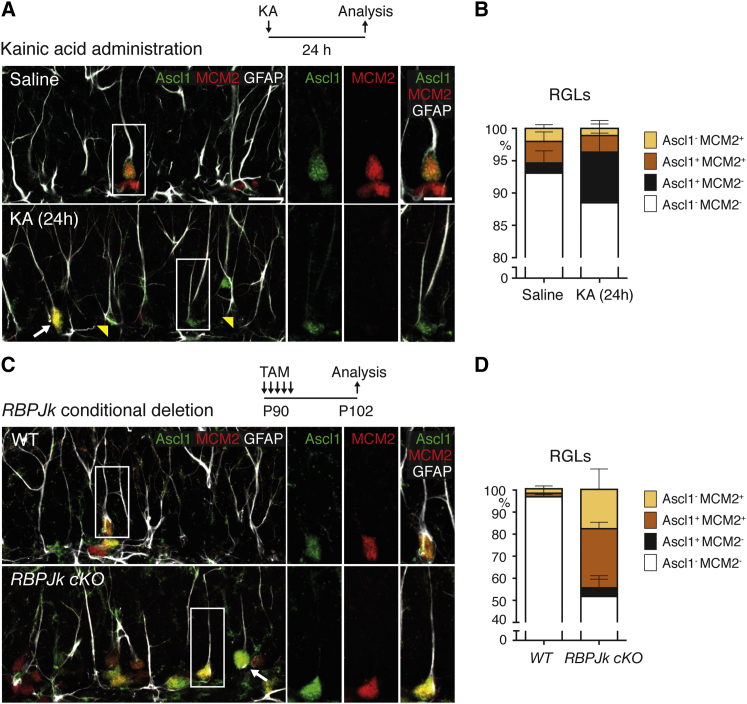
Induction of Ascl1 Expression in Hippocampal Stem Cells by Neurogenic Stimuli (A and B) Administration of kainic acid (KA) in 8- to 9-week-old mice and analysis of Ascl1, MCM2, and GFAP expression 24 hr later. The area boxed in (A), top (Saline), and enlarged on the right, and the white arrow in the bottom panel (KA) show an RGL that expresses Ascl1 and is activated (MCM2^+^). The area boxed in (A), bottom (KA), and the yellow arrowheads show Ascl1^+^ RGLs that are still quiescent (MCM2^−^). The percentages of RGLs expressing Ascl1 and MCM2 24 hr after injection (B) show an increased number of quiescent RGLs expressing Ascl1 in KA-injected mice, while the number of activated RGLs is not changed. p values, Ascl1^+^MCM2^−^ RGLs in saline versus KA = 0.0074; MCM2^+^ RGLs in saline versus KA = 0.6492. n = 4 (saline) and 5 (KA). (C and D) Inactivation of the *RBPJk* gene by injection of tamoxifen (TAM) in conditional *RBPJk*-mutant mice on 5 consecutive days starting at P90 and analysis of Ascl1, MCM2, and GFAP expression at P102 show that there are more Ascl1^+^ and activated RGLs in the DG of an *RBPJk cKO* mouse (arrow and boxed area in lower panel shown enlarged on the right) than in a control mouse (WT, enlarged area in upper panel) (C). The percentages of RGLs that express MCM2 and Ascl1 in the DG of WT and *RBPJk cKO* mice (D) show an increase in the number of quiescent Ascl1^+^ RGLs in *RBPJk cKO* mice (black bar), suggesting that blocking Notch signaling induces Ascl1 expression before RGLs exit quiescence. p values, Ascl1^+^ RGLs in WT versus *RBPJk cKO* < 0.0001; MCM2^+^ RGLs in WT versus *RBPJk cKO* < 0.0001; Ascl1^+^ MCM2^−^ RGLs in WT versus *RBPJk cKO* = 0.0911. n = 5 for WT, n = 7 for *RBPJk cKO*. Scale bars, 20 μm in main panels and 10 μm in enlarged panels. Values represent mean values, and error bars represent SDs.

The fact that Ascl1 expression is restricted to activated RGLs suggested that this factor might be induced by signals that promote RGL activity and neurogenesis in the hippocampus. To address this possibility, we examined Ascl1 expression in mice treated with the ionotropic glutamate receptor agonist kainic acid (KA), a neurogenic molecule that induces progenitor divisions in the DG (Lugert et al., 2010). A single injection of KA in 8- to 9-week-old wild-type (WT) mice produced, as expected, a robust increase in the number of MCM2^+^ RGLs in the DG, which became detectable 2 days after injection (Figure S2). Remarkably, KA induced Ascl1 expression in RGLs with more rapid kinetics, as the number of Ascl1^+^ RGLs was already increased 24 hr after injection of KA (Figure S2). Thus, a significant fraction of quiescent (MCM2^−^) RGLs expressed Ascl1 at 24 hr after injection (7.8% ± 1.3% Ascl1^+^ MCM2^−^ RGLs in KA-injected mice and 1.4% ± 0.9% in saline-injected mice at 24 hr; Figures 2A, 2B, and S2). The number of Ascl1^+^ quiescent RGLs decreased, while the number of activated RGLs increased at 2 days and 4 days (Figure S2). Together, these data indicate that KA administration induces Ascl1 expression in RGLs that are still quiescent. Therefore, Ascl1 induction precedes RGL activation.

To determine whether antineurogenic stimuli also regulate Ascl1 expression in the DG, we examined Notch signaling. Deletion of the Notch pathway component RBPJk has been shown to transiently induce the proliferation of hippocampal RGLs, followed later by a depletion of RGLs (Ehm et al., 2010). We deleted *RBPJk* from RGLs by injecting tamoxifen in 3-month-old mice carrying an inducible *RBPJk*-mutant allele (*Glast::CreERT2; RBPJk*^loxP/loxP^; *Rosa26R-stop-YFP* mice, called thereafter *RBPJk cKO* mice). Examination of the hippocampus 7 days later revealed a dramatic increase in the numbers of MCM2^+^ activated RGLs and of Ascl1-expressing RGLs in *RBPJk cKO* mice compared with control mice, demonstrating that loss of Notch signaling stimulates both the proliferation of RGLs and the expression of Ascl1 (Figures 2C and 2D). A fraction of RGLs in *RBPJk cKO* DGs expressed Ascl1, and not MCM2 (3.60% ± 1.50% in *RBPJk cKO* versus 0.25% ± 0.25% in control mice; Figure 2D), suggesting that like KA administration, inactivation of the Notch pathway in RGLs sequentially promotes Ascl1 expression and quiescence exit. We also examined the effect of voluntary exercise on RGL proliferation and expression of Ascl1 and found that it did not significantly increase proliferation of hippocampal RGLs, but only that of IPCs, as previously reported (Klempin et al., 2013). Together, our results demonstrate that neurogenic stimuli rapidly induce Ascl1 expression in quiescent RGLs, which in turn suggests that this factor might be implicated in RGL activation.

### *Ascl1* Is Absolutely Required for the Exit of RGLs from Quiescence

**Figure 3:**
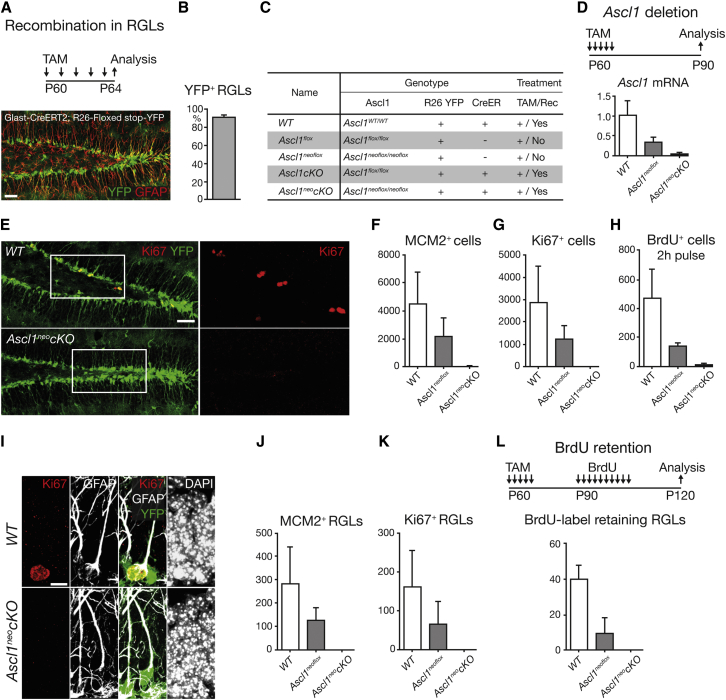
Block of Activation and Proliferation of Hippocampal Stem Cells by Conditional Inactivation of *Ascl1* (A and B) Administration of tamoxifen at P60–P64 in *Glast-CreERT2; Rosa26-floxed stop-YFP* mice followed by the analysis of YFP expression at P64 (A) and the percentage of RGLs that express YFP (B) show the high efficiency of recombination of the YFP reporter allele. n = 3. (C) Presentation of the five mouse lines analyzed in this study, including (first column) their names, (second column) the *Ascl1* allele they carry (WT, *flox*, or *neoflox*), (third column) the *Rosa26-floxed stop-YFP* reporter transgene they all carry (*R26 YFP*), (fourth column) whether they carry (+) or do not carry (−) the deleter allele *Glast-CreERT2* (*CreER*), and (fifth column) whether the tamoxifen injection they have all received (TAM, +) results in inactivation of *Ascl1* and the YFP reporter (Rec, Yes) or not (Rec, No). (D) Conditional inactivation of the *Ascl1* gene by tamoxifen administration at P60–P64 and analysis at P90 of *Ascl1* transcripts by quantitative RT-PCR on laser-capture-microdissected SGZ tissue shows that *Ascl1* expression is strongly reduced in *Ascl1*^neoflox^ mice compared to WT mice and is eliminated in *Ascl1*^neo^*cKO* mice. The graph shows expression levels normalized to *Gapdh* and relative to *Ascl1* expression in WT. n = 3 in each genotype. (E–H) Labeling for the proliferation marker Ki67 and for YFP to mark recombined cells (right panels are enlargements of the areas boxed in left panels) (E) and the total numbers per DG of MCM2^+^ cells (F), Ki67^+^ cells (G), and BrdU^+^ cells 2 hr after BrdU administration (H) show that cells do not proliferate in the DG of P90 *Ascl1*^neo^*cKO* mice, and that the lower level of *Ascl1* expression in *Ascl1*^neoflox^ mice results in reduced proliferation compared to WT mice. p values in WT versus *Ascl1*^neo^*cKO*, MCM2^+^ cells = 0.058; Ki67^+^ cells = 0.031; BrdU^+^ cells = 0.0162. n = 5 for WT, n = 4 for *Ascl1*^neo^*cKO* (F), 4 (G), and 3 (H). (I–L) Labeling for Ki67 and GFAP (I) and total numbers per DG of MCM2^+^ and Ki67^+^ RGLs (J and K) and BrdU^+^ cells following prolonged administration and chase of BrdU (L) demonstrate the absence of activated, proliferating, and BrdU label-retaining RGLs in *Ascl1*^neo^*cKO* mice and their reduced numbers in *Ascl1*^neoflox^ mice. p values in WT versus *Ascl1*^neo^*cKO*, MCM2^+^ cells = 0.016; Ki67^+^ cells = 0.038; BrdU^+^ cells = 0.0084. n = 5 for WT, n = 4 for *Ascl1*^neo^*cKO* (J), 4 (K), 5 (L, WT), 3 (L, *Ascl1*^neoflox^), and 3 (L, *Ascl1*^neo^*cKO*). Scale bars, 40 μm in (A) and (E) and 10 μm in (I). Values represent mean values, and error bars represent SDs.

To directly address the role of *Ascl1* in the activation of DG RGLs, we generated triple-transgenic mice that were homozygous for a conditional mutant allele of *Ascl1* (Pacary et al., 2011) and also carried the *Glast-CreERT2* allele to delete *Ascl1* in RGLs in a tamoxifen-dependent manner (Mori et al., 2006) and the *Rosa26-floxed stop-YFP* reporter transgene to identify cells having undergone Cre-mediated recombination by their expression of YFP (Srinivas et al., 2001). Administration of tamoxifen for 5 days to postnatal day 60 (P60) triple-transgenic mice and control mice (carrying *Glast-CreERT2* and *Rosa26-floxed stop-YFP*, but WT for *Ascl1*) resulted in widespread induction of YFP in radial GFAP^+^, Nestin^+^ RGLs (Figures 3A, 3B, and data not shown). However, examination by immunolabeling revealed that a fraction of SGZ cells that expressed YFP and had therefore recombined the *Rosa26-floxed stop-YFP* locus also expressed Ascl1 and had therefore not recombined the *Ascl1*^flox^ locus, indicating that recombination at the two loci was partially uncoupled (Vooijs et al., 2001). We also examined triple-transgenic mice carrying a different conditional mutant allele of *Ascl1*, in which a *PGK promoter-neo* cassette remained inserted on the 3′ side of the *Ascl1* locus (*Ascl1*^neoflox^ mice; Figures 3C and S3A). Interestingly, even without tamoxifen-induced recombination, *Ascl1* RNA and protein expressions were significantly reduced in the DG of these mice compared with WT mice (Figures 3D and S3B), suggesting that *Ascl1*^neoflox^ is a hypomorphic allele (Nagy et al., 1998). Analysis of *Ascl1*^neoflox^ mice at P10 did not reveal any overt morphological defect of the DG, and the rate of RGL proliferation was similar to that found in WT mice, indicating that the hypomorphic allele of *Ascl1* does not result in a developmental defect in the DG (Figure S3). Tamoxifen administration to these mice at P60–P64 resulted in undetectable *Ascl1* expression in the DG at P90 (*Ascl1*^neo^*cKO* mice; Figures 3C, 3D, S3A, and S3B). We therefore used *Ascl1*^neo^*cKO* mice in the rest of this study to examine the effect of loss of *Ascl1* on hippocampal neurogenesis, and we used *Ascl1*^neoflox^ mice to examine the effect of a reduced expression of *Ascl1*.

To determine whether *Ascl1* deletion has an impact on DG RGLs, we administered tamoxifen at P60–P64 and examined *Ascl1*^neo^*cKO* mice 1 month later, at P90. Double labeling for YFP and for MCM2, Ki67, or bromodeoxyuridine (BrdU) after a 2 hr pulse revealed a near-complete absence of proliferating YFP^+^ cells in the SGZ of *Ascl1*^neo^*cKO* mice (*Ascl1*^neo^*cKO* mice versus WT mice, 19.65 ± 19.65 versus 4,635 ± 921 YFP^+^ MCM2^+^ cells; 0 versus 2,875 ± 815 YFP^+^ KI67^+^ cells; 9.8 ± 4.9 versus 471.6 ± 115.5 YFP^+^ BrdU^+^ cells), while proliferating cells were present in *Ascl1*^neoflox^ mice, albeit in reduced numbers (2,233 ± 632 MCM2^+^ cells; 1,225 ± 298 KI67^+^ cells; 142.8 ± 10.2 BrdU^+^ cells; Figures 3E–3H). Moreover, triple labeling for YFP, GFAP, and MCM2 to mark activated stem cells or Ki67 to mark proliferating stem cells demonstrated a complete absence of recombined RGLs that were activated or proliferating in *Ascl1*^neo^*cKO* mice, while activated and cycling RGLs were present, but less numerous, in *Ascl1*^neoflox^ mice than in WT mice (*Ascl1*^neo^*cKO* versus *Ascl1*^neoflox^ versus WT mice, 0 versus 126.3 ± 26.3 versus 281.2 ± 70.1 MCM2^+^ cells; 0 versus 66.0 ± 29.5 versus 161.2 ± 48.5 KI67^+^ cells; Figures 3I–3K; see also Figures S3D–S3G for measures of proliferation in the DG of *Ascl1*^flox^ and *Ascl1cKO* mice). Rarely dividing RGLs, characterized by their capacity to retain BrdU, were labeled in tamoxifen-injected P90 mice by 10 days of BrdU administration followed by 20 days of chase (Figure 3L). BrdU label-retaining cells were present in WT mice and to a lesser extent in *Ascl1*^neoflox^ mice, but were again completely absent in *Ascl1*^neo^*cKO* mice (0 cells in *Ascl1*^neo^*cKO* mice; 9.7 ± 4.9 cells in *Ascl1*^neoflox^ mice; 43.8 ± 6.2 cells in WT mice; Figure 3L). We could thus demonstrate by several independent methods the complete inability of RGLs to exit quiescence and divide in the absence of *Ascl1*, and therefore establish that *Ascl1* is essential for activation of RGLs in the adult hippocampus. Interestingly, *Ascl1* deletion had no significant effect on the rate of RGL proliferation in the postnatal DG. When *Ascl1* was deleted by tamoxifen administration in *Ascl1*^neo^*cKO* mice at P7, RGLs continued to proliferate at P10 at a rate that was not significantly different from that seen in WT mice (Figure S3H). Therefore, the absolute requirement of *Ascl1* for RGL activity is specific to the adult DG.

Dividing RGLs in the adult hippocampus generate IPCs that proliferate before producing postmitotic granule neurons (Bonaguidi et al., 2011, Kempermann et al., 2004). Since RGLs require *Ascl1* to divide, the production of IPCs and their neuronal progeny might also depend on *Ascl1* function. The absence of Ki67^+^, MCM2^+^, and BrdU^+^ cells in the SGZ of *Ascl1*^neo^*cKO* mice (Figures 3F–3H) already suggested that IPCs are indeed missing in these mice. We further examined neurogenesis by double labeling the hippocampus of *Ascl1*^neo^*cKO* mice for YFP and for Tbr2 to mark IPCs, for DCX to mark neuroblasts, and for NeuN to mark granule neurons (Kempermann et al., 2004, Ming and Song, 2011). No YFP^+^ cells expressed these markers in the DG of *Ascl1*^neo^*cKO* mice at P90, demonstrating that no new IPCs or granule neurons were produced in these mice (Figures S3I–S3K; data not shown). *Ascl1* is therefore absolutely required for the generation of IPCs and for neurogenesis in the hippocampus.

### *Ascl1* Is Required for RGL Activation in the V-SVZ

**Figure 4:**
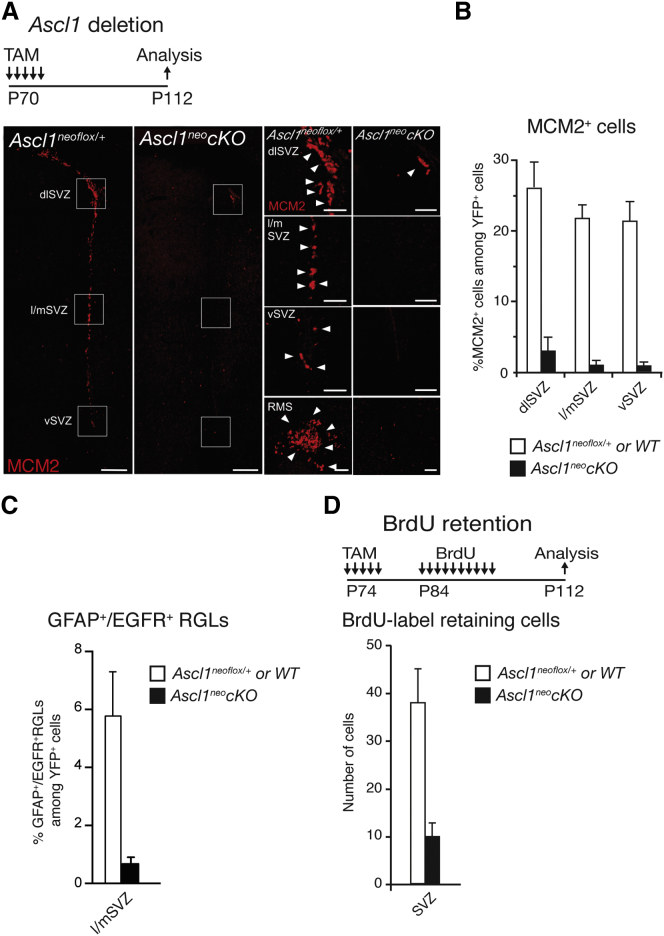
Block of Activation and Proliferation of V-SVZ Stem Cells by Conditional Inactivation of *Ascl1* (A–D) Labeling for the activation marker MCM2 (right panels are enlargements of the areas boxed in left panels; arrowheads point to MCM2^+^ cells) (A) and numbers of MCM2^+^ cells (B), activated RGLs identified by coexpression of GFAP and EGFR (C) among recombined YFP+ cells in the dorsal (dlSVZ), medial, lateral (l/mSVZ), and ventral (vSVZ) V-SVZ, and BrdU label-retaining cells in the whole SVZ following prolonged administration and chase of BrdU (D) show that RGLs are not activated and do not proliferate in the V-SVZ of P112 *Ascl1*^neo^*cKO* mice. p values in WT versus *Ascl1*^neo^*cKO*, MCM2^+^ cells = 0.00014 in dlSVZ, < 0.0001 in l/mSVZ, < 0.0001 in vSVZ; n = 5; GFAP^+^, EGFR^+^ cells = 0.00033; n = 4; BrdU label-retaining cells = 0.0056; n = 4. Scale bars, 200 μm in whole panels and 50 μm in enlarged panels. Values represent mean values, and error bars represent SDs.

We also examined neurogenesis in the V-SVZ of *Ascl1*^neo^*cKO* mice to determine whether the role of *Ascl1* in adult RGLs extends to the other main neurogenic region of the adult rodent brain. Analyzing the expression of GFAP, DCX, EGFR, and GFP in *Ascl1*^neo^*cKO* and WT mice showed that deletion of *Ascl1* results in a severe decrease in the fraction of RGLs of the V-SVZ that are activated (GFAP^+^ EGFR^+^) and proliferate (BrdU label retaining), and in a severe reduction in the production of DCX^+^ neuroblasts (Figures 4 and S4). Therefore, *Ascl1* is essential for NSC activation and proliferation and for neurogenesis in the two main neurogenic regions of the adult brain.

### *Ascl1* Acts Cell Autonomously to Promote RGL Proliferation

**Figure 5:**
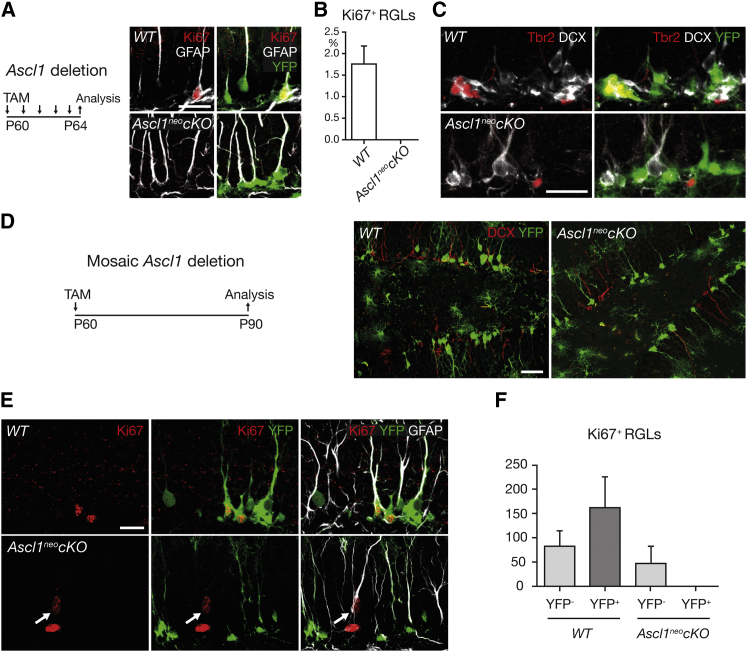
Cell-Autonomous Function of *Ascl1* in Hippocampal Stem Cell Proliferation (A–C) RGLs have ceased to proliferate in the *Ascl1*^neo^*cKO* DG as early as 4 days after the first tamoxifen injection (A and B). Percentages of RGLs expressing Ki67 in each genotype (B). Nonrecombined (YFP^−^) Tbr2^+^ IPCs and DCX^+^ neuroblasts remain in the DG of the *Ascl1*^neo^*cKO* mouse at P64 (C), but they cannot rescue the proliferation defect of recombined (YFP^+^) RGLs (B). p value, Ki67^+^ RGLs in WT versus *Ascl1*^neo^*cKO* = 0.0019. n = 3 in each genotype. (D–F) A single injection of tamoxifen results in recombination in only a fraction of RGLs, and nonrecombined RGLs produce YFP^−^ DCX^+^ neuroblasts in both WT and *Ascl1*^neo^*cKO* mice (D). In the *Ascl1*^neo^*cKO* DG, nonrecombined (YFP^−^, arrow) RGLs proliferate, but recombined (YFP^+^) RGLs do not, demonstrating that the presence of WT RGLs and their progeny does not rescue the proliferation defect of *Ascl1*-deficient RGLs (E and F). Total numbers of Ki67^+^ RGLs among YFP^+^ and YFP^−^ RGLs in the two genotypes (F). p value, Ki67^+^ YFP^+^ RGLs in WT versus *Ascl1*^neo^*cKO* = 0.0003. n = 3 (WT) and 6 (*Ascl1*^neo^*cKO*). Scale bars, 20 μm in (A), (C), and (E) and 40 μm in (D). Values represent mean values, and error bars represent SDs.

Ascl1 is expressed in proliferating RGLs, and the loss of *Ascl1* results in an arrest of RGL proliferation, suggesting that this gene is required in RGLs to promote their divisions. However, Ascl1 is also expressed in some IPCs, raising the alternative possibility that *Ascl1* is primarily required for the generation and/or division of IPCs, and that the arrest of RGL divisions is a secondary consequence of the loss of IPCs. In particular, a loss of IPCs might disrupt Notch signaling in the SGZ, resulting in a transient increase in RGL proliferation, followed later by a reduction of proliferation due to RGL exhaustion (Ables et al., 2010, Ehm et al., 2010, Lavado et al., 2010). To address this possibility, RGL proliferation was monitored in *Ascl1*^neo^*cKO* mice just 4 days after the beginning of tamoxifen administration at P60. RGLs had already stopped dividing in P64 *Ascl1*^neo^*cKO* mice, indicating that the effect of *Ascl1* deletion on RGL proliferation is rapid and therefore likely direct (Figures 5A and 5B). Moreover, although RGLs in the DG of P64 *Ascl1*^neo^*cKO* mice are mostly YFP^+^ and have therefore recombined (Figures 3B and 5A), WT Tbr2^+^ IPCs and DCX^+^ neuroblasts that were produced by RGLs before tamoxifen administration at P60–P64 are still present (Figure 5C). This suggests that the RGL proliferation defect is not the consequence of a loss of *Ascl1*-mutant IPCs and neuroblasts, i.e., *Ascl1* is required cell autonomously for RGL divisions.

To more rigorously address the cell autonomy or noncell autonomy of *Ascl1* function, we performed a mosaic analysis. We activated CreERT2 in a fraction of RGLs with only one injection of tamoxifen at P60, resulting 1 month later in an intermingling of recombined YFP-positive RGLs and nonrecombined YFP-negative RGLs and their progenies (Figure 5D). In WT mice that had received a single tamoxifen injection, a fraction of recombined YFP^+^ RGLs was proliferating (Figures 5E and 5F). In contrast, recombined YFP^+^ RGLs did not proliferate in mosaic *Ascl1*^neo^*cKO* and *Ascl1cKO* mice, irrespective of the recombination efficiency (Figures 5E, 5F, and S5). Therefore, the proliferation defect of *Ascl1*-mutant RGLs cannot be rescued by the presence of nearby WT cells, demonstrating that *Ascl1* is required cell autonomously in RGLs for their divisions.

### *Ascl1*-Deficient RGLs Do Not Respond to Mitogenic Stimuli

**Figure 6:**
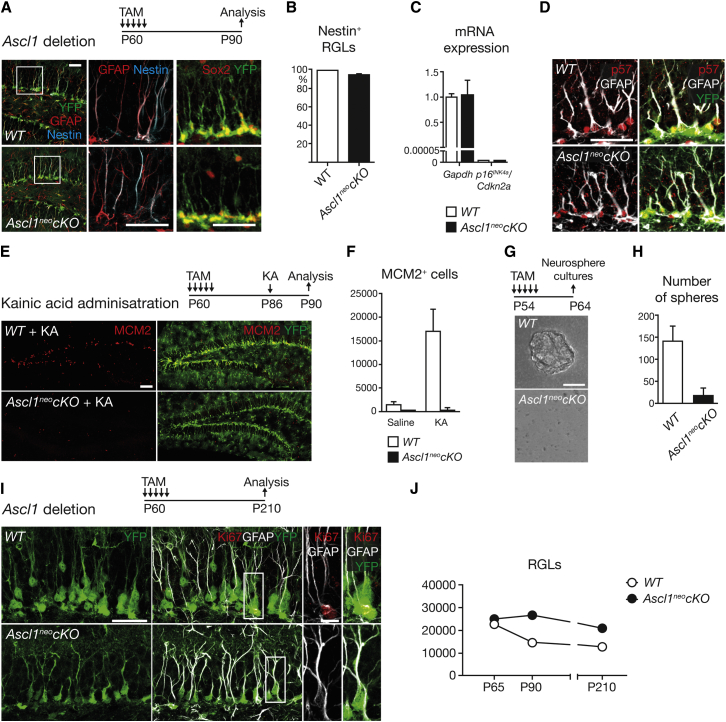
Unresponsiveness of *Ascl1*-Deficient Hippocampal Stem Cells to Extrinsic Stimuli (A and B) *Ascl1*-deficient RGLs in P90 *Ascl1*^neo^*cKO* mice retain a neural stem cell phenotype characterized by expression of the neural stem/progenitor cell markers Nestin, Sox2, and GFAP and a radial morphology. We note that Nestin remains expressed in *Ascl1*-mutant quiescent hippocampal RGLs, albeit at a lower level than in WT RGLs (not shown), whereas it is not expressed in quiescent RGLs in the V-SVZ (Codega et al., 2014). n = 3 in each genotype. (C) Quantitative RT-PCR analysis of the cyclin-dependent kinase inhibitor *p16*^INK4a^/*Cdkn2a* in the laser-capture-microdissected SGZ shows that expression of this marker of RGL senescence is barely detectable and not increased in the *Ascl1*^neo^*cKO* SGZ compared with WT mice. Expression levels normalized to *Gapdh*. n = 3 in each genotype. (D) Expression of the cyclin-dependent kinase inhibitor p57^Kip2^, a marker of quiescent RGLs, is similar in the DG of WT and *Ascl1*^neo^*cKO* mice. (E and F) KA administration stimulates cell proliferation in the SGZ of WT mice, but not in the SGZ of *Ascl1*^neo^*cKO* mice. Total numbers of MCM2^+^ cells in the SGZ in each condition (F). p value, MCM2^+^ cells in WT versus *Ascl1*^neo^*cKO* = 0.0183. n = 3 in each condition. (G and H) Clonal cultures of dissociated DG cells produced a large number of primary neurospheres from WT DG and only few neurospheres from *Ascl1*^neo^*cKO* DG. Total numbers of spheres obtained from clonal cultures of one dissociated DG. p value = 0.0060. n = 3 in each genotype. Further analysis showed that the few neurospheres from *Ascl1*^neo^*cKO* DG have escaped recombination and express Ascl1 (see Figure S6E). (I) At 5 months after *Ascl1* inactivation, SGZ cells in *Ascl1*^neo^*cKO* mice retain morphological and molecular features of RGLs and remain nonproliferative. The boxed areas are enlarged on the right and show a proliferating RGL in WT and a nonproliferating RGL in *Ascl1*^neo^*cKO*. Note the accumulation of YFP^+^ granule neurons above the SGZ in WT, and not in *Ascl1*^neo^*cKO*, mice. (J) RGL numbers decrease significantly with age in WT mice, while RGL numbers are not significantly different in *Ascl1*^neo^*cKO* mice at P65, P90, and P210. Total numbers of RGLs per DG. p values, WT RGLs at P65 versus P90 = 0.0176; at P90 versus P210 = 0.3655; *Ascl1*^neo^*cKO* RGLs at P65 versus P90 = 0.5453; at P90 versus P210 = 0.0525. n = 3 in each condition. Scale bars, 40 μm in (A), (D), (E), (G), and (I) and 10 μm in enlargement of (I). Values represent mean values, and error bars represent SDs.

Next, we examined the phenotype of the cells remaining in the SGZ after *Ascl1* deletion. Antibody labeling of the DG of P90 *Ascl1*^neo^*cKO* mice revealed that these cells retain the typical radial morphology of RGLs and maintain expression of the RGL markers GFAP, Nestin, and Sox2 and do not express the astrocytic marker S100β or the oligodendrocyte progenitor marker Olig2 (Figures 6A, 6B, and S6A–S6C). RT-PCR analysis showed that expression of *p16*^INK4a^*/Cdkn2a* was not elevated in the DG of *Ascl1*^neo^*cKO* mice, suggesting that RGLs in these mice do not become senescent (Molofsky et al., 2006). Moreover, RGLs in *Ascl1*^neo^*cKO* maintained normal levels of the cyclin-dependent kinase inhibitor p57^Kip2^, which is required for quiescence of hippocampal RGLs (Furutachi et al., 2013). These data, together with the lack of MCM2 expression in *Ascl1*-deficient RGLs (Figure 3J), show that *Ascl1* is not required for the maintenance of RGLs, but specifically for their activation, and that loss of *Ascl1* keeps RGLs in an inactive and undifferentiated state.

Although *Ascl1*-deficient RGLs are unable to proliferate when in a steady state, they might still be able to respond to a potent neurogenic stimulus such as KA. We therefore injected tamoxifen in *Ascl1*^neo^*cKO* mice at P60 to delete *Ascl1*, then injected KA at P86 and analyzed the DG at P90. KA failed to activate RGLs in *Ascl1*^neo^*cKO* mice, while it strongly activated them in WT mice (Figures 6E and 6F). Therefore, loss of *Ascl1* in RGLs results in an inactive state that cannot be reversed by KA stimulation. We also confirmed the inactivated state of *Ascl1*-deficient RGLs by performing an in vitro neurosphere assay in the presence of mitogens (Ehm et al., 2010). The DG was dissected from 7- to 8-week-old mice and dissociated, and single-cell suspensions were cultured at clonal density in the presence of FGF2 and EGF. Cultures of WT DG cells produced large numbers of primary neurospheres that generated secondary neurospheres when passaged. In contrast, *Ascl1*^neo^*cKO* DG cell cultures produced very few neurospheres, and antibody labeling showed that these neurospheres maintained expression of Ascl1 and therefore originated from cells that had escaped recombination (Figures 6G, 6H, and S6E). Therefore, *Ascl1*-deficient RGLs are quiescent and unable to respond to mitogens and divide in vitro.

We next examined whether the inactive state of *Ascl1*-deficient RGLs was stable over a longer period. Analysis of *Ascl1*^neo^*cKO* mice 5 months after *Ascl1* deletion, at P210, showed that RGLs retained a radial morphology and GFAP expression and remained Ki67 negative (Figure 6I). Interestingly, while the total number of RGLs in the DG of WT mice decreased considerably between P65 and P90 and decreased further at P210 (22,116 ± 1,681 at P65, 14,453 ± 1,021 at P90, and 12,516 ± 1,063 at P210), their number in *Ascl1*^neo^*cKO* mice did not change between P65 and P90 and decreased only slightly and nonsignificantly at P210 (24,533 ± 1,399 at P65, 26,098 ± 1,913 at P90, and 20,764 ± 395 at P210; Figure 6J). As the age-related attrition of hippocampal RGLs is thought to result from non-self-renewing divisions (Bonaguidi et al., 2012, Encinas et al., 2011), the maintenance of RGL numbers in older *Ascl1*^neo^*cKO* mice supports the finding that RGLs do not divide in these mice.

### *Ascl1* Directly Regulates Cell-Cycle Genes in Hippocampal Stem Cells

**Figure 7:**
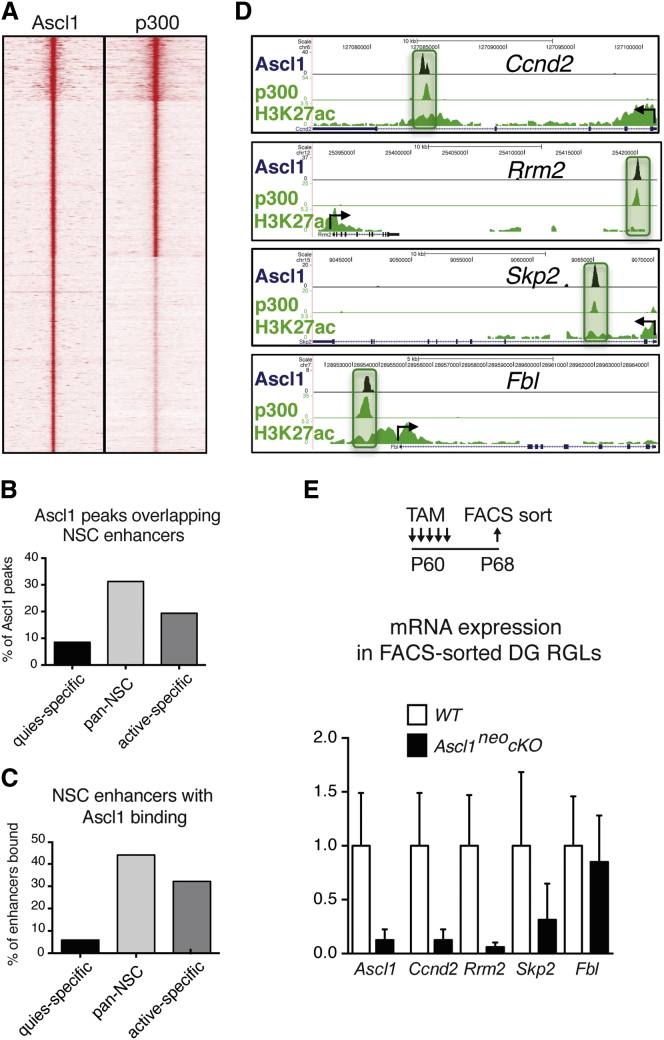
Direct Targets of Ascl1 in Adult Hippocampal Stem Cells (A–C) Heat map representation of Ascl1-binding signals in adult hippocampus-derived neural stem cells (AH-NSCs) alongside p300-binding signals in proliferating NSCs at the same genomic locations to mark enhancers (from Martynoga et al. [2013]) (A), and the distribution of Ascl1-binding sites in different classes of NSC enhancers (from Martynoga et al. [2013]) (B) shows that a large fraction of Ascl1-binding sites are located in enhancers present in proliferating NSCs, while reciprocally a large fraction of enhancers present in proliferating NSCs are bound by Ascl1 (C). quies-specific, enhancers specific for quiescent NSCs; pan-NSC, enhancers present in both quiescent and activated NSCs; active-specific, enhancers specific for activated NSCs (from Martynoga et al. [2013]). (D) ChIP-seq signals in AH-NSCs for Ascl1 and the enhancer marks p300 and H3K27ac (from Martynoga et al. [2013]) at five genes coding for components of the cell-cycle machinery. Significant binding peaks are indicated by green rectangles. Ascl1 binds enhancers in the four genes in AH-NSCs. (E) Quantitative RT-PCR analysis of fluorescent-activated cell-sorted YFP+ cells from the DG of WT and *Ascl1*^neo^*cKO* mice shows that expression of *Ascl1* and two Ascl1-bound genes, the cell-cycle genes *Ccnd2* and *Rrm2*, are strongly reduced in *Ascl1*^neo^*cKO* cells, while the expression of *Skp2* and *Fbl*, two other Ascl1-bound genes, is unchanged. The graph shows expression levels normalized to *Gapdh* and *Ppia* and relative to gene expression in WT. The remaining *Skp2* expression in *Ascl1*-mutant cells might be due to the presence of Glast^+^ parenchymal astrocytes in the sorted population. p values in WT versus *Ascl1*^neo^*cKO*, *Ascl1* = 0.0016; *Ccnd2* = 0.0016; *Rrm2* = 0.0011; *Skp2* = 0.052; *Fbl* = 0.65. n = 4 in each genotype. Values represent mean values, and error bars represent SDs.

To identify target genes that mediate the proliferative role of *Ascl1* in hippocampal stem cells, we examined the genome-wide binding of Ascl1 in adult hippocampus-derived NSCs (Knobloch et al., 2013) by chromatin immunoprecipitation-sequencing (ChIP-seq) with an anti-serum against Ascl1 (Figures 7 and S7). Ascl1 was bound to 7,826 high-confidence sites in the genome of AH-NSCs (Figure 7A), a majority of which mapped to enhancers previously identified in cultured NSCs (Figure 7B; Martynoga et al., 2013). Moreover, a large fraction of enhancers active in proliferating NSCs featured an Ascl1-binding event in AH-NSCs (Figure 7C).

We then established a list of the genes associated with an Ascl1-binding event in the AH-NSC genome (Table S1) and searched for candidate direct targets of Ascl1, i.e., genes that are both bound by Ascl1 and misregulated in RGLs of *Ascl1*^neo^*cKO*. We reasoned that since *Ascl1* is required for NSC proliferation in both DG and V-SVZ, it might regulate some of the same genes in the two tissues. We therefore intersected our list of Ascl1-bound genes with a recently published list of V-SVZ genes enriched in activated NSCs compared with quiescent NSCs (Figure S7; Table S2) (Codega et al., 2014). The 250 genes that are both bound by Ascl1 in AH-NSCs and enriched in activated V-SVZ NSCs include, in addition to known *Ascl1* targets such as the Notch ligands *Dll1* and *Dll3* (Castro et al., 2011), several cell-cycle regulators such as *E2f1*, *Ccnd2*, *Cdc6*, and *Skp2*. To establish whether these genes are regulated by *Ascl1* in DG RGLs, we used fluorescence-activated cell sorting to purify YFP^+^ cells from the DG of *Ascl1*^neo^*cKO* and WT mice 4 days after tamoxifen treatment, and we analyzed gene expression by qRT-PCR. We found that the cyclin *Ccnd2* and the ribonucleotide reductase subunit *Rrm2* were significantly downregulated when *Ascl1* was deleted in RGLs, while *E2f1* and *Cdc6* expression were not detectable in either WT or mutant cells, and expression of *Skp2* and non-cell-cycle genes such as *Fbl* were not significantly reduced (Figure 7E). Together, these data demonstrate that *Ascl1* controls the proliferation of hippocampal RGLs by directly activating the expression of *Ccnd2*, *Rrm2*, and possibly additional cell-cycle genes.

## Discussion

Stem cells in adult tissues respond to environmental signals by adjusting the production of mature cells to the needs of the tissue. Deciphering the pathways that link physiological stimuli to NSC activity requires characterization of the intrinsic machinery that controls stem cell activation. We show in this study that signals regulating hippocampal stem cell activity control the expression of Ascl1, and that this factor has an essential role in stem cell activation.

### Neurogenic and Antineurogenic Signals Converge on Ascl1 Expression

Ascl1 expression in the adult brain has often been described as being restricted to IPCs in the SVZ and DG (Lugert et al., 2012, Parras et al., 2004). We found, however, that Ascl1 is already expressed in proliferating RGLs in the hippocampus, in agreement with earlier studies (Breunig et al., 2007, Kim et al., 2011). Ascl1 expression is then presumably maintained by the IPCs that are produced when RGLs divide. Most Ascl1^+^ RGLs are activated, but only about a third of activated RGLs express Ascl1 detectably, which might be due to an oscillation of Ascl1 expression in activated RGLs as in embryonic neural progenitor cells (Imayoshi et al., 2013).

The results of our experiments with KA-injected mice suggest that neurogenic stimuli such as neuronal activity promote stem cell activation in the hippocampus by inducing Ascl1 expression in quiescent RGLs. The essential role of Ascl1 in the activation of adult NSCs suggests that other neurogenic signals controlling this step, including Wnt signals in the hippocampus (Jang et al., 2013, Qu et al., 2010, Qu et al., 2013, Seib et al., 2013) and VEGF in the V-SVZ (Calvo et al., 2011), might also act by inducing Ascl1 expression. Our results with *RBPJk* conditional mutant mice also suggest that antineurogenic stimuli, including the Notch pathway, suppress stem cell activity by repressing *Ascl1* expression in RGLs. The mechanism by which the Notch-RBPJk pathway represses *Ascl1* expression can be inferred from studies in the embryonic brain and in cancer cells, which have shown that the Notch-induced Hes factors directly repress the *Ascl1* gene (Kageyama et al., 2005). Moreover, antineurogenic signals may also target Ascl1 protein activity. The maintenance of hippocampal stem cell quiescence by BMPs (Mira et al., 2010) may involve an inactivation of Ascl1 protein by BMP effectors Id proteins, which are known to block Ascl1 activity by preventing its dimerization with E proteins (Nakashima et al., 2001). FoxO3, which acts downstream of the insulin/IGF-1 signaling pathway to maintain hippocampal stem cell quiescence, shares many targets with Ascl1 and may also function by antagonizing Ascl1 function (Webb et al., 2013). Inactivation of Ascl1 protein in RGLs that have begun to transcribe the *Ascl1* gene may be important to slow down the transition to an active state or to accelerate the return of active RGLs to quiescence.

Mice carrying the hypomorphic allele *Ascl1*^neoflox^ express *Ascl1* in the DG at a reduced level and retain only a small fraction of the proliferating RGLs found in WT mice (Figure 3). The finding that a change in *Ascl1* expression level translates into a change in the fraction of RGLs that proliferate suggests that extrinsic signals may fine-tune the rate of hippocampal neurogenesis by modulating the expression level of Ascl1, as shown for PDK1/Akt signaling, which regulates Ascl1 protein stability in the embryonic brain (Oishi et al., 2009).

### *Ascl1* Has a Crucial Role in Hippocampal Stem Cell Activation

A cell that exits quiescence and transits from the G_0_ to the early G_1_ phase of the cell cycle assembles a prereplication complex that contains minichromosome maintenance proteins, including MCM2. MCM2 expression therefore marks not only cycling cells, but also activated cells that have exited the quiescent state but not yet re-entered the cell cycle (Niu et al., 2011, Stoeber et al., 2001). The glutamate receptor agonist KA induces the expression of Ascl1 before that of MCM2, indicating that Ascl1 induction is one of the first steps in the pathway through which neuronal activity promotes the activation of quiescent RGLs. Since MCM2 is not expressed in *Ascl1*-deficient RGLs even after KA stimulation, *Ascl1* is also absolutely required for the quiescence exit of hippocampal RGLs.

Because *Ascl1*-deficient RGLs do not exit the quiescent state, it is not possible to ascertain whether *Ascl1* also regulates the cell-cycle progression of RGLs. However, *Ascl1* promotes the proliferation of progenitor cells in the embryonic brain (which do not enter quiescence), and it directly regulates the expression of cell-cycle regulatory genes in AH-NSCs, including *Ccnd2* and *Rrm2*, suggesting that it promotes the divisions of hippocampal RGLs in addition to their activation. *Ascl1* has previously been shown to promote cell proliferation in cancer cells and in the injured zebrafish retina by regulating Wnt-signaling genes (Osada et al., 2008, Ramachandran et al., 2011, Rheinbay et al., 2013). In glioblastoma cancer stem cells (GBM CSCs), Ascl1 has been shown to bind a site near the Wnt antagonist gene *Dkk1*, whose regulation mediates *Ascl1* activity in these cells (Rheinbay et al., 2013). In AH-NSCs, however, Ascl1 does not bind this site and many other sites bound in GBM CSCs, and reciprocally, many Ascl1-bound loci were found in AH-NSCs, and not in GBM CSCs, including sites near the cell-cycle regulators *E2f1*, *Ccna1*, *Ccnd2*, and *Skp2* (data not shown), suggesting that *Ascl1* controls the proliferation of hippocampal RGLs by regulating different genes from those regulated in cancer cells and the injured retina.

Transcriptomic and genetic studies suggest that NSCs undergo profound changes in their oxygen and lipid metabolisms and cell adhesion properties when they exit quiescence (Knobloch et al., 2013, Martynoga et al., 2013, Renault et al., 2009). Further characterization of *Ascl1* target genes in RGLs should elucidate whether *Ascl1* directly controls these physiological changes in addition to promoting cell-cycle re-entry and cell-cycle progression. *Ascl1* inactivation also blocks RGL activity in the V-SVZ. Whether Ascl1 acts through the same downstream mechanisms in the two adult neurogenic regions remains to be addressed.

In contrast with the complete lack of RGL activity in the adult DG in the absence of *Ascl1*, RGL proliferation was not significantly affected by *Ascl1* deletion in the early postnatal DG, supporting our earlier finding that Ascl1 is not required for DG morphogenesis during embryonic development (Galichet et al., 2008). Interestingly, cell proliferation in the DG becomes also increasingly dependent on the *Ascl1* target *Ccnd2* between early postnatal and adult stages (Ansorg et al., 2012). Therefore, a switch in the genetic control of DG RGL proliferation occurs during the first few weeks of life, with the activation of a mitogenic pathway involving *Ascl1* and *Ccnd2*.

*Ascl1*-deficient RGLs remain in *Ascl1*^neo^*cKO* mice for at least 5 months without dividing, differentiating, or dying. The permanent cell-cycle arrest can be readily explained by the cell-autonomous role of *Ascl1* in RGL activation. The lack of astrocyte differentiation may also be partially explained by the absence of proliferation, as astrogenesis is normally coupled to RGL divisions (Encinas et al., 2011). However, astrocytes can also be generated by direct differentiation of RGLs without cell division (Bonaguidi et al., 2011). In the embryonic brain, activation of Notch signaling by ligands presented by IPCs and young neurons switches neurogenic progenitors to an astrocytic fate (Namihira et al., 2009). In the hippocampal SGZ, *Ascl1* deficiency greatly reduces the expression of the Notch effectors *Hes1* and *Hes5* (Figure S6D), likely due to the elimination of Notch-ligand-presenting IPCs and young neurons. The resulting decrease in Notch activity may thus block the astrocytic differentiation of *Ascl1*-deficient RGLs.

Numerous transcription factors have been shown to stimulate the self-renewal of stem cells in tissues such as the blood and the skin (Akala and Clarke, 2006, Goldstein and Horsley, 2012). These factors often act by regulating multiple aspects of the biology of the stem cells; for example, by suppressing their differentiation, senescence, or apoptosis (Lieu and Reddy, 2009, Souroullas et al., 2009). Only a few factors, such as Gata3 in hematopoietic stem cells (Ku et al., 2012) and Runx1 in hair follicle stem cells (Osorio et al., 2008), have been proposed to primarily regulate adult stem cell divisions. In the brain, the orphan nuclear receptor Tlx promotes hippocampal NSC proliferation through induction of Wnt7a and repression of p21/WAF1 (Niu et al., 2011, Qu et al., 2010). However, how Tlx is regulated is currently not known. The function of Ascl1 of controlling adult stem cell activity in response to environmental signals is therefore so far unique in the adult brain. It is akin to that of MyoD in muscle satellite stem cells, which is expressed shortly after quiescent satellite cells have been activated, and which in turn induces the expression of the component of the prereplication complex Cdc6 (Zhang et al., 2010). Identifying the molecular pathways that control *Ascl1* expression at transcriptional and posttranscriptional levels will be important in order to learn how to manipulate hippocampal neurogenesis for therapeutic purposes.

## Experimental Procedures

### Animals

Mice were housed, bred, and treated according to the guidelines approved by the Home Office under the Animal (Scientific Procedures) Act 1986. All experimental procedures involving mice have been approved by the Animal Welfare and Ethical Review Panel of the National Institute for Medical Research. *RBPJk*^loxp/loxp^ animals were generated as previously described (Han et al., 2002) and bred to *Glast::CreERT2* BAC transgenic mice (Slezak et al., 2007). *Ascl1*^neoflox/neoflox^ mice, in which exon 1 of the *Ascl1* gene is flanked by loxP sites (Pacary et al., 2011), were bred with *Glast-CreERT2* knockin mice (Mori et al., 2006) and with *Rosa26-floxed stop-YFP* reporter mice (Srinivas et al., 2001) to generate *Glast-CreERT2*; *Ascl1*^neoflox^; *R26 YFP* mice, which are heterozygous for *Glast-CreERT2* and homozygous for *Ascl1*^neoflox^ and *R26 YFP*. Both *Glast::CreERT2* and *Glast-CreERT2* lines target both radial and horizontal astrocytes in the DG. In order to remove the *PGK promoter-neo* cassette from the *Ascl1* locus, *Ascl1*^neoflox^ animals were crossed with *actβ-Flp* mice (The Jackson Laboratory).

### Tamoxifen, BrdU, and KA Administration

For activation of the CreERT2 recombinase, P60 animals were administered intraperitoneally (i.p.) 4-hydroxytamoxifen (TAM) for 5 consecutive days. For mosaic experiments, P60 animals received a single TAM injection at the same concentration. All animals including WT and *Ascl1*^neoflox^ mice received TAM injections. To examine proliferating progenitors, mice received a single i.p. injection of BrdU 2 hr prior to tissue collection. To examine slowly dividing RGLs, mice received 5 daily BrdU injections followed by 5 consecutive days of BrdU-containing drinking water. Mice were sacrificed 20 days later. Male mice received KA as a single i.p. injection and were monitored for 90 min after KA injection. Animals that did not display rearing and falling were sacrificed 1, 2, or 4 days later and processed as described below.

### Tissue Preparation and Immunofluorescence

Animals were transcardially perfused with saline followed by 4% paraformaldehyde (PFA). Brains were postfixed with 4% PFA for 2 hr at 4°C and sectioned coronally at 40 μm with a vibratome. The immunofluorescence procedure and the primary and secondary antibodies are described in Supplemental Information.

### Microscopic Analysis and Quantification

Labeled cells were counted in every ninth 40 μm section through the entire rostrocaudal length of the DG (−0.82 mm to −4.24 mm from bregma). Counted cells were divided by the number of z planes counted to obtain the number of cells per 1 μm, and then multiplied by the total length of the DG. To count RGLs (GFAP^+^ radial cells), cells were deemed radial if the cell body clearly associated with a DAPI-positive nucleus was located in the SGZ and had a single radial process extending through at least two-thirds of the granule layer. In all figures, the cell numbers counted in WT and *Ascl1*^neo^*cKO* mice are numbers of YFP^+^ marker^+^ double-labeled cells, while the numbers counted in *Ascl1*^neoflox^ mice are for Ki67^+^ cells only, since YFP is not expressed in these mice.

### Laser-Capture Microdissection, FACS Sorting, RNA Isolation, and Quantitative Real-Time PCR

Coronal sections 14 μm long were cut from fresh-frozen brains in OCT with a cryostat and placed on slides. The SGZ of WT, *Ascl1*^neoflox^, and *Ascl1*^neo^*cKO* mice was excised by a PLAM laser-capture microdissection system (Zeiss) and collected in an adhesive cap. RNA from microdissected tissue was extracted and purified using Arcturus Pico Pure RNA Isolation Kit (Applied Biosystems) and reverse transcribed using the High-Capacity cDNA Reverse Transcription Kit (Applied Biosystems). Gene expression was detected using TaqMan Gene Expression Assays (Applied Biosystems). The protocol used for fluorescent-activated cell sorting is described in Supplemental Information.

### Statistical Analyses

Statistical analyses were conducted using a two-sample t test with equal variance in Prism software. Values represent mean values ± SD.

### Neurosphere Assay

Clonal primary and secondary neurosphere cultures were performed from dissociated DG dissected from 7- to 8-week-old mice as described (Walker et al., 2013). The number of neurospheres per well was counted 10 days after plating.

### ChIP-seq Data Generation and Processing

For chromatin immunoprecipitation, adult hippocampus-derived NSCs were fixed and processed as described (Castro et al., 2011) and immunoprecipitated using a rabbit anti-Ascl1 antibody (Abcam, ab74065, 4.5 μg per ChIP sample). DNA libraries were prepared and sequences analyzed as described (Martynoga et al., 2013). A total of 13.5 million nonredundant reads were used to call peaks, and only peaks with an FDR-corrected q value ≤ 1 × 10^−5^ were used for the analysis. p300 and H3K27ac data and active enhancer definitions in NSCs were from Martynoga et al. [2013]).

## Accession Numbers

The European Nucleotide Archive accession number for the Ascl1 ChIP-Seq data reported in this paper is PRJEB5023.

## Supplemental Information

Document S1. Figures S1–S7 and Supplemental Experimental Procedures

Table S1. Ascl1-Bound Genes

Table S2. Overlap of aNSCs in Codega et al. (2014) and Ascl1-Bound Genes

Document S2. Article plus Supplemental Information
