# Supplementary material for: A Transcriptional Mechanism Integrating Inputs from Extracellular Signals to Activate Hippocampal Stem Cells
Source: Neuron. 2014 Sep 3;83(5):1085–97. doi: 10.1016/j.neuron.2014.08.004 (PMC4157576; doi:10.1016/j.neuron.2014.08.004)
Supplement: Document S1. Figures S1–S7 and Supplemental Experimental Procedures [file mmc1.pdf]

Neuron, Volume *83*

Supplemental Information

A Transcriptional Mechanism Integrating Inputs from Extracellular Signals to Activate Hippocampal Stem Cells

Jimena Andersen, Noelia Urbán, Angeliki Achimastou, Ayako Ito, Milesa Simic, Kristy Ullom, Ben Martynoga, Mélanie Lebel, Christian Göritz, Jonas Frisén, Masato Nakafuku, and François Guillemot

Figure S1

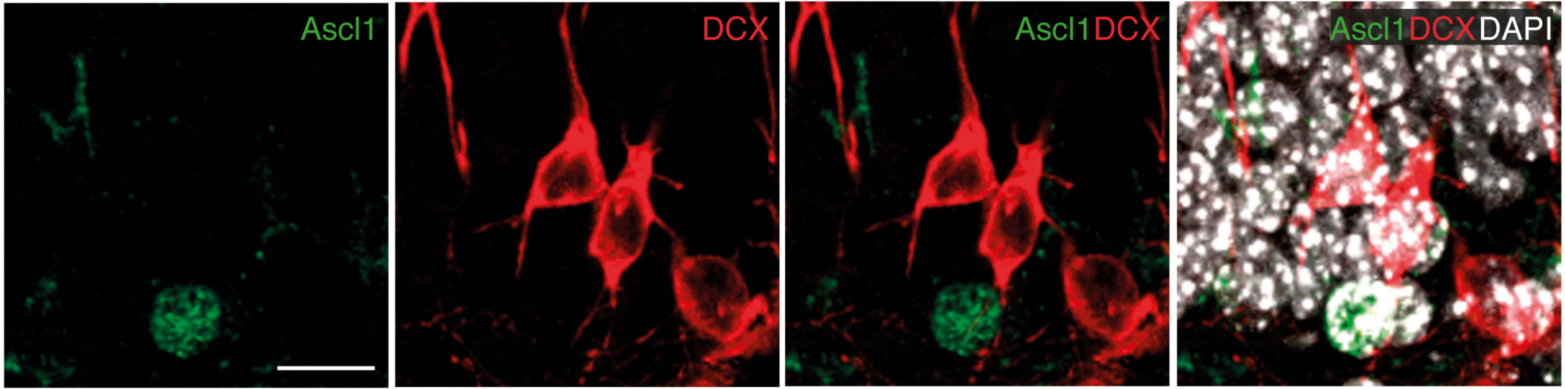

Figure S2

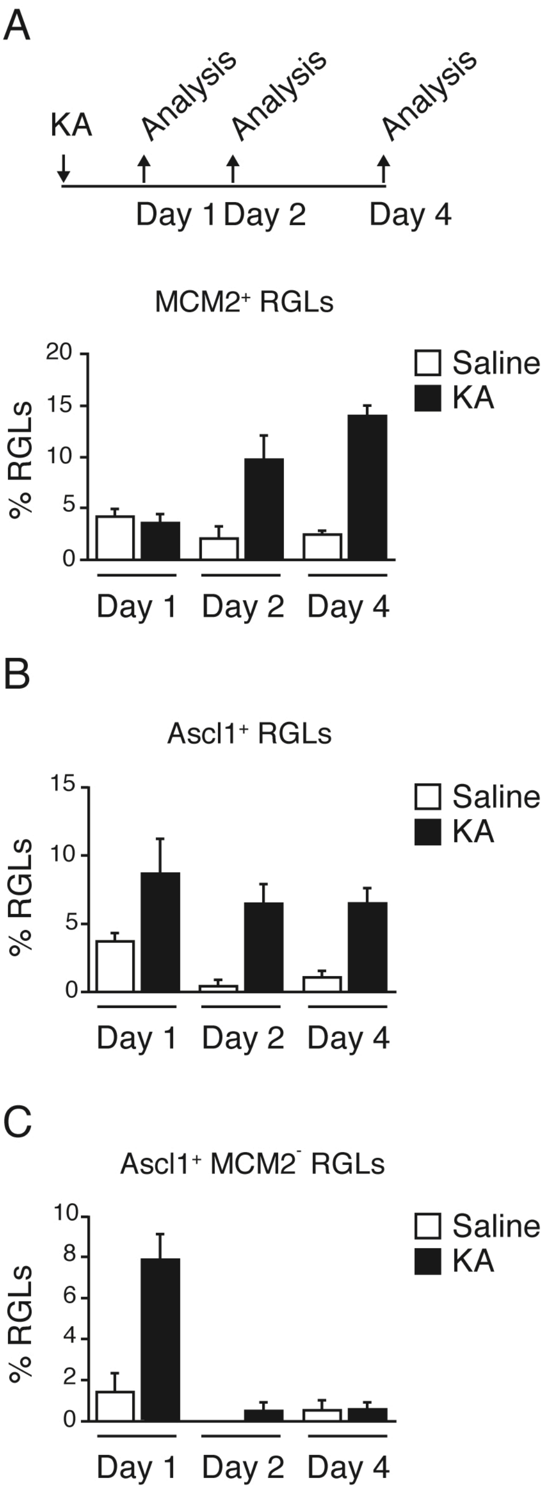
A
KA
Analysis
Analysis
Analysis
Day 1
Day 2
Day 4
MCM2+ RGLs
% RGLs
20
15
10
5
0
Saline
KA
Day 1
Day 2
Day 4
B
Ascl1+ RGLs
% RGLs
15
10
5
0
Saline
KA
Day 1
Day 2
Day 4
C
Ascl1+ MCM2- RGLs
% RGLs
10
8
6
4
2
0
Saline
KA
Day 1
Day 2
Day 4

Figure S3 A-H

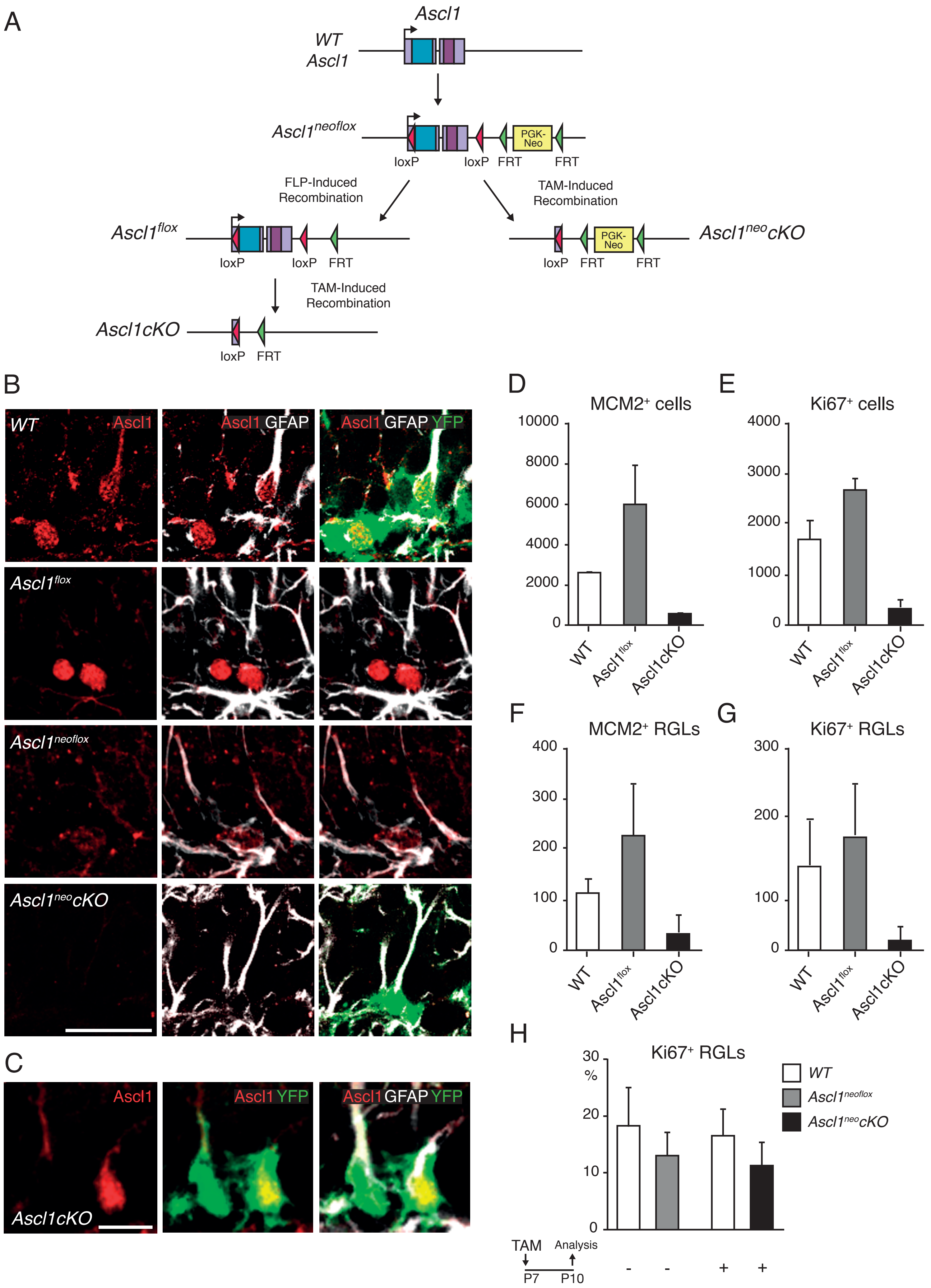
A
Ascl1
WT
Ascl1
Ascl1neoflox
PGK-
Neo
loxP
loxP
FRT
FRT
FLP-Induced
Recombination
TAM-Induced
Recombination
Ascl1flox
Ascl1neocKO
TAM-Induced
Recombination
Ascl1cKO
B
WT
Ascl1
Ascl1 GFAP
Ascl1 GFAP YFP
Ascl1flox
Ascl1neoflox
Ascl1neocKO
C
Ascl1
Ascl1 YFP
Ascl1 GFAP YFP
Ascl1cKO
D
MCM2+ cells
E
Ki67+ cells
F
MCM2+ RGLs
G
Ki67+ RGLs
WT
Ascl1flox
Ascl1cKO
H
Ki67+ RGLs
%
WT
Ascl1neoflox
Ascl1neocKO
TAM
Analysis
P7
P10
-
-
+
+

Figure S3 I-K

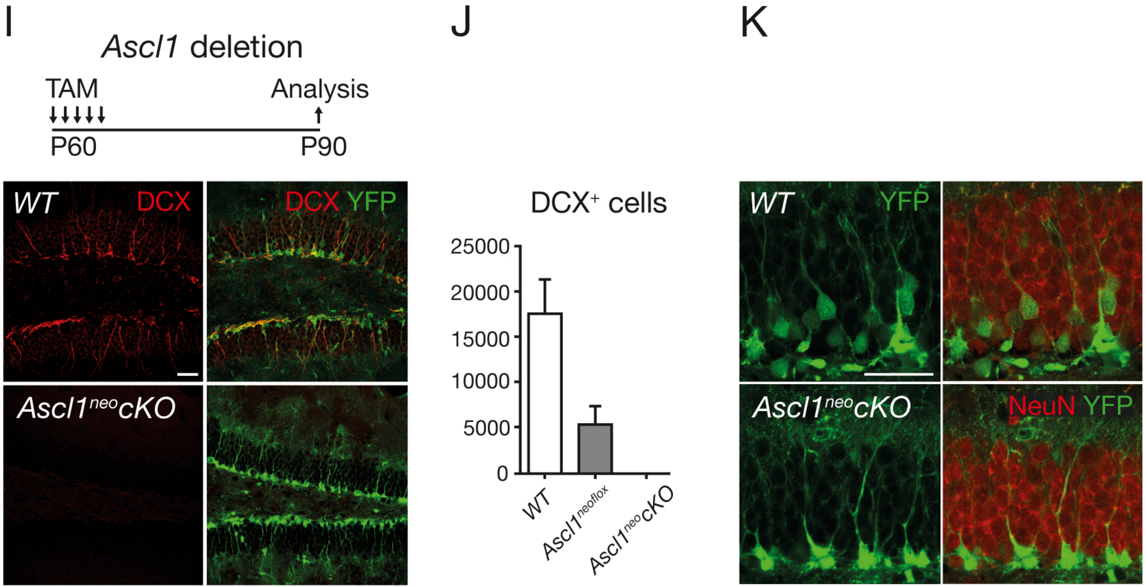
I
Ascl1 deletion
TAM
Analysis
P60
P90
WT
DCX
DCX YFP
Ascl1neocKO
J
DCX+ cells
25000
20000
15000
10000
5000
0
WT
Ascl1neoflox
Ascl1neocKO
K
WT
YFP
Ascl1neocKO
NeuN YFP

Figure S4

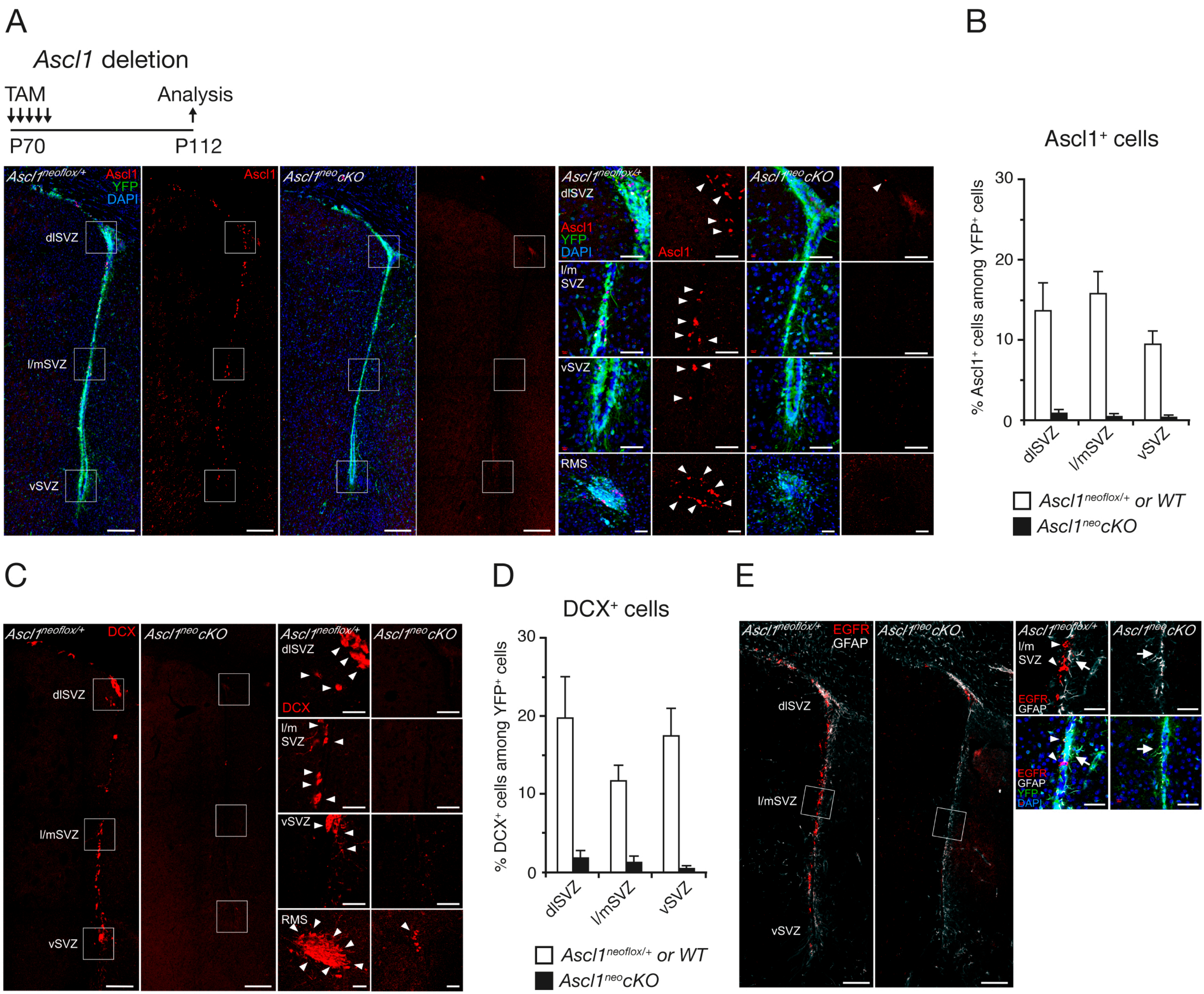
A
Ascl1 deletion
TAM
Analysis
P70
P112
Ascl1neoflox/+
Ascl1
YFP
DAPI
Ascl1neocKO
dlSVZ
l/mSVZ
vSVZ
l/m SVZ
RMS
B
Ascl1+ cells
% Ascl1+ cells among YFP+ cells
30
20
10
0
Ascl1neoflox/+ or WT
C
DCX
D
DCX+ cells
% DCX+ cells among YFP+ cells
E
EGFR
GFAP

Figure S5

A

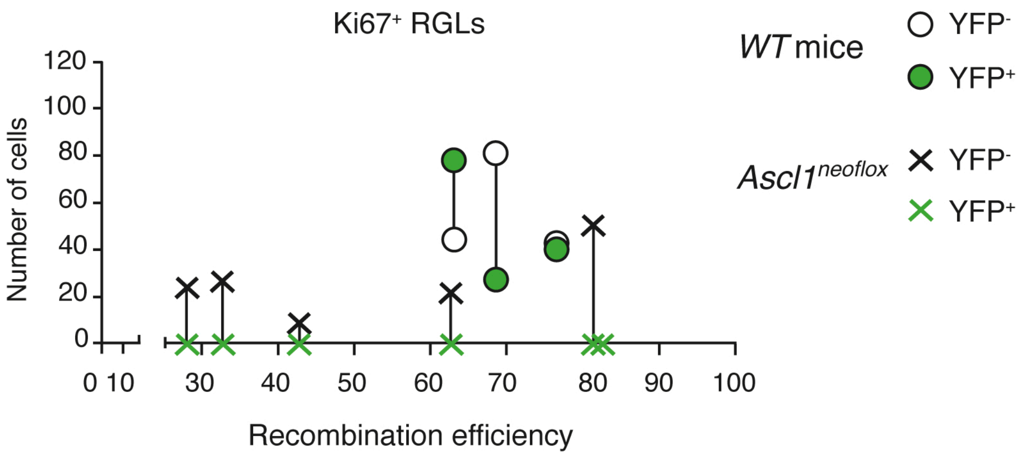
Ki67+ RGLs
WT mice
YFP-
YFP+
Ascl1neoflox
YFP-
YFP+
Number of cells
120
100
80
60
40
20
0
0 10
30
40
50
60
70
80
90
100
Recombination efficiency

B

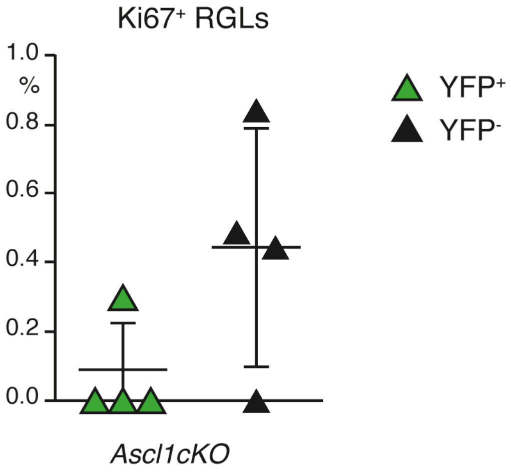
Ki67+ RGLs
YFP+
YFP-
%
1.0
0.8
0.6
0.4
0.2
0.0
Ascl1cKO

Figure S6

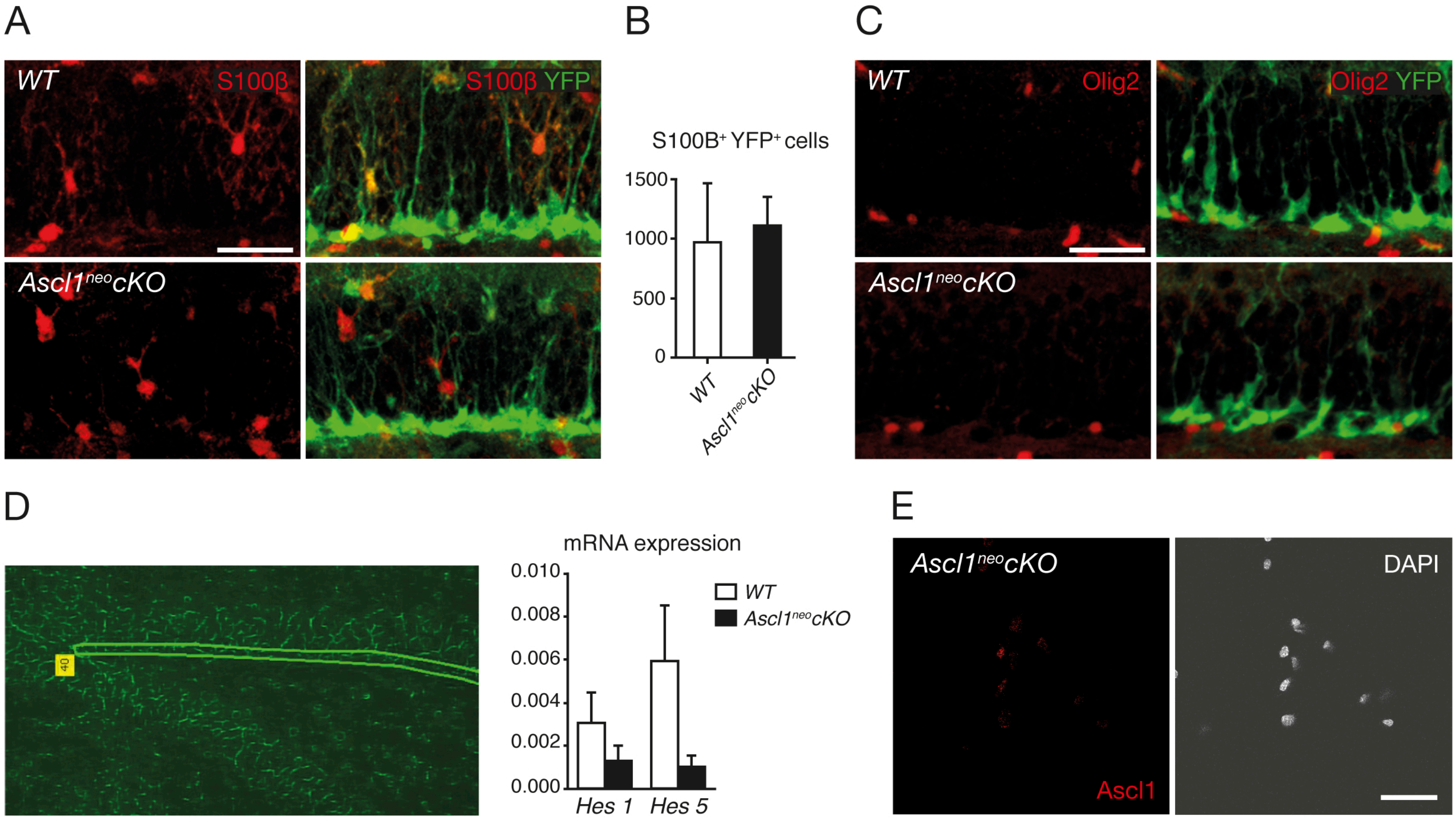
A
WT
S100β
S100β YFP
Ascl1neocKO
B
S100B+ YFP+ cells
1500
1000
500
0
WT
Ascl1neocKO
C
WT
Olig2
Olig2 YFP
Ascl1neocKO
D
40
mRNA expression
0.010
0.008
0.006
0.004
0.002
0.000
WT
Ascl1neocKO
Hes 1
Hes 5
E
Ascl1neocKO
Ascl1
DAPI

Figure S7

A

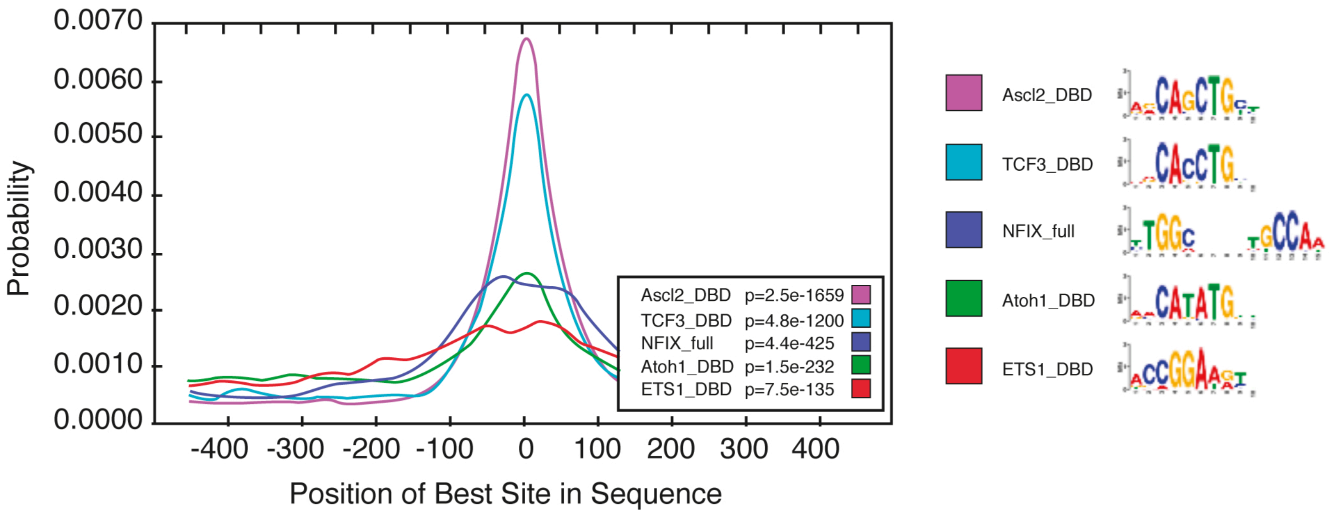

0.0070
0.0060
0.0050
0.0040
0.0030
0.0020
0.0010
0.0000
Probability
-400 -300 -200 -100 0 100 200 300 400
Position of Best Site in Sequence
Ascl2_DBD p=2.5e-1659
TCF3_DBD p=4.8e-1200
NFIX_full p=4.4e-425
Atoh1_DBD p=1.5e-232
ETS1_DBD p=7.5e-135
Ascl2_DBD
TCF3_DBD
NFIX_full
Atoh1_DBD
ETS1_DBD

B

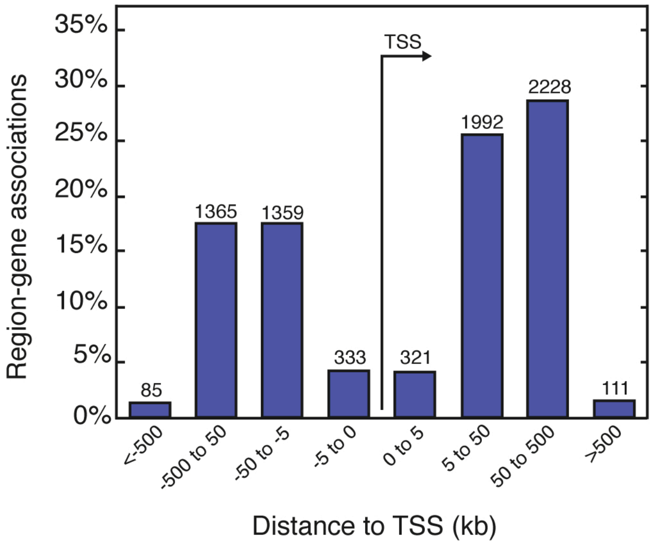

35%
30%
25%
20%
15%
10%
5%
0%
Region-gene associations
TSS
85
1365
1359
333
321
1992
2228
111
<-500
-500 to 50
-50 to -5
-5 to 0
0 to 5
5 to 50
50 to 500
>500
Distance to TSS (kb)

SUPPLEMENTAL INFORMATION

Supplemental Information includes six Supplemental Figures, Supplemental Experimental Procedures and Supplemental References.

Supplemental figure legends

Figure S1. Ascl1 is not expressed in DCX^+ neuroblasts

Labelling for Ascl1 and the neuroblast marker DCX in the DG of a P60 *WT* mouse shows that Ascl1 is not expressed by DG neuroblasts. Scale bar represents 20µm.

Figure S2. Induction of Ascl1 expression and stem cell activity in the DG of KA-injected mice

(A and B) The percentages of RGLs that express Ascl1 (A) or express MCM2 (B) 24 hours, 2 days and 4 days after saline and KA injections show that KA induces Ascl1 expression more rapidly than it promotes RGL activation. p-values: $MCM2^+$ RGLs in saline vs KA at 1 day = 0.6492, 2 days = 0.0578, 4 days = 0.0005; $Ascl1^+$ RGLs in saline vs KA at 1 day = 0.1567, 2 days = 0.0224, and 4 days = 0.0149. n = 3 (saline day 2 and day 4), 4 (saline day 1, KA day 2 and 4), 5 (KA day 1).

(C) The percentages of Ascl1-expressing quiescent ($MCM2^-$) RGLs 24 hours, 2 days and 4 days after saline and KA injections show that KA induces Ascl1 expression in RGLs that are still quiescent. p-values: $Ascl1^+MCM2^-$ RGLs in saline vs KA at 1 day = 0.0074, 2 days = 0.4366, 4 days = 0.9385. Values represent mean values, and error bars represent standard deviations.

Figure S3. Expression of Ascl1 and proliferation of SGZ cells and RGLs in *Ascl1^flox^* and *Ascl1cKO* mice

(A) Scheme showing how the different mutant alleles of *Ascl1* used in this study were generated.

(B) Labelling for Ascl1, GFAP and YFP shows that Ascl1 expression is markedly reduced in the non-recombined RGLs of $Ascl1^{neoflox}$ DG and is absent in recombined RGLs of $Ascl1^{neo}cKO$ DG.

(C) Labelling for Ascl1, YFP and GFAP in *Ascl1cKO* DG shows that some RGLs express YFP and have therefore recombined the *Rosa26 YFP* reporter allele but also express Ascl1 and have therefore not recombined the *Ascl1* gene.

(D to G) Total numbers per DG of SGZ cells (D, E) and RGLs (F, G) expressing MCM2 (D, F) and Ki67 (E, G) in WT, $Ascl1^{flox}$, and *Ascl1cKO* DG. Note the small number of proliferating SGZ cells and activated RGLs remaining in the *Ascl1cKO* DG due to the lack of complete recombination of the $Ascl1^{flox}$ allele. Note also the different numbers of proliferating RGLs found in *WT* and $Ascl1^{flox}$ mice, which might be due to expression of Cre in *WT* but not $Ascl1^{flox}$ RGLs (see Figure 3C). p-values: *WT* vs *Ascl1cKO*: $Mcm2^+$ cells = 0.0001, $Ki67^+$ cells = 0.0003; $Mcm2^+$ RGLs = 0.0254, $Ki67^+$ RGLs = 0.0108; $Ascl1^{flox}$ vs *Ascl1cKO*, $MCM2^+$ cells = 0.0005, $Ki67^+$ cells = 0.0001, $MCM2^+$ RGLs p = 0.0075, $Ki67^+$ RGLs = 0.0056. n = 3 in *WT* and $Ascl1^{flox}$ and n = 5 in *Ascl1cKO*.

(H) Percentages of proliferating RGLs in the DG of P10 *WT* and $Ascl1^{neoflox}$ mice, untreated with tamoxifen (left bars) and in the DG of P10 *WT* and $Ascl1^{neo}cKO$ mice

injected with tamoxifen at P7 (right bars). In contrast with the requirement for *Ascl1* in adult RGL proliferation, the hypomorphic allele of *Ascl1* (*Ascl1^{neoflox}*) and the deletion of *Ascl1* (*Ascl1^{neo}cKO*) do not significantly reduce RGL proliferation at this postnatal stage. p-values: *WT* vs *Ascl1^{neoflox}* = 0.2159. N = 5 for *WT* and n = 4 for *Ascl1^{neoflox}*. *WT* vs *Ascl1^{neo}cKO* = 0.2309. n = 3 for each.

(I and J) DCX^{+} neuroblasts are missing in the DG of *Ascl1^{neo}cKO* mice. Total numbers of DCX^{+} cells per DG. p-value: DCX^{+} cells in *WT vs Ascl1^{neo}cKO* = 0.0010. n = 3 in each genotype.

(K) The recently born YFP^{+} NeuN^{+} granule cells seen in the *WT* DG (green cells above the SGZ) are absent in the *Ascl1^{neo}cKO* DG. Scale bars represent 20μm in B, 10μm in C, and 40μm in I and K. Values represent mean values, and error bars represent standard deviations.

Figure S4. Block of activation and proliferation of V-SVZ stem cells by conditional inactivation of *Ascl1*

(A,B) Labelling for Ascl1 (A) and percentages of Ascl1^{+} cells among recombined YFP^{+} cells in the dorsal (dlSVZ), medial and lateral (l/mSVZ) and ventral (vSVZ) V-SVZ show the efficiency of *Ascl1* deletion in the V-SVZ of *Ascl1^{neo}cKO* mice. p-values in *WT vs Ascl1^{neo}cKO* Ascl1^{+} cells = 0.0015 in dlSVZ, = 0.0003 in l/mSVZ, = 0.0003 in vSVZ; n = 5.

(C,D) Labelling for the neuroblast marker DCX (C) and percentages of DCX^{+} cells among YFP^{+} cells in the dlSVZ, l/mSVZ and vSVZ (D) show that RGLs are not activated and do not generate neuroblasts in the V-SVZ of P112 *Ascl1^{neo}cKO* mice. p-

values in *WT vs Ascl1^{neo}cKO* DCX$^+$ cells = 0.0022 in dlSVZ, = 0.0004 in l/mSVZ, = 0.0004 in vSVZ; n = 5.

(E) Labelling for the RGL marker GFAP, the activation marker EGFR and YFP to identify recombined cells (right panels are enlargements of the areas boxed in left panels; arrowheads point to GFAP$^+$, EGFR$^+$ activated RGLs and arrows point to GFAP$^+$ EGFR$^-$ inactive RGLs). Scale bars represent 200µm in whole panels and 50µm in enlarged panels. Values represent mean values, and error bars represent standard deviations.

Figure S5. RGLs that have recombined in mosaic *Ascl1^{neo}cKO* and *Ascl1cKO* mice do not proliferate

(A) Mosaic analysis in *Ascl1^{neo}cKO* mice**.** For each *WT* and *Ascl1^{neo}cKO* mouse studied, the numbers of Ki67$^+$ RGLs (vertical axis) have been plotted separately for recombined (YFP$^+$) and non-recombined (YFP$^-$) RGLs and are shown linked by a vertical line. The recombination efficiency (horizontal axis) is the ratio of YFP$^+$ RGLs to total RGLs in each mouse. Note that recombined RGLs fail to proliferate in all the *Ascl1^{neo}cKO* mice.

(B) Mosaic analysis in *Ascl1cKO* mice**.** For each *WT* and *Ascl1cKO* mouse studied, the numbers of Ki67$^+$ RGLs have been plotted separately for recombined (green triangles) and non-recombined (black triangles) RGLs. Recombination efficiency was between 12% and 40% of all RGLs. Note that recombined RGLs fail to proliferate in the *Ascl1cKO* mice. Expression of Ki67$^+$ in one YFP$^+$ RGL is likely due to the uncoupling between recombination of *Ascl1* and of the *Rosa26 YFP* reporter

allele (Figure S3C, see Results section). Values represent mean values, and error bars represent standard deviations.

Figure S6. *Ascl1^{neo}cKO* RGLs remain undifferentiated and do not generate neurospheres

(A-C) Labelling for YFP and the astrocytic marker S100β (A) shows that *Ascl1^{neo}cKO* RGLs do not express this marker. The total numbers of YFP^{+} S100β^{+} astrocytes in the DG (B) show that there is no increase in number in astrocytes in *Ascl1^{neo}cKO* compared with *WT* mice. Labelling for YFP and the oligodendrocyte progenitor marker Olig2 shows that RGLs do not express this marker in *Ascl1^{neo}cKO* mice (C).

(D) Quantitative RT-PCR analysis in laser capture-microdissected SGZ tissue (top panel) of transcripts for the Notch transcriptional effectors *Hes1* and *Hes5* (bottom panel) shows that Notch signalling activity is reduced in the *Ascl1^{neo}cKO* SGZ. Expression levels normalized to *Gapdh.* n = 3.

(E) Clonal neurosphere cultures of dissociated DG cells shows that the few neurospheres that are generated from *Ascl1^{neo}cKO* DG contain Ascl1^{+} cells demonstrating that they originate from DG cells that did not undergo recombination. Scale bars represent 40μm. Values represent mean values, and error bars represent standard deviations.

Figure S7. Analysis of Ascl1-binding events in the genome of AH-NSCs

(A) Frequencies of motif occurrence around the summit of Ascl1-binding peaks in AH-NSCs. A bHLH factor binding E-box motif is found most frequently near Ascl1-binding peaks.

(B) Distribution of Ascl1-binding sites relative to transcription start sites (TSS) in the AH-NSC genome. Most Ascl1-binding sites are between 5 kb and 500 kb away from a TSS.

Supplemental experimental procedures

Animals

All procedures were performed in accordance with a UK Home Office Project Licence and approved by the local ethics committee. Mice were housed in standard cages under a 12 h light/dark cycle, and had *ad libitum* access to food and water. *RBPJk*$^{loxp/loxp}$ animals were generated as previously described (Han et al., 2002). *Glast-CreERT2* mice, which allow for tamoxifen-inducible expression of Cre recombinase under the astrocyte-specific glutamate aspartate transporter (GLAST, Mori et al., 2006) promoter, were crossed with *Ascl1*$^{neoflox/neoflox}$ mice, in which exon 1 of the *Ascl1* gene is flanked by loxP sites, and with *Rosa26-floxed stop-YFP* reporter mice (R26 YFP, Srinivas et al., 2001) to generate *Glast-CreERT2/Ascl1*^{neoflox}*/R26 YFP*. In order to remove the PGK promoter-neo cassette from the *Ascl1* locus, we crossed *Ascl1*^{neoflox} animals with an *actβ-Flp* mouse line (The Jackson Laboratories, Maine). Both male and female transgenic mice were included in the analysis.

Tamoxifen and BrdU administration

For activation of the CreERT2 recombinase, postnatal day 60 (P60) animals were administered 4- Hydroxytamoxifen (TAM, Sigma-Aldrich) for 5 consecutive days (intraperitoneally, ip; 2 mg/day, stock solution 10 mg/ml dissolved in 20% EtOH/80% sunflower oil). For mosaic experiments, P60 animals received a single TAM injection at the same concentration (based on preliminary studies with a range of concentrations that showed a single injection to produce adequate mosaic recombination). For deletion of *Ascl1* during postnatal life, animals received one

injection of TAM at P7 (i.p.; 50 µl, stock solution 20 mg/ml). Analysis was performed 3 days after TAM injection. All *WT*, *Ascl1^{neoflox} a*nd *Ascl1^{neo}cKO* animals received TAM injections to avoid injection-related differences between *Ascl1^{neoflox} a*nd experimental animals. To examine progenitor proliferation, mice were given a single bromodeoxyuridine (BrdU, Sigma-Aldrich) i.p. injection (2 mg, stock solution 10 mg/ml dissolved in 0.9% saline) 2 hours prior tissue collection. In order to examine slowly-dividing progenitor proliferation, a long-term BrdU paradigm was performed. Mice received 5 daily i.p. BrdU injections (2 mg/day) followed by 5 consecutive days of BrdU-containing drinking water (1 mg/ml). Mice were sacrificed 20 days after the last day of receiving BrdU in the drinking water.

Kainic acid administration

To induce seizures, 8-9 week-old MF1 mice or P86 *Ascl1 WT* and *Ascl1^{neo}cKO* male mice received kainic acid (KA, Sigma-Aldrich) as a single i.p. injection at 19 mg/kg dissolved in 0.9% saline (sub-seizure concentration based on preliminary studies showing that progenitor proliferation is induced without animals undergoing observable convulsions). Animals were monitored for 90 minutes after KA injection, and their behaviour was scored every 10 min from 0-7 based on (Monory et al., 2006) (0- no response, 1- immobility and staring, 2- forelimb and/or tail extension, rigid posture, 3- repetitive movements, 4- rearing and falling, 5- continuous rearing and falling, 6- severe clonic-tonic seizures, 7- death). Animals reaching stage 4 were immediately killed and were not used for the experiment. Around 15% of all animals receiving KA reached stage 4. Remaining animals were then sacrificed 1, 2

or 4 days after KA injection, and processed as described below.

Tissue preparation

Animals were terminally anesthetized and transcardially perfused with 0.9% saline for 3 min followed by 4% paraformaldehyde (PFA) in PBS for 12 min. Brains were post-fixed with 4% PFA for 2 hours at 4°C and sectioned coronally at 40μm using a vibratome (Leica). These sections were kept at 4°C on 0.02% Azide until used. For $p57^{Kip2}$ staining, brains were cryo-protected in sucrose overnight (30% sucrose in PBS) before being embedded in gelatin and sucrose (7.5 and 15%, respectively, in PBS) and frozen in isopentane. Frozen brains were cut in a Microm Cryostat (Zeiss) at 30μm and kept at minus 20°C until used.

Histology

For immunofluorescence staining, free-floating or frozen sections were blocked in 10% normal donkey serum and 1% Triton X-100 in PBS for 2 hours. Sections were then incubated overnight at 4°C with primary antibodies diluted at appropriate concentrations in incubation solution (10% normal donkey serum and 0.1% Triton X-100 in PBS). The following primary antibodies were used: mouse anti Ascl1 (1/100, BD Biosciences), rat anti BrdU (1/1000, AbD Serotec), goat anti doublecortin (1/50, Santa Cruz Biotechnology), rabbit anti GFAP (1/1000, Dako), rat anti GFAP (1/1000, Invitrogen Life Technologies), rabbit anti GFP (1/1000, Invitrogen Life Technologies), rat anti GFP (1/1000, Fine Chemicals), sheep anti GFP (1/1000, AbD Serotec), rabbit anti Ki67 (1/200, Leica), goat anti MCM2 (1/50, Santa

Cruz Biotechnology), mouse anti NeuN (1/1000, Chemicon), mouse anti Nestin (1/200, Millipore), rabbit anti S100β (1/500, Dako), goat anti Sox2 (1/500, Acris Antibodies), rabbit anti Olig2 (1/500, Millipore), rabbit anti $p57^{Kip2}$ (1/200, Sigma), and rabbit anti Tbr2 (1/500, Abcam). After 3 washes with PBT (PBS + 0.1% Triton X-100), sections were incubated in incubation solution with corresponding secondary antibodies for 2 hours at a dilution of 1/1000. Secondary antibodies used were conjugated to Alexa-488, Alexa-568 (Invitrogen Life Technologies), Cy3 or Cy5 (The Jackson Laboratory). Following 3 washes with PBT, sections were incubated with 6-diamidino-2-phenylindole (DAPI, 1/10000, Sigma-Aldrich) for 20 min to obtain nuclear staining, and finally mounted in Aqua PolyMount (Polysciences). For BrdU GFP GFAP triple staining, GFP GFAP staining was performed as explained above. Sections were then fixed for 30 min with 4% PFA, washed 3 times with PBS and then pre-treated with pre-warmed 2N HCL for 30 min. BrdU primary antibody was then added after two 15 min borate buffer (0.1 M sodium tetraborate, pH 8.5 in PBS) washes to continue immunostaining as normal. For immunocytochemistry of attached neurospheres, the same protocol was used except for the initial blocking step, which was performed with 10% normal donkey serum and 0.1% Triton X-100 in PBS for 30 minutes at room temperature.

Microscopic analysis and quantification

The total number of single, double or triple antigen-positive cells was counted in every ninth 40μm section through the entire rostrocaudal length of the DG (-0.82 mm to -4.24 mm from bregma). Images were acquired using an SP5 confocal

microscope (Leica). 10 to 15 z-plane images separated by a 1μm step were obtained and counted. To present total numbers per dentate gyrus, cells counted were divided by the number of z-planes counted to obtain the number of cells per 1μm, and then multiplied by the total length of the dentate gyrus. For counts of GFAP$^+$ RGLs, cells were deemed to be radial if the cell body, clearly associated with a DAPI-positive nucleus, was located in the SGZ and had a single radial process extending through at least the length of two cell bodies from the bottom of the subgranular zone. In each experiment the DG of 3 or more mice per group were analysed. In all figures, the cell numbers counted in *WT* and *Ascl1^{neo}cKO* mice are numbers of YFP$^+$ marker$^+$ double-labelled cells, while the numbers counted in *Ascl1^{neoflox}* mice are for marker$^+$ cells only since YFP is not expressed in these mice.

Laser Capture microdissection

For laser capture microdissection tissue collection, brains were removed after sacrifice, rapidly frozen in OCT on dry ice and stored at -80°C until further use. 14μm coronal sections spanning the length of the dentate gyrus were cut in a cryostat (CM3050S, Leica) and placed on MembraneSlides (Zeiss). The subgranular zone of *WT*, *Ascl1^{neoflox}* and *Ascl1^{neo}cKO* was excised by a PLAM laser-capture microdissection system (Zeiss) and collected in an adhesive cap (AdhesiveCap 200, ZEISS).

Fluorescence-activated cell sorting, RNA isolation and cDNA production and quantitative real-time PCR analysis

Glast-CreERT2, *Ascl1^{neoflox}*, *R26 YFP* and *Glast-CreERT2*, *Ascl1^{WT}*, *R26 YFP* mice were treated with tamoxifen during 5 days and the DG were microdissected 4 days after the last tamoxifen injection. The protocol used was as described (Walker et al., 2013), with the exception that un-stained pellets were re-suspended in 1 ml of DMEM:F12 without phenol red. The YFP+ cells were analyzed using a FACS Aria Cell Sorter (BD Biosciences). DGs of *Glast-CreERT2* negative animals were used for YFP gating and dead cells were excluded by propidium iodide staining (1ug/ml). The DGs of three to five animals of the same genotype were pooled for each sorting and considered as one n. RNA was extracted using the RNeasy Mini Kit (Qiagen) and following manufacturer's instructions. RNA was reverse transcribed using the High Capacity cDNA Reverse Transcription Kit (Applied Biosystems) following manufacturer's instructions. Gene expression was detected using TaqMan Gene expression assays (Applied Biosystems) as described by the manufacturer and performed on a 7500 real time PCR system (Applied Biosystems). Data were analysed using standard protocols to calculate relative expression with the dCT method with *Gapdh* serving as an endogenous control. Each probe was performed in duplicates for at least 3 independent samples per group.

Neurospheres

Primary neurospheres were obtained from 7 to 8 weeks old mice as previously described (Walker et al., 2013). Briefly, animals received a daily injection of

tamoxifen for five days and were killed four days after the last injection, the brains were removed and the DG were dissected. Isolated DG of each animal were processed separately. Tissue was enzymatically dissociated using the Neural Tissue Dissociation Kit (Milteny) according to manufacturer's instructions. The resulting cell suspension was diluted in HBSS with Calcium and Magnesium (Gibco, Life Technologies) and filtered through a 40µm cell sieve (Falcon; BD Bioscience). The single cell suspension was then pelleted and resuspended at a density of 1 dentate gyrus per P96-well plate. The neurosphere media consisted of DMEM:F12 (Gibco, Life Technologies), supplemented with NeuroCult NSC Proliferation Supplements (Stem Cell Techonologies), 2% bovine serum albumin (Sigma), 15mm potassium chloride (Sigma), 2µg/ml heparin (Sigma), 10ng/ml FGF2 (Peprotech) and 20ng/ml EGF (Peprotech). Cells were incubated for 12 days in a humidified incubator (5% CO_2) at 37°C and then the number of spheres formed was counted.

For passaging of the spheres, media was almost completely removed from the wells containing single spheres and 100 µl of trypsin EDTA (Gibco, Life Sciences) was added and incubated for 2 minutes at room temperature. Then an equal volume of trypsin inhibitor (Sigma) was added and neurospheres were mechanically dissociated with a P-200 tip. Cells were re-seeded in P24-well plates with 1ml of fresh medium. The number of neurospheres per well was counted 10 days after plating. Wells containing two or more spheres were considered as having successfully self-renewed. For immunocytochemistry analysis, the neurospheres were allowed to attach to a matrigel (Growth Factor reduced, BD Biosciences) -coated coverslip during 1 hour. They were then fixed with 4% PFA in PBS for 10

minutes and stored in PBS at 4 degrees until the immunocytochemistry was performed.

Statistical analysis

Statistical analyses were conducted using a two-sample t test with equal variance in Prism software. A p-value of <0.05 was considered significant. All values represent mean values, and error bars represent standard deviations.

ChIP-seq data generation and processing

Adult hippocampus-derived neural stem cells were a kind gift from S. Jessberger and were cultured on laminin-coated flasks in DMEM with Ham's F12, supplemented with N2 supplement plus EGF, FGF and heparin. For chromatin immunoprecipitation, cells were fixed sequentially with di(N-succimidyl) glutarate and 1% formaldehyde in phosphate-buffered saline and then lysed, sonicated and immunoprecipitated as described previously (Castro et al., 2011), using a rabbit anti-Ascl1 antibody (Abcam, ab74065, 4.5μg per ChIP sample).

DNA libraries were prepared from 20ng of immunoprecipitated DNA according to the standard Illumina ChIP-seq protocol. Libraries were sequenced with the Genome Analyzer IIx (Illumina). The raw reads were mapped to the mouse genome (mm9 including random chromosomes) with Bowtie2 (Langmead et al., 2012) version 2.1.0. An input chromatin sample was mapped in the same way and used as the control sample. Significant binding peaks were called using MACS (Zhang et al., 2008) version 2.0.10, after balancing the number of reads in treatment

and control samples, as described previously (Martynoga et al., 2013). 13.5 million non-redundant reads were used to call peaks and only peaks with an FDR-corrected q-value<=$1x10^{-5}$ were used for this analysis. p300 and H3K27ac data and active enhancer definitions in NSCs were from (Martynoga et al., 2013). Ascl1 binding events in GBMSCs were obtained from (Rheinbay et al., 2013) and for comparison of binding event locations were converted to the mouse mm9 genome co-ordinates with the UCSC liftOver tool.

Supplemental references

Castro, D.S., Martynoga, B., Parras, C., Ramesh, V., Pacary, E., Johnston, C., Drechsel, D., Lebel-Potter, M., Garcia, L.G., Hunt, C., *et al.* (2011). A novel function of the proneural factor Ascl1 in progenitor proliferation identified by genome-wide characterization of its targets. Genes Dev *25*, 930-945.

Han, H., Tanigaki, K., Yamamoto, N., Kuroda, K., Yoshimoto, M., Nakahata, T., Ikuta, K., and Honjo, T. (2002). Inducible gene knockout of transcription factor recombination signal binding protein-J reveals its essential role in T versus B lineage decision. Int Immunol *14*, 637-645.

Langmead, B., and Salzberg, S.L. (2012). Fast gapped-read alignment with Bowtie 2. Nat Methods *9*, 357-359.

Martynoga, B., Mateo, J.L., Zhou, B., Andersen, J., Achimastou, A., Urban, N., van den Berg, D., Georgopoulou, D., Hadjur, S., Wittbrodt, J., *et al.* (2013). Epigenomic

enhancer annotation reveals a key role for NFIX in neural stem cell quiescence. Genes Dev *27*, 1769-1786.

Monory, K., Massa, F., Egertova, M., Eder, M., Blaudzun, H., Westenbroek, R., Kelsch, W., Jacob, W., Marsch, R., Ekker, M.*, et al.* (2006). The endocannabinoid system controls key epileptogenic circuits in the hippocampus. Neuron *51*, 455-466.

Mori, T., Tanaka, K., Buffo, A., Wurst, W., Kuhn, R., and Gotz, M. (2006). Inducible gene deletion in astroglia and radial glia--a valuable tool for functional and lineage analysis. Glia *54*, 21-34.

Rheinbay, E., Suva, M.L., Gillespie, S.M., Wakimoto, H., Patel, A.P., Shahid, M., Oksuz, O., Rabkin, S.D., Martuza, R.L., Rivera, M.N.*, et al.* (2013). An Aberrant Transcription Factor Network Essential for Wnt Signaling and Stem Cell Maintenance in Glioblastoma. Cell Rep *3*, 1567-1579.

Srinivas, S., Watanabe, T., Lin, C.S., William, C.M., Tanabe, Y., Jessell, T.M., and Costantini, F. (2001). Cre reporter strains produced by targeted insertion of EYFP and ECFP into the ROSA26 locus. BMC Dev Biol *1*, 4.

Walker, T.L., Wierick, A., Sykes, A.M., Waldau, B., Corbeil, D., Carmeliet, P., and Kempermann, G. (2013). Prominin-1 Allows Prospective Isolation of Neural Stem Cells from the Adult Murine Hippocampus. J Neurosci *33*, 3010-3024.

Zhang, Y., Liu, T., Meyer, C.A., Eeckhoute, J., Johnson, D.S., Bernstein, B.E., Nusbaum, C., Myers, R.M., Brown, M., Li, W.*, et al.* (2008b). Model-based analysis of ChIP-Seq (MACS). Genome Biol *9*, R137.
