# Supplementary material for: A Transcriptional Mechanism Integrating Inputs from Extracellular Signals to Activate Hippocampal Stem Cells
Source: Neuron. 2014 Sep 3;83(5):1085–97. doi: 10.1016/j.neuron.2014.08.004 (PMC4157576; doi:10.1016/j.neuron.2014.08.004)
Supplement: Document S2. Article plus Supplemental Information [file mmc4.pdf]

CellPress

A Transcriptional Mechanism Integrating Inputs from Extracellular Signals to Activate Hippocampal Stem Cells

Jimena Andersen,[1] Noelia Urbán,[1,*] Angeliki Achimastou,[1] Ayako Ito,[1] Milesa Simic,[2] Kristy Ullom,[2] Ben Martynoga,[1] Mélanie Lebel,[1] Christian Göritz,[3] Jonas Frisén,[3] Masato Nakafuku,[2] and François Guillemot[1,*]
[1]Division of Molecular Neurobiology, MRC National Institute for Medical Research, Mill Hill, London NW7 1AA, UK
[2]Division of Developmental Biology, Cincinnati Children's Hospital Research Foundation, Cincinnati, OH 45229-3039, USA
[3]Department for Cell and Molecular Biology, Karolinska Institute, 17177 Stockholm, Sweden
*Correspondence: nurban@nimr.mrc.ac.uk (N.U.), fguille@nimr.mrc.ac.uk (F.G.)
http://dx.doi.org/10.1016/j.neuron.2014.08.004

SUMMARY

The activity of adult stem cells is regulated by signals emanating from the surrounding tissue. Many niche signals have been identified, but it is unclear how they influence the choice of stem cells to remain quiescent or divide. Here we show that when stem cells of the adult hippocampus receive activating signals, they first induce the expression of the transcription factor Ascl1 and only subsequently exit quiescence. Moreover, lowering *Ascl1* expression reduces the proliferation rate of hippocampal stem cells, and inactivating *Ascl1* blocks quiescence exit completely, rendering them unresponsive to activating stimuli. *Ascl1* promotes the proliferation of hippocampal stem cells by directly regulating the expression of cell-cycle regulatory genes. *Ascl1* is similarly required for stem cell activation in the adult subventricular zone. Our results support a model whereby *Ascl1* integrates inputs from both stimulatory and inhibitory signals and converts them into a transcriptional program activating adult neural stem cells.

INTRODUCTION

Adult stem cells maintain tissue function and integrity throughout the lifetime of an organism. They produce mature progenies to replace short-lived cells and repair tissue damage while maintaining their numbers through self-renewing divisions (Simons and Clevers, 2011). Many tissue stem cells are relatively quiescent, which delays their attrition and minimizes the accumulation of deleterious mutations (Orford and Scadden, 2008). The transit of stem cells between quiescent and activated states is not well understood in most systems. Elucidating the mechanisms that control the activation of tissue stem cells is an important goal in stem cell biology.

A variety of extracellular signals present in stem cell niches have been shown to influence the activity of tissue stem cells (Fuchs et al., 2004; Goldstein and Horsley, 2012; Kuang et al., 2008). For example, BMP signaling induces quiescence, while Wnts promote proliferation of skin and blood stem cells (Blank et al., 2008; Fuchs et al., 2004). However, the cell-intrinsic mechanisms that mediate the activity of extrinsic signals and promote stem cell quiescence or proliferation are poorly characterized. Niche signals might act by inducing the expression or activity of transcription factors that in turn regulate the large number of genes differentially expressed between quiescent and active stem cells (Lien et al., 2011; Martynoga et al., 2013; Venezia et al., 2004). Transcription factors have indeed been shown to regulate stem cell activity in various tissues by controlling their proliferation, survival, or differentiation (Akala and Clarke, 2006; Goldstein and Horsley, 2012). However, it is not known in most instances how these factors are regulated (Niu et al., 2011; Osorio et al., 2008).

In the adult mammalian nervous system, neural stem cells (NSCs) are found mostly in two regions of the anterior brain, the dentate gyrus (DG) of the hippocampus and the ventricular-subventricular zone (V-SVZ) lining the lateral ventricles, where stem cells produce new neurons that integrate into neuronal circuits of the hippocampus and olfactory bulb, respectively (Fuentealba et al., 2012; Ming and Song, 2011). Most adult NSCs are quiescent and rest in G_0, with only a small fraction progressing through the cell cycle at any time. NSC divisions result in the generation of transit-amplifying cells or intermediate progenitor cells (IPCs) that undergo a limited number of rapid divisions before they exit the cell cycle and differentiate into neurons (Ming and Song, 2011; Ponti et al., 2013). Clonal analysis in the adult mouse hippocampus in vivo has provided evidence that hippocampal NSCs, also called radial glia-like cells (RGLs), are multipotent and can generate both neurons and astrocytes, and that they use two modes of divisions to self-renew. Some RGLs divide asymmetrically to generate a new RGL and an IPC or an astrocyte, while others divide symmetrically into two new RGLs (Bonaguidi et al., 2011).

A particularly important feature of hippocampal neurogenesis is its regulation by a variety of physiological stimuli (Ming and Song, 2011). Neurogenesis in the hippocampus declines sharply with age, due in part to a reduction of the fraction of RGLs that divide, and it is suppressed by stress and depression (Lee et al., 2011; Ming and Song, 2011). Conversely, an enriched environment, task learning, or seizures stimulate hippocampal neurogenesis, in part by stimulating RGL divisions (Kronenberg

CrossMark

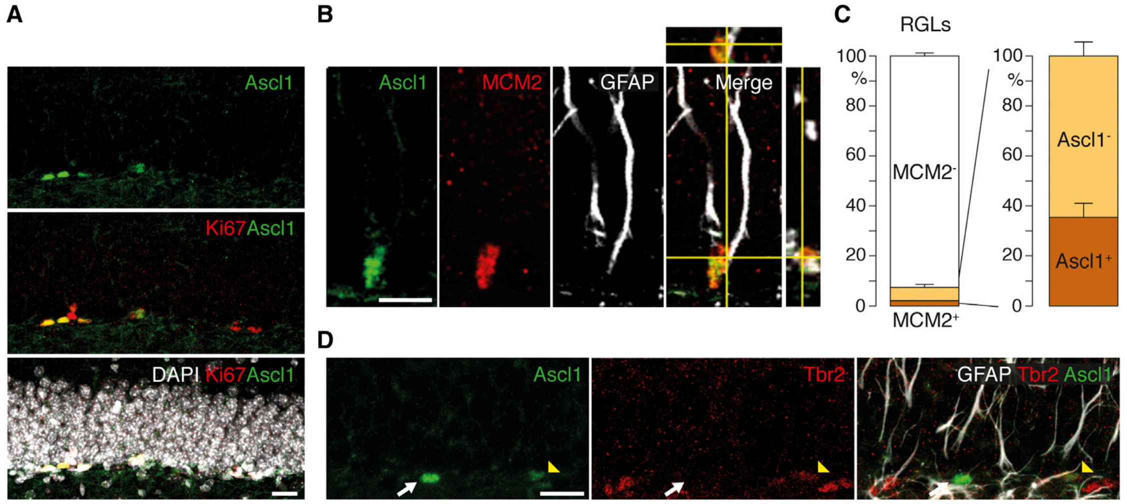

Figure 1. Expression of Ascl1 by Proliferating Stem and Progenitor Cells in the Adult Hippocampus

(A) Colocalization of Ascl1 protein (green) and proliferation marker Ki67 (red) shows that Ascl1 is expressed in proliferating cells in the dentate gyrus (DG) of a P60 mouse. Counterstained by DAPI (white).

(B) Ascl1 (green), MCM2 (a marker of cell cycle and G_0 to early G_1 transition, red), and neural stem cell marker GFAP (white) are colocalized in an activated radial glia-like stem cell (RGL). The z stack of the merged confocal picture is shown along the x axis (top) and the y axis (right).

(C) The percentage of activated (MCM2$^+$) and Ascl1-expressing (Ascl1$^+$) RGLs in the DG of P60 mice show that Ascl1 is expressed in a third of activated RGLs. n = 3.

(D) Labeling for Ascl1 (green), the intermediate progenitor cell (IPC) marker Tbr2 (red), and GFAP (white) in a P60 DG show that Ascl1 is expressed in both Tbr2$^+$ (yellow arrowhead) and Tbr2$^-$ (white arrow) GFAP$^-$ IPCs. Scale bars, 20 μm in (A) and (D) and 10 μm in (B). Values represent mean values, and error bars represent SDs.

et al., 2003; Ming and Song, 2011). Some of the extracellular signals that regulate RGL activity have been identified (Ming and Song, 2011). In particular, the BMP and Notch signaling pathways maintain RGLs in a quiescent state (Ables et al., 2010; Ehm et al., 2010; Mira et al., 2010), while the Wnt and IGF-1 pathways, among others, promote RGL divisions and stimulate neurogenesis (Bracko et al., 2012; Jang et al., 2013; Lie et al., 2005; Qu et al., 2010). Little is known, however, of how the activity of physiological stimuli or extrinsic signals is transduced inside RGLs to control their divisions. The orphan nuclear receptor Tlx is required to maintain RGLs in proliferation (Niu et al., 2011; Qu et al., 2010; Zhang et al., 2008), but how Tlx expression and activity are regulated has not been addressed.

The proneural transcription factor achaete-scute homolog 1 (Ascl1/Mash1) is an important regulator of neurogenesis in the embryonic nervous system. It is expressed by dividing progenitors and promotes their proliferation, specification, and differentiation into neurons (Bertrand et al., 2002; Castro et al., 2011). Moreover, ectopic expression of *Ascl1* can reprogram various cell types into neurons (Berninger et al., 2007; Yang et al., 2011). Ascl1 is also expressed in the DG and V-SVZ of the adult rodent brain, but its function there has not been examined. Ascl1 adult expression is mostly confined to IPCs (Lugert et al., 2012; Parras et al., 2004; Pastrana et al., 2009), but recent genetic lineage-tracing experiments have established that it is also present in self-renewing stem cells in both the V-SVZ and hippocampus (Kim et al., 2011). Consistent with this finding, Ascl1 was found expressed by a small subset of cycling stem cells in both neurogenic zones (Breunig et al., 2007; Kim et al., 2011). Here we show that Ascl1 expression is rapidly induced by neurogenic signals in hippocampal RGLs, and that *Ascl1* has a crucial role in RGL activation in both DG and V-SVZ. Ascl1 is specifically expressed in activated adult stem cells and is specifically required for the exit of stem cells from quiescence.

RESULTS

Ascl1 Is Expressed by Activated Stem Cells in the Adult Hippocampus

To study the function of *Ascl1* in hippocampal neurogenesis, we first characterized its expression in the adult hippocampus. Labeling of 2-month-old mouse brains with a monoclonal antibody against Ascl1 showed that in the hippocampus, Ascl1-expressing cells are restricted to the subgranular zone (SGZ) of the DG (Figure 1A). Double labeling for Ascl1 and the cell proliferation marker Ki67 showed that most Ascl1-expressing cells are proliferating (Figure 1A). Double labeling for Ascl1 and markers of progenitor cells of the DG neurogenic lineage showed that Ascl1 is expressed by three distinct progenitor cell populations. It is expressed by a small subset of RGLs, identified by their radial morphology and expression of GFAP. Most Ascl1-positive RGLs are activated, as 83.3% ± 16.7% of them express the cell cycle and cell activation marker MCM2 (Machida et al., 2005; Niu et al., 2011) (Figures 1B and 1C). Ascl1-positive RGLs represent 35.6% ± 3.2% of activated (MCM2$^+$) RGLs and 2.0% ± 0.7% of all RGLs (Figure 1C). Ascl1 is also expressed by nonradial GFAP$^+$ cells in the SGZ, which are also considered to be hippocampal NSCs (Lugert et al., 2010; Suh et al., 2007),

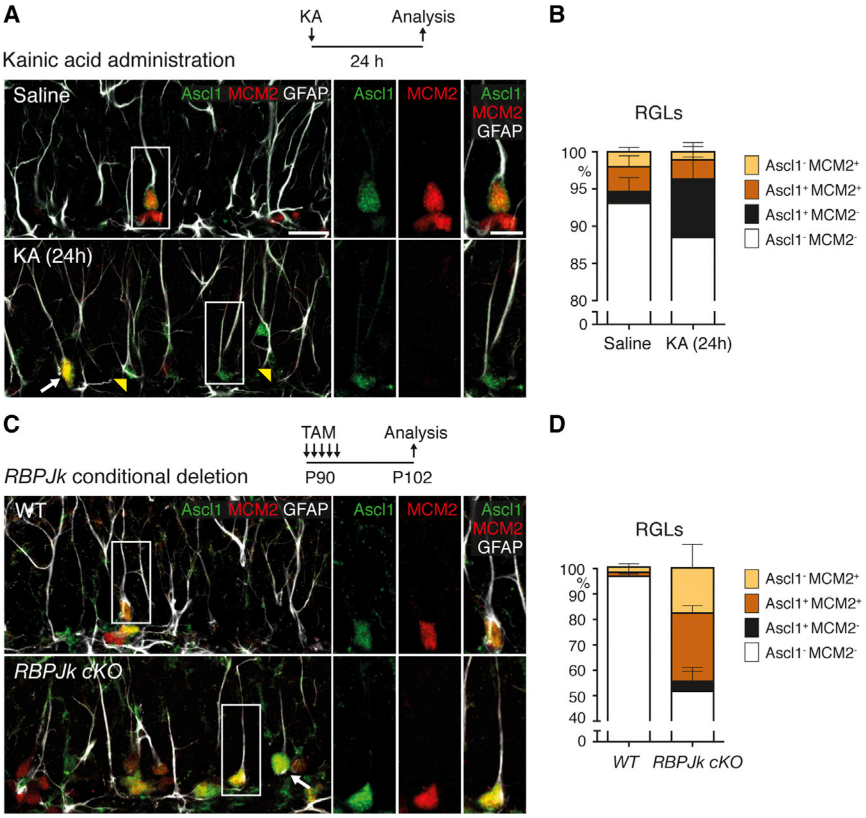

Figure 2. Induction of Ascl1 Expression in Hippocampal Stem Cells by Neurogenic Stimuli

(A and B) Administration of kainic acid (KA) in 8- to 9-week-old mice and analysis of Ascl1, MCM2, and GFAP expression 24 hr later. The area boxed in (A), top (Saline), and enlarged on the right, and the white arrow in the bottom panel (KA) show an RGL that expresses Ascl1 and is activated (MCM2$^+$). The area boxed in (A), bottom (KA), and the yellow arrowheads show Ascl1$^+$ RGLs that are still quiescent (MCM2$^-$). The percentages of RGLs expressing Ascl1 and MCM2 24 hr after injection (B) show an increased number of quiescent RGLs expressing Ascl1 in KA-injected mice, while the number of activated RGLs is not changed. p values, Ascl1$^+$MCM2$^-$ RGLs in saline versus KA = 0.0074; MCM2$^+$ RGLs in saline versus KA = 0.6492. n = 4 (saline) and 5 (KA).

(C and D) Inactivation of the *RBPJk* gene by injection of tamoxifen (TAM) in conditional *RBPJk*-mutant mice on 5 consecutive days starting at P90 and analysis of Ascl1, MCM2, and GFAP expression at P102 show that there are more Ascl1$^+$ and activated RGLs in the DG of an *RBPJk cKO* mouse (arrow and boxed area in lower panel shown enlarged on the right) than in a control mouse (WT, enlarged area in upper panel) (C). The percentages of RGLs that express MCM2 and Ascl1 in the DG of WT and *RBPJk cKO* mice (D) show an increase in the number of quiescent Ascl1$^+$ RGLs in *RBPJk cKO* mice (black bar), suggesting that blocking Notch signaling induces Ascl1 expression before RGLs exit quiescence. p values, Ascl1$^+$ RGLs in WT versus *RBPJk cKO* < 0.0001; MCM2$^+$ RGLs in WT versus *RBPJk cKO* < 0.0001; Ascl1$^+$ MCM2$^-$ RGLs in WT versus *RBPJk cKO* = 0.0911. n = 5 for WT, n = 7 for *RBPJk cKO*. Scale bars, 20 μm in main panels and 10 μm in enlarged panels. Values represent mean values, and error bars represent SDs.

and by IPCs, characterized by their SGZ location, proliferative state, and lack of GFAP expression (Figure 1D). Ascl1-positive IPCs represent 13.5% ± 4.1% of all IPCs and are subdivided into two subsets that differ in the expression of the IPC marker Tbr2 (Kempermann et al., 2004; Ming and Song, 2011). Ascl1 is not expressed by more mature cells in the lineage, including doublecortin (DCX)$^+$ neuroblasts and NeuN$^+$ granule neurons (Figure S1; data not shown). Together, these data agree with previous reports showing that Ascl1 expression is restricted to the earliest stages of the neurogenic lineage of the adult DG, including proliferating RGLs (Breunig et al., 2007; Kim et al., 2011) and early IPCs (Lugert et al., 2010, 2012), and that it is downregulated before IPCs begin to express neuronal markers and exit the cell cycle.

Ascl1 Expression in Hippocampal Stem Cells Is Induced by Neurogenic Stimuli

The fact that Ascl1 expression is restricted to activated RGLs suggested that this factor might be induced by signals that promote RGL activity and neurogenesis in the hippocampus. To address this possibility, we examined Ascl1 expression in mice treated with the ionotropic glutamate receptor agonist kainic acid (KA), a neurogenic molecule that induces progenitor divisions in the DG (Lugert et al., 2010). A single injection of KA in 8- to 9-week-old wild-type (WT) mice produced, as expected, a robust increase in the number of MCM2$^+$ RGLs in the DG, which became detectable 2 days after injection (Figure S2). Remarkably, KA induced Ascl1 expression in RGLs with more rapid kinetics, as the number of Ascl1$^+$ RGLs was already increased 24 hr after injection of KA (Figure S2). Thus, a significant fraction of quiescent (MCM2$^-$) RGLs expressed Ascl1 at 24 hr after injection (7.8% ± 1.3% Ascl1$^+$ MCM2$^-$ RGLs in KA-injected mice and 1.4% ± 0.9% in saline-injected mice at 24 hr; Figures 2A, 2B, and S2). The number of Ascl1$^+$ quiescent RGLs decreased, while the number of activated RGLs increased at 2 days and 4 days (Figure S2). Together, these data indicate that KA administration induces Ascl1 expression in RGLs that are still quiescent. Therefore, Ascl1 induction precedes RGL activation.

To determine whether antineurogenic stimuli also regulate Ascl1 expression in the DG, we examined Notch signaling. Deletion of the Notch pathway component RBPJk has been shown to transiently induce the proliferation of hippocampal RGLs, followed later by a depletion of RGLs (Ehm et al., 2010). We deleted *RBPJk* from RGLs by injecting tamoxifen in 3-month-old mice carrying an inducible *RBPJk*-mutant allele (*Glast::CreERT2; RBPJk*$^{loxP/loxP}$*; Rosa26R-stop-YFP* mice, called thereafter *RBPJk cKO* mice). Examination of the hippocampus 7 days later revealed a dramatic increase in the numbers of MCM2$^+$ activated RGLs and of Ascl1-expressing RGLs in *RBPJk cKO* mice

compared with control mice, demonstrating that loss of Notch signaling stimulates both the proliferation of RGLs and the expression of Ascl1 (Figures 2C and 2D). A fraction of RGLs in *RBPJk cKO* DGs expressed Ascl1, and not MCM2 (3.60% ± 1.50% in *RBPJk cKO* versus 0.25% ± 0.25% in control mice; Figure 2D), suggesting that like KA administration, inactivation of the Notch pathway in RGLs sequentially promotes Ascl1 expression and quiescence exit. We also examined the effect of voluntary exercise on RGL proliferation and expression of Ascl1 and found that it did not significantly increase proliferation of hippocampal RGLs, but only that of IPCs, as previously reported (Klempin et al., 2013). Together, our results demonstrate that neurogenic stimuli rapidly induce Ascl1 expression in quiescent RGLs, which in turn suggests that this factor might be implicated in RGL activation.

Ascl1 Is Absolutely Required for the Exit of RGLs from Quiescence

To directly address the role of *Ascl1* in the activation of DG RGLs, we generated triple-transgenic mice that were homozygous for a conditional mutant allele of *Ascl1* (Pacary et al., 2011) and also carried the *Glast-CreERT2* allele to delete *Ascl1* in RGLs in a tamoxifen-dependent manner (Mori et al., 2006) and the *Rosa26-floxed stop-YFP* reporter transgene to identify cells having undergone Cre-mediated recombination by their expression of YFP (Srinivas et al., 2001). Administration of tamoxifen for 5 days to postnatal day 60 (P60) triple-transgenic mice and control mice (carrying *Glast-CreERT2* and *Rosa26-floxed stop-YFP*, but WT for *Ascl1*) resulted in widespread induction of YFP in radial GFAP$^+$, Nestin$^+$ RGLs (Figures 3A, 3B, and data not shown). However, examination by immunolabeling revealed that a fraction of SGZ cells that expressed YFP and had therefore recombined the *Rosa26-floxed stop-YFP* locus also expressed Ascl1 and had therefore not recombined the *Ascl1^{flox}* locus, indicating that recombination at the two loci was partially uncoupled (Vooijs et al., 2001). We also examined triple-transgenic mice carrying a different conditional mutant allele of *Ascl1*, in which a *PGK promoter-neo* cassette remained inserted on the 3′ side of the *Ascl1* locus (*Ascl1^{neoflox}* mice; Figures 3C and S3A). Interestingly, even without tamoxifen-induced recombination, *Ascl1* RNA and protein expressions were significantly reduced in the DG of these mice compared with WT mice (Figures 3D and S3B), suggesting that *Ascl1^{neoflox}* is a hypomorphic allele (Nagy et al., 1998). Analysis of *Ascl1^{neoflox}* mice at P10 did not reveal any overt morphological defect of the DG, and the rate of RGL proliferation was similar to that found in WT mice, indicating that the hypomorphic allele of *Ascl1* does not result in a developmental defect in the DG (Figure S3). Tamoxifen administration to these mice at P60–P64 resulted in undetectable *Ascl1* expression in the DG at P90 (*Ascl1^{neo}cKO* mice; Figures 3C, 3D, S3A, and S3B). We therefore used *Ascl1^{neo}cKO* mice in the rest of this study to examine the effect of loss of *Ascl1* on hippocampal neurogenesis, and we used *Ascl1^{neoflox}* mice to examine the effect of a reduced expression of *Ascl1*.

To determine whether *Ascl1* deletion has an impact on DG RGLs, we administered tamoxifen at P60–P64 and examined *Ascl1^{neo}cKO* mice 1 month later, at P90. Double labeling for YFP and for MCM2, Ki67, or bromodeoxyuridine (BrdU) after a 2 hr pulse revealed a near-complete absence of proliferating YFP$^+$ cells in the SGZ of *Ascl1^{neo}cKO* mice (*Ascl1^{neo}cKO* mice versus WT mice, 19.65 ± 19.65 versus 4,635 ± 921 YFP$^+$ MCM2$^+$ cells; 0 versus 2,875 ± 815 YFP$^+$ KI67$^+$ cells; 9.8 ± 4.9 versus 471.6 ± 115.5 YFP$^+$ BrdU$^+$ cells), while proliferating cells were present in *Ascl1^{neoflox}* mice, albeit in reduced numbers (2,233 ± 632 MCM2$^+$ cells; 1,225 ± 298 KI67$^+$ cells; 142.8 ± 10.2 BrdU$^+$ cells; Figures 3E–3H). Moreover, triple labeling for YFP, GFAP, and MCM2 to mark activated stem cells or Ki67 to mark proliferating stem cells demonstrated a complete absence of recombined RGLs that were activated or proliferating in *Ascl1^{neo}cKO* mice, while activated and cycling RGLs were present, but less numerous, in *Ascl1^{neoflox}* mice than in WT mice (*Ascl1^{neo}cKO* versus *Ascl1^{neoflox}* versus WT mice, 0 versus 126.3 ± 26.3 versus 281.2 ± 70.1 MCM2$^+$ cells; 0 versus 66.0 ± 29.5 versus 161.2 ± 48.5 KI67$^+$ cells; Figures 3I–3K; see also Figures S3D–S3G for measures of proliferation in the DG of *Ascl1^{flox}* and *Ascl1cKO* mice). Rarely dividing RGLs, characterized by their capacity to retain BrdU, were labeled in tamoxifen-injected P90 mice by 10 days of BrdU administration followed by 20 days of chase (Figure 3L). BrdU label-retaining cells were present in WT mice and to a lesser extent in *Ascl1^{neoflox}* mice, but were again completely absent in *Ascl1^{neo}cKO* mice (0 cells in *Ascl1^{neo}cKO* mice; 9.7 ± 4.9 cells in *Ascl1^{neoflox}* mice; 43.8 ± 6.2 cells in WT mice; Figure 3L). We could thus demonstrate by several independent methods the complete inability of RGLs to exit quiescence and divide in the absence of *Ascl1*, and therefore establish that *Ascl1* is essential for activation of RGLs in the adult hippocampus. Interestingly, *Ascl1* deletion had no significant effect on the rate of RGL proliferation in the postnatal DG. When *Ascl1* was deleted by tamoxifen administration in *Ascl1^{neo}cKO* mice at P7, RGLs continued to proliferate at P10 at a rate that was not significantly different from that seen in WT mice (Figure S3H). Therefore, the absolute requirement of *Ascl1* for RGL activity is specific to the adult DG.

Dividing RGLs in the adult hippocampus generate IPCs that proliferate before producing postmitotic granule neurons (Bonaguidi et al., 2011; Kempermann et al., 2004). Since RGLs require *Ascl1* to divide, the production of IPCs and their neuronal progeny might also depend on *Ascl1* function. The absence of Ki67$^+$, MCM2$^+$, and BrdU$^+$ cells in the SGZ of *Ascl1^{neo}cKO* mice (Figures 3F–3H) already suggested that IPCs are indeed missing in these mice. We further examined neurogenesis by double labeling the hippocampus of *Ascl1^{neo}cKO* mice for YFP and for Tbr2 to mark IPCs, for DCX to mark neuroblasts, and for NeuN to mark granule neurons (Kempermann et al., 2004; Ming and Song, 2011). No YFP$^+$ cells expressed these markers in the DG of *Ascl1^{neo}cKO* mice at P90, demonstrating that no new IPCs or granule neurons were produced in these mice (Figures S3I–S3K; data not shown). *Ascl1* is therefore absolutely required for the generation of IPCs and for neurogenesis in the hippocampus.

Ascl1 Is Required for RGL Activation in the V-SVZ

We also examined neurogenesis in the V-SVZ of *Ascl1^{neo}cKO* mice to determine whether the role of *Ascl1* in adult RGLs extends to the other main neurogenic region of the adult rodent brain. Analyzing the expression of GFAP, DCX, EGFR, and GFP in *Ascl1^{neo}cKO* and WT mice showed that deletion of

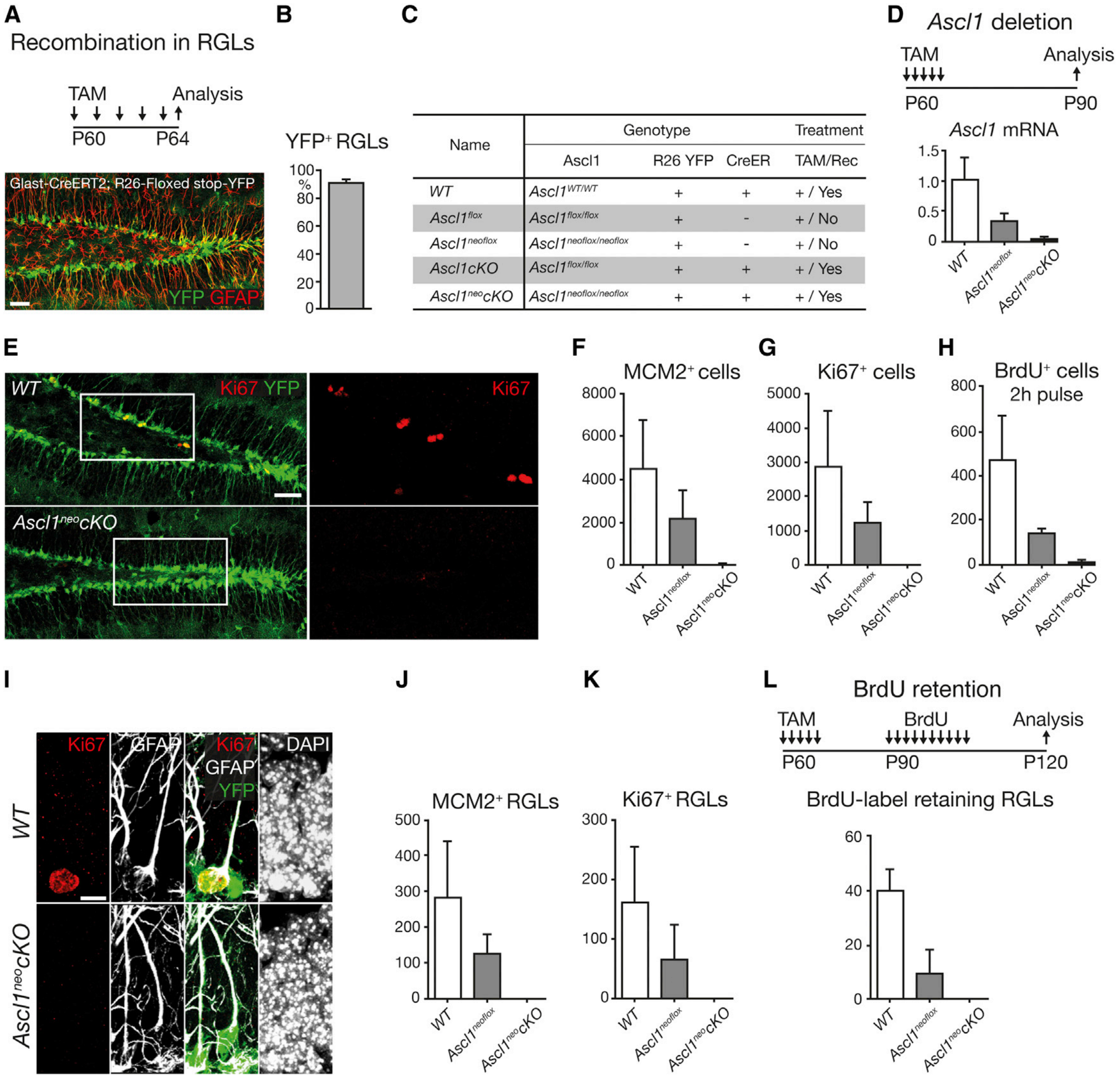

Figure 3. Block of Activation and Proliferation of Hippocampal Stem Cells by Conditional Inactivation of *Ascl1*

(A and B) Administration of tamoxifen at P60–P64 in *Glast-CreERT2; Rosa26-floxed stop-YFP* mice followed by the analysis of YFP expression at P64 (A) and the percentage of RGLs that express YFP (B) show the high efficiency of recombination of the YFP reporter allele. n = 3.

(C) Presentation of the five mouse lines analyzed in this study, including (first column) their names, (second column) the *Ascl1* allele they carry (WT, *flox*, or *neoflox*), (third column) the *Rosa26-floxed stop-YFP* reporter transgene they all carry (*R26 YFP*), (fourth column) whether they carry (+) or do not carry (–) the deleter allele *Glast-CreERT2* (*CreER*), and (fifth column) whether the tamoxifen injection they have all received (TAM, +) results in inactivation of *Ascl1* and the YFP reporter (Rec, Yes) or not (Rec, No).

(D) Conditional inactivation of the *Ascl1* gene by tamoxifen administration at P60–P64 and analysis at P90 of *Ascl1* transcripts by quantitative RT-PCR on laser-capture-microdissected SGZ tissue shows that *Ascl1* expression is strongly reduced in *Ascl1*^{neoflox} mice compared to WT mice and is eliminated in *Ascl1*^{neo}*cKO* mice. The graph shows expression levels normalized to *Gapdh* and relative to *Ascl1* expression in WT. n = 3 in each genotype.

(E–H) Labeling for the proliferation marker Ki67 and for YFP to mark recombined cells (right panels are enlargements of the areas boxed in left panels) (E) and the total numbers per DG of MCM2$^+$ cells (F), Ki67$^+$ cells (G), and BrdU$^+$ cells 2 hr after BrdU administration (H) show that cells do not proliferate in the DG of P90 *Ascl1*^{neo}*cKO* mice, and that the lower level of *Ascl1* expression in *Ascl1*^{neoflox} mice results in reduced proliferation compared to WT mice. p values in WT versus *Ascl1*^{neo}*cKO*, MCM2$^+$ cells = 0.058; Ki67$^+$ cells = 0.031; BrdU$^+$ cells = 0.0162. n = 5 for WT, n = 4 for *Ascl1*^{neo}*cKO* (F), 4 (G), and 3 (H).

(I–L) Labeling for Ki67 and GFAP (I) and total numbers per DG of MCM2$^+$ and Ki67$^+$ RGLs (J and K) and BrdU$^+$ cells following prolonged administration and chase of BrdU (L) demonstrate the absence of activated, proliferating, and BrdU label-retaining RGLs in *Ascl1*^{neo}*cKO* mice and their reduced numbers in *Ascl1*^{neoflox} mice. p values in WT versus *Ascl1*^{neo}*cKO*, MCM2$^+$ cells = 0.016; Ki67$^+$ cells = 0.038; BrdU$^+$ cells = 0.0084. n = 5 for WT, n = 4 for *Ascl1*^{neo}*cKO* (J), 4 (K), 5 (L, WT), 3 (L, *Ascl1*^{neoflox}), and 3 (L, *Ascl1*^{neo}*cKO*). Scale bars, 40 μm in (A) and (E) and 10 μm in (I). Values represent mean values, and error bars represent SDs.

Ascl1 results in a severe decrease in the fraction of RGLs of the V-SVZ that are activated (GFAP$^+$ EGFR$^+$) and proliferate (BrdU label retaining), and in a severe reduction in the production of DCX$^+$ neuroblasts (Figures 4 and S4). Therefore, *Ascl1* is essential for NSC activation and proliferation and for neurogenesis in the two main neurogenic regions of the adult brain.

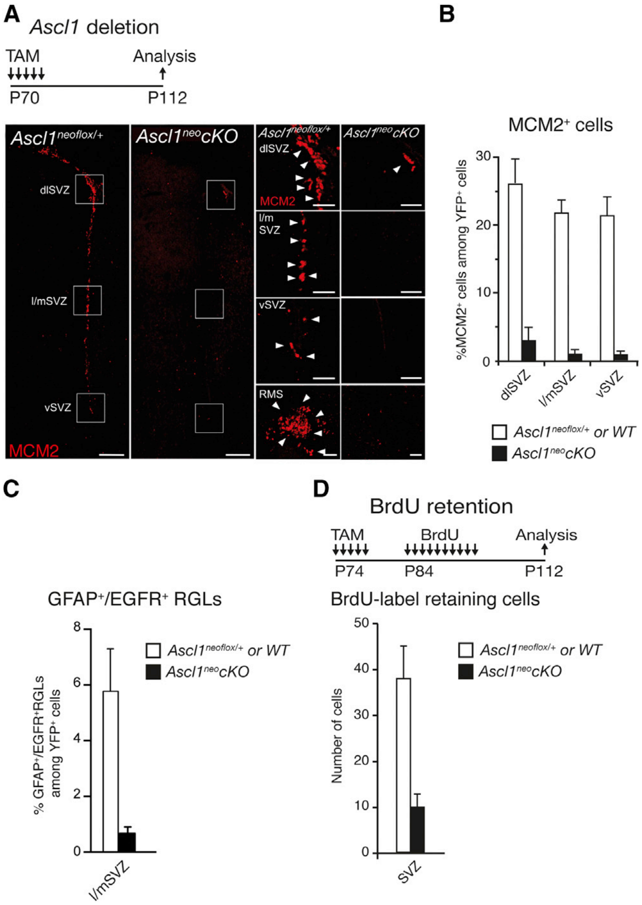

Figure 4. Block of Activation and Proliferation of V-SVZ Stem Cells by Conditional Inactivation of *Ascl1*

(A–D) Labeling for the activation marker MCM2 (right panels are enlargements of the areas boxed in left panels; arrowheads point to MCM2$^+$ cells) (A) and numbers of MCM2$^+$ cells (B), activated RGLs identified by coexpression of GFAP and EGFR (C) among recombined YFP+ cells in the dorsal (dlSVZ), medial, lateral (l/mSVZ), and ventral (vSVZ) V-SVZ, and BrdU label-retaining cells in the whole SVZ following prolonged administration and chase of BrdU (D) show that RGLs are not activated and do not proliferate in the V-SVZ of P112 *Ascl1*^{neo}*cKO* mice. p values in WT versus *Ascl1*^{neo}*cKO*, MCM2$^+$ cells = 0.00014 in dlSVZ, < 0.0001 in l/mSVZ, < 0.0001 in vSVZ; n = 5; GFAP$^+$, EGFR$^+$ cells = 0.00033; n = 4; BrdU label-retaining cells = 0.0056; n = 4. Scale bars, 200 μm in whole panels and 50 μm in enlarged panels. Values represent mean values, and error bars represent SDs.

Ascl1 Acts Cell Autonomously to Promote RGL Proliferation

Ascl1 is expressed in proliferating RGLs, and the loss of *Ascl1* results in an arrest of RGL proliferation, suggesting that this gene is required in RGLs to promote their divisions. However, Ascl1 is also expressed in some IPCs, raising the alternative possibility that *Ascl1* is primarily required for the generation and/or division of IPCs, and that the arrest of RGL divisions is a secondary consequence of the loss of IPCs. In particular, a loss of IPCs might disrupt Notch signaling in the SGZ, resulting in a transient increase in RGL proliferation, followed later by a reduction of proliferation due to RGL exhaustion (Ables et al., 2010; Ehm et al., 2010; Lavado et al., 2010). To address this possibility, RGL proliferation was monitored in *Ascl1*^{neo}*cKO* mice just 4 days after the beginning of tamoxifen administration at P60. RGLs had already stopped dividing in P64 *Ascl1*^{neo}*cKO* mice, indicating that the effect of *Ascl1* deletion on RGL proliferation is rapid and therefore likely direct (Figures 5A and 5B). Moreover, although RGLs in the DG of P64 *Ascl1*^{neo}*cKO* mice are mostly YFP$^+$ and have therefore recombined (Figures 3B and 5A), WT Tbr2$^+$ IPCs and DCX$^+$ neuroblasts that were produced by RGLs before tamoxifen administration at P60–P64 are still present (Figure 5C). This suggests that the RGL proliferation defect is not the consequence of a loss of *Ascl1*-mutant IPCs and neuroblasts, i.e., *Ascl1* is required cell autonomously for RGL divisions.

To more rigorously address the cell autonomy or noncell autonomy of *Ascl1* function, we performed a mosaic analysis. We activated CreERT2 in a fraction of RGLs with only one injection of tamoxifen at P60, resulting 1 month later in an intermingling of recombined YFP-positive RGLs and nonrecombined YFP-negative RGLs and their progenies (Figure 5D). In WT mice that had received a single tamoxifen injection, a fraction of recombined YFP$^+$ RGLs was proliferating (Figures 5E and 5F). In contrast, recombined YFP$^+$ RGLs did not proliferate in mosaic *Ascl1*^{neo}*cKO* and *Ascl1cKO* mice, irrespective of the recombination efficiency (Figures 5E, 5F, and S5). Therefore, the proliferation defect of *Ascl1*-mutant RGLs cannot be rescued by the presence of nearby WT cells, demonstrating that *Ascl1* is required cell autonomously in RGLs for their divisions.

Ascl1-Deficient RGLs Do Not Respond to Mitogenic Stimuli

Next, we examined the phenotype of the cells remaining in the SGZ after *Ascl1* deletion. Antibody labeling of the DG of P90 *Ascl1*^{neo}*cKO* mice revealed that these cells retain the typical radial morphology of RGLs and maintain expression of the RGL markers GFAP, Nestin, and Sox2 and do not express the astrocytic marker S100β or the oligodendrocyte progenitor marker Olig2 (Figures 6A, 6B, and S6A–S6C). RT-PCR analysis showed that expression of *p16*^{INK4a}*/Cdkn2a* was not elevated in the DG of *Ascl1*^{neo}*cKO* mice, suggesting that RGLs in these mice do not become senescent (Molofsky et al., 2006). Moreover, RGLs in *Ascl1*^{neo}*cKO* maintained normal levels of the cyclin-dependent kinase inhibitor p57^{Kip2}, which is required for quiescence of hippocampal RGLs (Furutachi et al., 2013). These data, together with the lack of MCM2 expression in *Ascl1*-deficient RGLs (Figure 3J), show that *Ascl1* is not required for the maintenance of RGLs, but specifically for their activation, and that loss of *Ascl1* keeps RGLs in an inactive and undifferentiated state.

Although *Ascl1*-deficient RGLs are unable to proliferate when in a steady state, they might still be able to respond to a potent neurogenic stimulus such as KA. We therefore injected tamoxifen in *Ascl1*^{neo}*cKO* mice at P60 to delete *Ascl1*, then injected KA at P86 and analyzed the DG at P90. KA failed to activate RGLs in *Ascl1*^{neo}*cKO* mice, while it strongly activated them in WT mice (Figures 6E and 6F). Therefore, loss of *Ascl1* in RGLs results in an inactive state that cannot be reversed by KA stimulation. We also confirmed the inactivated state of

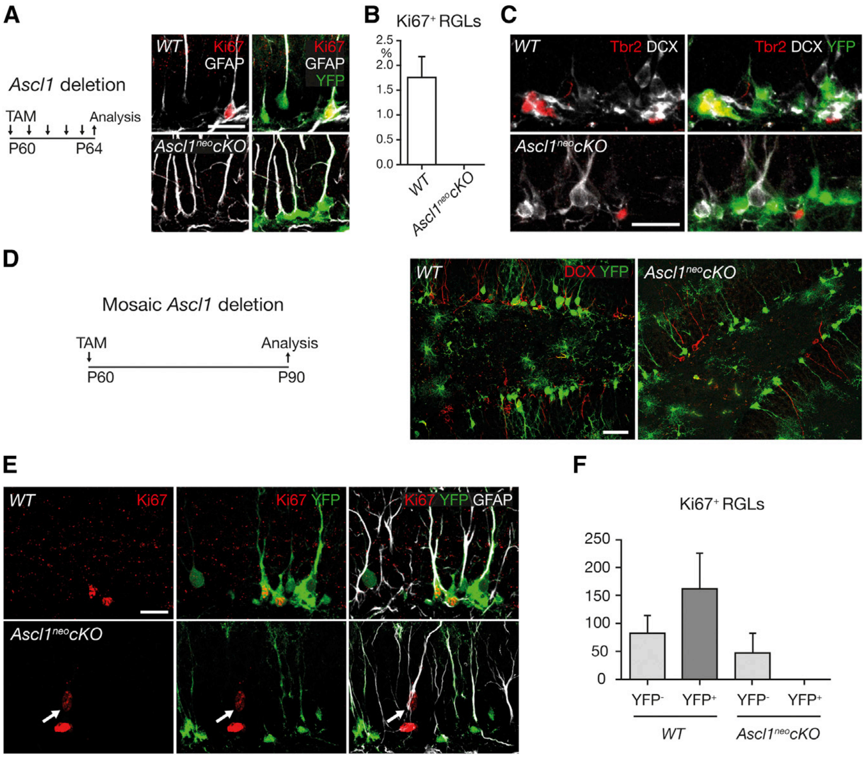

Figure 5. Cell-Autonomous Function of *Ascl1* in Hippocampal Stem Cell Proliferation

(A–C) RGLs have ceased to proliferate in the *Ascl1*^{neo}*cKO* DG as early as 4 days after the first tamoxifen injection (A and B). Percentages of RGLs expressing Ki67 in each genotype (B). Nonrecombined (YFP$^-$) Tbr2$^+$ IPCs and DCX$^+$ neuroblasts remain in the DG of the *Ascl1*^{neo}*cKO* mouse at P64 (C), but they cannot rescue the proliferation defect of recombined (YFP$^+$) RGLs (B). p value, Ki67$^+$ RGLs in WT versus *Ascl1*^{neo}*cKO* = 0.0019. n = 3 in each genotype.

(D–F) A single injection of tamoxifen results in recombination in only a fraction of RGLs, and nonrecombined RGLs produce YFP$^-$ DCX$^+$ neuroblasts in both WT and *Ascl1*^{neo}*cKO* mice (D). In the *Ascl1*^{neo}*cKO* DG, nonrecombined (YFP$^-$, arrow) RGLs proliferate, but recombined (YFP$^+$) RGLs do not, demonstrating that the presence of WT RGLs and their progeny does not rescue the proliferation defect of *Ascl1*-deficient RGLs (E and F). Total numbers of Ki67$^+$ RGLs among YFP$^+$ and YFP$^-$ RGLs in the two genotypes (F). p value, Ki67$^+$ YFP$^+$ RGLs in WT versus *Ascl1*^{neo}*cKO* = 0.0003. n = 3 (WT) and 6 (*Ascl1*^{neo}*cKO*). Scale bars, 20 μm in (A), (C), and (E) and 40 μm in (D). Values represent mean values, and error bars represent SDs.

Ascl1-deficient RGLs by performing an in vitro neurosphere assay in the presence of mitogens (Ehm et al., 2010). The DG was dissected from 7- to 8-week-old mice and dissociated, and single-cell suspensions were cultured at clonal density in the presence of FGF2 and EGF. Cultures of WT DG cells produced large numbers of primary neurospheres that generated secondary neurospheres when passaged. In contrast, *Ascl1*^{neo}*cKO* DG cell cultures produced very few neurospheres, and antibody labeling showed that these neurospheres maintained expression of Ascl1 and therefore originated from cells that had escaped recombination (Figures 6G, 6H, and S6E). Therefore, *Ascl1*-deficient RGLs are quiescent and unable to respond to mitogens and divide in vitro.

We next examined whether the inactive state of *Ascl1*-deficient RGLs was stable over a longer period. Analysis of *Ascl1*^{neo}*cKO* mice 5 months after *Ascl1* deletion, at P210, showed that RGLs retained a radial morphology and GFAP expression and remained Ki67 negative (Figure 6I). Interestingly, while the total number of RGLs in the DG of WT mice decreased considerably between P65 and P90 and decreased further at P210 (22,116 ± 1,681 at P65, 14,453 ± 1,021 at P90, and 12,516 ± 1,063 at P210), their number in *Ascl1*^{neo}*cKO* mice did not change between P65 and P90 and decreased only slightly and nonsignificantly at P210 (24,533 ± 1,399 at P65, 26,098 ± 1,913 at P90, and 20,764 ± 395 at P210; Figure 6J). As the age-related attrition of hippocampal RGLs is thought to result from non-self-renewing divisions (Bonaguidi et al., 2012; Encinas et al., 2011), the maintenance of RGL numbers in older *Ascl1*^{neo}*cKO* mice supports the finding that RGLs do not divide in these mice.

Ascl1 Directly Regulates Cell-Cycle Genes in Hippocampal Stem Cells

To identify target genes that mediate the proliferative role of *Ascl1* in hippocampal stem cells, we examined the genome-wide binding of Ascl1 in adult hippocampus-derived NSCs (Knobloch et al., 2013) by chromatin immunoprecipitation-sequencing (ChIP-seq) with an anti-serum against Ascl1 (Figures 7 and S7). Ascl1 was bound to 7,826 high-confidence sites in the genome of AH-NSCs (Figure 7A), a majority of which mapped to enhancers previously identified in cultured NSCs (Figure 7B; Martynoga et al., 2013). Moreover, a large fraction of enhancers active in proliferating NSCs featured an Ascl1-binding event in AH-NSCs (Figure 7C).

We then established a list of the genes associated with an Ascl1-binding event in the AH-NSC genome (Table S1) and searched for candidate direct targets of Ascl1, i.e., genes that are both bound by Ascl1 and misregulated in RGLs of *Ascl1*^{neo}*cKO*. We reasoned that since *Ascl1* is required for NSC proliferation in both DG and V-SVZ, it might regulate some of the same genes in the two tissues. We therefore intersected our list of Ascl1-bound genes with a recently published list of V-SVZ genes enriched in activated NSCs compared with quiescent NSCs (Figure S7; Table S2) (Codega et al., 2014). The 250 genes that are both bound by Ascl1 in AH-NSCs and enriched in activated V-SVZ NSCs include, in addition to known *Ascl1* targets such as the Notch ligands *Dll1* and *Dll3* (Castro et al., 2011), several cell-cycle regulators such as *E2f1*, *Ccnd2*, *Cdc6*, and *Skp2*. To establish whether these genes are regulated by *Ascl1* in DG RGLs, we used fluorescence-activated cell

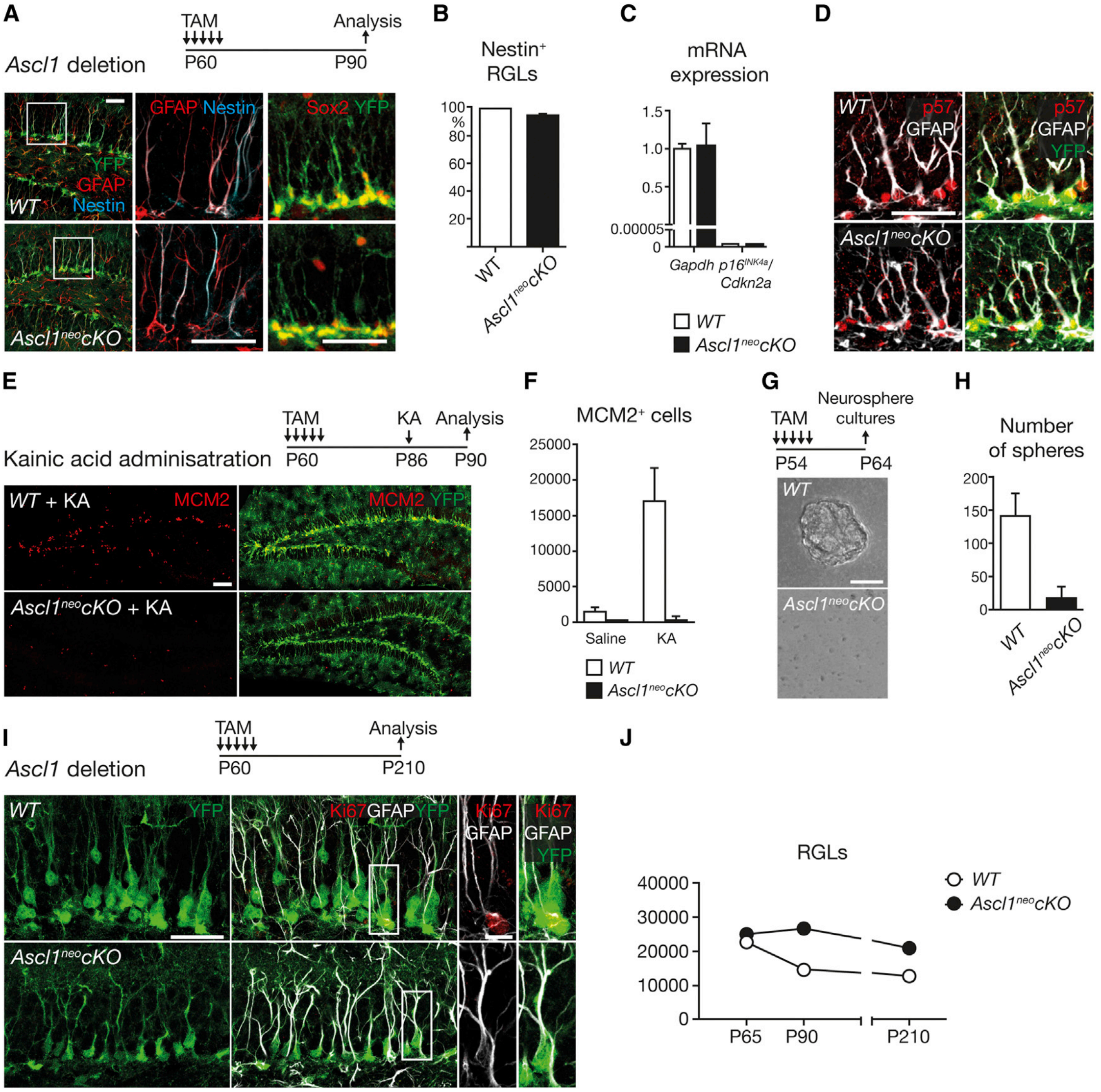

Figure 6. Unresponsiveness of *Ascl1*-Deficient Hippocampal Stem Cells to Extrinsic Stimuli

(A and B) *Ascl1*-deficient RGLs in P90 *Ascl1*^{neo}*cKO* mice retain a neural stem cell phenotype characterized by expression of the neural stem/progenitor cell markers Nestin, Sox2, and GFAP and a radial morphology. We note that Nestin remains expressed in *Ascl1*-mutant quiescent hippocampal RGLs, albeit at a lower level than in WT RGLs (not shown), whereas it is not expressed in quiescent RGLs in the V-SVZ (Codega et al., 2014). n = 3 in each genotype.

(C) Quantitative RT-PCR analysis of the cyclin-dependent kinase inhibitor *p16*^{INK4a}/*Cdkn2a* in the laser-capture-microdissected SGZ shows that expression of this marker of RGL senescence is barely detectable and not increased in the *Ascl1*^{neo}*cKO* SGZ compared with WT mice. Expression levels normalized to *Gapdh*. n = 3 in each genotype.

(D) Expression of the cyclin-dependent kinase inhibitor p57^{Kip2}, a marker of quiescent RGLs, is similar in the DG of WT and *Ascl1*^{neo}*cKO* mice.

(E and F) KA administration stimulates cell proliferation in the SGZ of WT mice, but not in the SGZ of *Ascl1*^{neo}*cKO* mice. Total numbers of MCM2^{+} cells in the SGZ in each condition (F). p value, MCM2^{+} cells in WT versus *Ascl1*^{neo}*cKO* = 0.0183. n = 3 in each condition.

(G and H) Clonal cultures of dissociated DG cells produced a large number of primary neurospheres from WT DG and only few neurospheres from *Ascl1*^{neo}*cKO* DG. Total numbers of spheres obtained from clonal cultures of one dissociated DG. p value = 0.0060. n = 3 in each genotype. Further analysis showed that the few neurospheres from *Ascl1*^{neo}*cKO* DG have escaped recombination and express Ascl1 (see Figure S6E).

(I) At 5 months after *Ascl1* inactivation, SGZ cells in *Ascl1*^{neo}*cKO* mice retain morphological and molecular features of RGLs and remain nonproliferative. The boxed areas are enlarged on the right and show a proliferating RGL in WT and a nonproliferating RGL in *Ascl1*^{neo}*cKO*. Note the accumulation of YFP^{+} granule neurons above the SGZ in WT, and not in *Ascl1*^{neo}*cKO*, mice.

(J) RGL numbers decrease significantly with age in WT mice, while RGL numbers are not significantly different in *Ascl1*^{neo}*cKO* mice at P65, P90, and P210. Total numbers of RGLs per DG. p values, WT RGLs at P65 versus P90 = 0.0176; at P90 versus P210 = 0.3655; *Ascl1*^{neo}*cKO* RGLs at P65 versus P90 = 0.5453; at P90 versus P210 = 0.0525. n = 3 in each condition. Scale bars, 40 μm in (A), (D), (E), (G), and (I) and 10 μm in enlargement of (I). Values represent mean values, and error bars represent SDs.

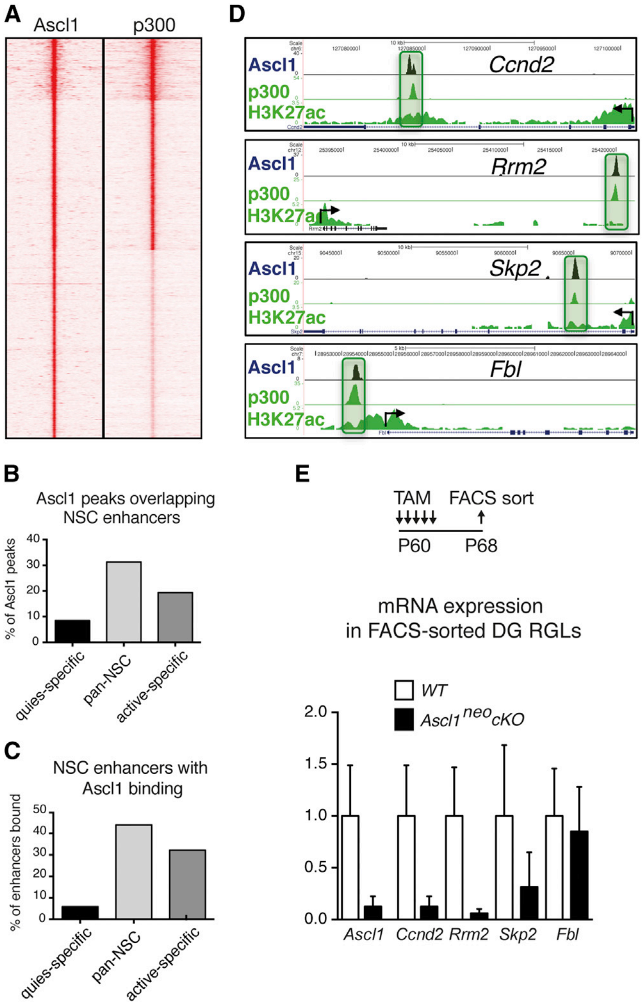

Figure 7. Direct Targets of Ascl1 in Adult Hippocampal Stem Cells

(A–C) Heat map representation of Ascl1-binding signals in adult hippocampus-derived neural stem cells (AH-NSCs) alongside p300-binding signals in proliferating NSCs at the same genomic locations to mark enhancers (from Martynoga et al. [2013]) (A), and the distribution of Ascl1-binding sites in different classes of NSC enhancers (from Martynoga et al. [2013]) (B) shows that a large fraction of Ascl1-binding sites are located in enhancers present in proliferating NSCs, while reciprocally a large fraction of enhancers present in proliferating NSCs are bound by Ascl1 (C). quies-specific, enhancers specific for quiescent NSCs; pan-NSC, enhancers present in both quiescent and activated NSCs; active-specific, enhancers specific for activated NSCs (from Martynoga et al. [2013]).

(D) ChIP-seq signals in AH-NSCs for Ascl1 and the enhancer marks p300 and H3K27ac (from Martynoga et al. [2013]) at five genes coding for components of the cell-cycle machinery. Significant binding peaks are indicated by green rectangles. Ascl1 binds enhancers in the four genes in AH-NSCs.

(E) Quantitative RT-PCR analysis of fluorescent-activated cell-sorted YFP+ cells from the DG of WT and *Ascl1*^{neo}*cKO* mice shows that expression of *Ascl1* and two Ascl1-bound genes, the cell-cycle genes *Ccnd2* and *Rrm2*, are strongly reduced in *Ascl1*^{neo}*cKO* cells, while the expression of *Skp2* and *Fbl*, two other Ascl1-bound genes, is unchanged. The graph shows expression levels normalized to *Gapdh* and *Ppia* and relative to gene expression in WT. The remaining *Skp2* expression in *Ascl1*-mutant cells might be due to the presence of Glast$^+$ parenchymal astrocytes in the sorted population. p values in WT versus *Ascl1*^{neo}*cKO*, *Ascl1* = 0.0016; *Ccnd2* = 0.0016; *Rrm2* = 0.0011; *Skp2* = 0.052; *Fbl* = 0.65. n = 4 in each genotype. Values represent mean values, and error bars represent SDs.

sorting to purify YFP$^+$ cells from the DG of *Ascl1*^{neo}*cKO* and WT mice 4 days after tamoxifen treatment, and we analyzed gene expression by qRT-PCR. We found that the cyclin *Ccnd2* and the ribonucleotide reductase subunit *Rrm2* were significantly downregulated when *Ascl1* was deleted in RGLs, while *E2f1* and *Cdc6* expression were not detectable in either WT or mutant cells, and expression of *Skp2* and non-cell-cycle genes such as *Fbl* were not significantly reduced (Figure 7E). Together, these data demonstrate that *Ascl1* controls the proliferation of hippocampal RGLs by directly activating the expression of *Ccnd2*, *Rrm2*, and possibly additional cell-cycle genes.

DISCUSSION

Stem cells in adult tissues respond to environmental signals by adjusting the production of mature cells to the needs of the tissue. Deciphering the pathways that link physiological stimuli to NSC activity requires characterization of the intrinsic machinery that controls stem cell activation. We show in this study that signals regulating hippocampal stem cell activity control the expression of Ascl1, and that this factor has an essential role in stem cell activation.

Neurogenic and Antineurogenic Signals Converge on Ascl1 Expression

Ascl1 expression in the adult brain has often been described as being restricted to IPCs in the SVZ and DG (Lugert et al., 2012; Parras et al., 2004). We found, however, that Ascl1 is already expressed in proliferating RGLs in the hippocampus, in agreement with earlier studies (Breunig et al., 2007; Kim et al., 2011). Ascl1 expression is then presumably maintained by the IPCs that are produced when RGLs divide. Most Ascl1$^+$ RGLs are activated, but only about a third of activated RGLs express Ascl1 detectably, which might be due to an oscillation of Ascl1 expression in activated RGLs as in embryonic neural progenitor cells (Imayoshi et al., 2013).

The results of our experiments with KA-injected mice suggest that neurogenic stimuli such as neuronal activity promote stem cell activation in the hippocampus by inducing Ascl1 expression in quiescent RGLs. The essential role of Ascl1 in the activation of adult NSCs suggests that other neurogenic signals controlling this step, including Wnt signals in the hippocampus (Jang et al., 2013; Qu et al., 2010, 2013; Seib et al., 2013) and VEGF in the V-SVZ (Calvo et al., 2011), might also act by inducing Ascl1 expression. Our results with *RBPJk* conditional mutant mice also suggest that antineurogenic stimuli, including the Notch pathway, suppress stem cell activity by repressing *Ascl1* expression in RGLs. The mechanism by which the Notch-RBPJk pathway represses *Ascl1* expression can be inferred from studies in the embryonic brain and in cancer cells, which have shown that the Notch-induced Hes factors directly repress the *Ascl1* gene (Kageyama et al., 2005). Moreover, antineurogenic signals may also target Ascl1 protein activity. The maintenance of hippocampal stem cell quiescence by BMPs (Mira et al., 2010) may involve an inactivation of Ascl1 protein by BMP effectors Id proteins, which are known to block Ascl1 activity by preventing its dimerization with E proteins (Nakashima et al., 2001). FoxO3, which acts downstream of the insulin/IGF-1

signaling pathway to maintain hippocampal stem cell quiescence, shares many targets with Ascl1 and may also function by antagonizing Ascl1 function (Webb et al., 2013). Inactivation of Ascl1 protein in RGLs that have begun to transcribe the *Ascl1* gene may be important to slow down the transition to an active state or to accelerate the return of active RGLs to quiescence.

Mice carrying the hypomorphic allele *Ascl1*^{neoflox} express *Ascl1* in the DG at a reduced level and retain only a small fraction of the proliferating RGLs found in WT mice (Figure 3). The finding that a change in *Ascl1* expression level translates into a change in the fraction of RGLs that proliferate suggests that extrinsic signals may fine-tune the rate of hippocampal neurogenesis by modulating the expression level of Ascl1, as shown for PDK1/Akt signaling, which regulates Ascl1 protein stability in the embryonic brain (Oishi et al., 2009).

Ascl1 Has a Crucial Role in Hippocampal Stem Cell Activation

A cell that exits quiescence and transits from the G_0 to the early G_1 phase of the cell cycle assembles a prereplication complex that contains minichromosome maintenance proteins, including MCM2. MCM2 expression therefore marks not only cycling cells, but also activated cells that have exited the quiescent state but not yet re-entered the cell cycle (Niu et al., 2011; Stoeber et al., 2001). The glutamate receptor agonist KA induces the expression of Ascl1 before that of MCM2, indicating that Ascl1 induction is one of the first steps in the pathway through which neuronal activity promotes the activation of quiescent RGLs. Since MCM2 is not expressed in *Ascl1*-deficient RGLs even after KA stimulation, *Ascl1* is also absolutely required for the quiescence exit of hippocampal RGLs.

Because *Ascl1*-deficient RGLs do not exit the quiescent state, it is not possible to ascertain whether *Ascl1* also regulates the cell-cycle progression of RGLs. However, *Ascl1* promotes the proliferation of progenitor cells in the embryonic brain (which do not enter quiescence), and it directly regulates the expression of cell-cycle regulatory genes in AH-NSCs, including *Ccnd2* and *Rrm2*, suggesting that it promotes the divisions of hippocampal RGLs in addition to their activation. *Ascl1* has previously been shown to promote cell proliferation in cancer cells and in the injured zebrafish retina by regulating Wnt-signaling genes (Osada et al., 2008; Ramachandran et al., 2011; Rheinbay et al., 2013). In glioblastoma cancer stem cells (GBM CSCs), Ascl1 has been shown to bind a site near the Wnt antagonist gene *Dkk1*, whose regulation mediates *Ascl1* activity in these cells (Rheinbay et al., 2013). In AH-NSCs, however, Ascl1 does not bind this site and many other sites bound in GBM CSCs, and reciprocally, many Ascl1-bound loci were found in AH-NSCs, and not in GBM CSCs, including sites near the cell-cycle regulators *E2f1*, *Ccna1*, *Ccnd2*, and *Skp2* (data not shown), suggesting that *Ascl1* controls the proliferation of hippocampal RGLs by regulating different genes from those regulated in cancer cells and the injured retina.

Transcriptomic and genetic studies suggest that NSCs undergo profound changes in their oxygen and lipid metabolisms and cell adhesion properties when they exit quiescence (Knobloch et al., 2013; Martynoga et al., 2013; Renault et al., 2009). Further characterization of *Ascl1* target genes in RGLs should elucidate whether *Ascl1* directly controls these physiological changes in addition to promoting cell-cycle re-entry and cell-cycle progression. *Ascl1* inactivation also blocks RGL activity in the V-SVZ. Whether Ascl1 acts through the same downstream mechanisms in the two adult neurogenic regions remains to be addressed.

In contrast with the complete lack of RGL activity in the adult DG in the absence of *Ascl1*, RGL proliferation was not significantly affected by *Ascl1* deletion in the early postnatal DG, supporting our earlier finding that Ascl1 is not required for DG morphogenesis during embryonic development (Galichet et al., 2008). Interestingly, cell proliferation in the DG becomes also increasingly dependent on the *Ascl1* target *Ccnd2* between early postnatal and adult stages (Ansorg et al., 2012). Therefore, a switch in the genetic control of DG RGL proliferation occurs during the first few weeks of life, with the activation of a mitogenic pathway involving *Ascl1* and *Ccnd2*.

Ascl1-deficient RGLs remain in *Ascl1*^{neo}*cKO* mice for at least 5 months without dividing, differentiating, or dying. The permanent cell-cycle arrest can be readily explained by the cell-autonomous role of *Ascl1* in RGL activation. The lack of astrocyte differentiation may also be partially explained by the absence of proliferation, as astrogenesis is normally coupled to RGL divisions (Encinas et al., 2011). However, astrocytes can also be generated by direct differentiation of RGLs without cell division (Bonaguidi et al., 2011). In the embryonic brain, activation of Notch signaling by ligands presented by IPCs and young neurons switches neurogenic progenitors to an astrocytic fate (Namihira et al., 2009). In the hippocampal SGZ, *Ascl1* deficiency greatly reduces the expression of the Notch effectors *Hes1* and *Hes5* (Figure S6D), likely due to the elimination of Notch-ligand-presenting IPCs and young neurons. The resulting decrease in Notch activity may thus block the astrocytic differentiation of *Ascl1*-deficient RGLs.

Numerous transcription factors have been shown to stimulate the self-renewal of stem cells in tissues such as the blood and the skin (Akala and Clarke, 2006; Goldstein and Horsley, 2012). These factors often act by regulating multiple aspects of the biology of the stem cells; for example, by suppressing their differentiation, senescence, or apoptosis (Lieu and Reddy, 2009; Souroullas et al., 2009). Only a few factors, such as Gata3 in hematopoietic stem cells (Ku et al., 2012) and Runx1 in hair follicle stem cells (Osorio et al., 2008), have been proposed to primarily regulate adult stem cell divisions. In the brain, the orphan nuclear receptor Tlx promotes hippocampal NSC proliferation through induction of Wnt7a and repression of p21/WAF1 (Niu et al., 2011; Qu et al., 2010). However, how Tlx is regulated is currently not known. The function of Ascl1 of controlling adult stem cell activity in response to environmental signals is therefore so far unique in the adult brain. It is akin to that of MyoD in muscle satellite stem cells, which is expressed shortly after quiescent satellite cells have been activated, and which in turn induces the expression of the component of the prereplication complex Cdc6 (Zhang et al., 2010). Identifying the molecular pathways that control *Ascl1* expression at transcriptional and posttranscriptional levels will be important in order to learn how to manipulate hippocampal neurogenesis for therapeutic purposes.

EXPERIMENTAL PROCEDURES

Animals

Mice were housed, bred, and treated according to the guidelines approved by the Home Office under the Animal (Scientific Procedures) Act 1986. All experimental procedures involving mice have been approved by the Animal Welfare and Ethical Review Panel of the National Institute for Medical Research. *RBPJk*$^{loxp/loxp}$ animals were generated as previously described (Han et al., 2002) and bred to *Glast::CreERT2* BAC transgenic mice (Slezak et al., 2007). *Ascl1*$^{neoflox/neoflox}$ mice, in which exon 1 of the *Ascl1* gene is flanked by loxP sites (Pacary et al., 2011), were bred with *Glast-CreERT2* knockin mice (Mori et al., 2006) and with *Rosa26-floxed stop-YFP* reporter mice (Srinivas et al., 2001) to generate *Glast-CreERT2*; *Ascl1*^{neoflox}; *R26 YFP* mice, which are heterozygous for *Glast-CreERT2* and homozygous for *Ascl1*^{neoflox} and *R26 YFP*. Both *Glast::CreERT2* and *Glast-CreERT2* lines target both radial and horizontal astrocytes in the DG. In order to remove the *PGK promoter-neo* cassette from the *Ascl1* locus, *Ascl1*^{neoflox} animals were crossed with *act*β*-Flp* mice (The Jackson Laboratory).

Tamoxifen, BrdU, and KA Administration

For activation of the CreERT2 recombinase, P60 animals were administered intraperitoneally (i.p.) 4-hydroxytamoxifen (TAM) for 5 consecutive days. For mosaic experiments, P60 animals received a single TAM injection at the same concentration. All animals including WT and *Ascl1*^{neoflox} mice received TAM injections. To examine proliferating progenitors, mice received a single i.p. injection of BrdU 2 hr prior to tissue collection. To examine slowly dividing RGLs, mice received 5 daily BrdU injections followed by 5 consecutive days of BrdU-containing drinking water. Mice were sacrificed 20 days later. Male mice received KA as a single i.p. injection and were monitored for 90 min after KA injection. Animals that did not display rearing and falling were sacrificed 1, 2, or 4 days later and processed as described below.

Tissue Preparation and Immunofluorescence

Animals were transcardially perfused with saline followed by 4% paraformaldehyde (PFA). Brains were postfixed with 4% PFA for 2 hr at 4°C and sectioned coronally at 40 μm with a vibratome. The immunofluorescence procedure and the primary and secondary antibodies are described in Supplemental Information.

Microscopic Analysis and Quantification

Labeled cells were counted in every ninth 40 μm section through the entire rostrocaudal length of the DG (−0.82 mm to −4.24 mm from bregma). Counted cells were divided by the number of z planes counted to obtain the number of cells per 1 μm, and then multiplied by the total length of the DG. To count RGLs (GFAP$^+$ radial cells), cells were deemed radial if the cell body clearly associated with a DAPI-positive nucleus was located in the SGZ and had a single radial process extending through at least two-thirds of the granule layer. In all figures, the cell numbers counted in WT and *Ascl1*^{neo}*cKO* mice are numbers of YFP$^+$ marker$^+$ double-labeled cells, while the numbers counted in *Ascl1*^{neoflox} mice are for Ki67$^+$ cells only, since YFP is not expressed in these mice.

Laser-Capture Microdissection, FACS Sorting, RNA Isolation, and Quantitative Real-Time PCR

Coronal sections 14 μm long were cut from fresh-frozen brains in OCT with a cryostat and placed on slides. The SGZ of WT, *Ascl1*^{neoflox}, and *Ascl1*^{neo}*cKO* mice was excised by a PLAM laser-capture microdissection system (Zeiss) and collected in an adhesive cap. RNA from microdissected tissue was extracted and purified using Arcturus Pico Pure RNA Isolation Kit (Applied Biosystems) and reverse transcribed using the High-Capacity cDNA Reverse Transcription Kit (Applied Biosystems). Gene expression was detected using TaqMan Gene Expression Assays (Applied Biosystems). The protocol used for fluorescent-activated cell sorting is described in Supplemental Information.

Statistical Analyses

Statistical analyses were conducted using a two-sample t test with equal variance in Prism software. Values represent mean values ± SD.

Neurosphere Assay

Clonal primary and secondary neurosphere cultures were performed from dissociated DG dissected from 7- to 8-week-old mice as described (Walker et al., 2013). The number of neurospheres per well was counted 10 days after plating.

ChIP-seq Data Generation and Processing

For chromatin immunoprecipitation, adult hippocampus-derived NSCs were fixed and processed as described (Castro et al., 2011) and immunoprecipitated using a rabbit anti-Ascl1 antibody (Abcam, ab74065, 4.5 μg per ChIP sample). DNA libraries were prepared and sequences analyzed as described (Martynoga et al., 2013). A total of 13.5 million nonredundant reads were used to call peaks, and only peaks with an FDR-corrected q value $\leq 1 \times 10^{-5}$ were used for the analysis. p300 and H3K27ac data and active enhancer definitions in NSCs were from Martynoga et al. [2013]).

ACCESSION NUMBERS

The European Nucleotide Archive accession number for the Ascl1 ChIP-Seq data reported in this paper is PRJEB5023.

SUPPLEMENTAL INFORMATION

Supplemental Information includes seven figures, two tables, and Supplemental Experimental Procedures and can be found with this article online at http://dx.doi.org/10.1016/j.neuron.2014.08.004.

ACKNOWLEDGMENTS

We gratefully acknowledge Lan Chen for helping with AH-NSC cultures, Marie Caulfield and Rekha Subramaniams for helping manage the mouse colony, Graham Preece and Wayne Turnbull for helping with flow cytometry, Magdalena Götz for providing *Glast-CreERT2* mice, Sebastian Jessberger for providing AH-NSCs, James Brock and Hayley Wood for helping with figure designs, Stephen Martin for advice on statistics, Yukiko Gotoh for advice on p57 immunolabeling, and Bertie Göttgens, Alex Gould, Vivian Li, Robin Lovell-Badge, and the members of the F.G. lab for discussions and comments on the manuscript. J.A. was supported by a Medical Research Council (MRC) Studentship and N.U. by an MRC Career Development Fellowship. This work was supported by a project grant from the Wellcome Trust (082347/Z/07/Z) and a Grant-in-Aid from the MRC (U117570528) to F.G. and a research grant from NIH (NIH/NINDS 1R01NS069893) to M.N.

Accepted: August 1, 2014
Published: September 3, 2014

REFERENCES

Ables, J.L., Decarolis, N.A., Johnson, M.A., Rivera, P.D., Gao, Z., Cooper, D.C., Radtke, F., Hsieh, J., and Eisch, A.J. (2010). Notch1 is required for maintenance of the reservoir of adult hippocampal stem cells. J. Neurosci. *30*, 10484–10492.

Akala, O.O., and Clarke, M.F. (2006). Hematopoietic stem cell self-renewal. Curr. Opin. Genet. Dev. *16*, 496–501.

Ansorg, A., Witte, O.W., and Urbach, A. (2012). Age-dependent kinetics of dentate gyrus neurogenesis in the absence of cyclin D2. BMC Neurosci. *13*, 46.

Berninger, B., Costa, M.R., Koch, U., Schroeder, T., Sutor, B., Grothe, B., and Götz, M. (2007). Functional properties of neurons derived from in vitro reprogrammed postnatal astroglia. J. Neurosci. *27*, 8654–8664.

Bertrand, N., Castro, D.S., and Guillemot, F. (2002). Proneural genes and the specification of neural cell types. Nat. Rev. Neurosci. *3*, 517–530.

Blank, U., Karlsson, G., and Karlsson, S. (2008). Signaling pathways governing stem-cell fate. Blood *111*, 492–503.

Bonaguidi, M.A., Wheeler, M.A., Shapiro, J.S., Stadel, R.P., Sun, G.J., Ming, G.L., and Song, H. (2011). In vivo clonal analysis reveals self-renewing and multipotent adult neural stem cell characteristics. Cell *145*, 1142–1155.

Bonaguidi, M.A., Song, J., Ming, G.L., and Song, H. (2012). A unifying hypothesis on mammalian neural stem cell properties in the adult hippocampus. Curr. Opin. Neurobiol. *22*, 754–761.

Bracko, O., Singer, T., Aigner, S., Knobloch, M., Winner, B., Ray, J., Clemenson, G.D., Jr., Suh, H., Couillard-Despres, S., Aigner, L., et al. (2012). Gene expression profiling of neural stem cells and their neuronal progeny reveals IGF2 as a regulator of adult hippocampal neurogenesis. J. Neurosci. *32*, 3376–3387.

Breunig, J.J., Silbereis, J., Vaccarino, F.M., Sestan, N., and Rakic, P. (2007). Notch regulates cell fate and dendrite morphology of newborn neurons in the postnatal dentate gyrus. Proc. Natl. Acad. Sci. USA *104*, 20558–20563.

Calvo, C.F., Fontaine, R.H., Soueid, J., Tammela, T., Makinen, T., Alfaro-Cervello, C., Bonnaud, F., Miguez, A., Benhaim, L., Xu, Y., et al. (2011). Vascular endothelial growth factor receptor 3 directly regulates murine neurogenesis. Genes Dev. *25*, 831–844.

Castro, D.S., Martynoga, B., Parras, C., Ramesh, V., Pacary, E., Johnston, C., Drechsel, D., Lebel-Potter, M., Garcia, L.G., Hunt, C., et al. (2011). A novel function of the proneural factor Ascl1 in progenitor proliferation identified by genome-wide characterization of its targets. Genes Dev. *25*, 930–945.

Codega, P., Silva-Vargas, V., Paul, A., Maldonado-Soto, A.R., Deleo, A.M., Pastrana, E., and Doetsch, F. (2014). Prospective identification and purification of quiescent adult neural stem cells from their in vivo niche. Neuron *82*, 545–559.

Ehm, O., Göritz, C., Covic, M., Schäffner, I., Schwarz, T.J., Karaca, E., Kempkes, B., Kremmer, E., Pfrieger, F.W., Espinosa, L., et al. (2010). RBPJkappa-dependent signaling is essential for long-term maintenance of neural stem cells in the adult hippocampus. J. Neurosci. *30*, 13794–13807.

Encinas, J.M., Michurina, T.V., Peunova, N., Park, J.H., Tordo, J., Peterson, D.A., Fishell, G., Koulakov, A., and Enikolopov, G. (2011). Division-coupled astrocytic differentiation and age-related depletion of neural stem cells in the adult hippocampus. Cell Stem Cell *8*, 566–579.

Fuchs, E., Tumbar, T., and Guasch, G. (2004). Socializing with the neighbors: stem cells and their niche. Cell *116*, 769–778.

Fuentealba, L.C., Obernier, K., and Alvarez-Buylla, A. (2012). Adult neural stem cells bridge their niche. Cell Stem Cell *10*, 698–708.

Furutachi, S., Matsumoto, A., Nakayama, K.I., and Gotoh, Y. (2013). p57 controls adult neural stem cell quiescence and modulates the pace of lifelong neurogenesis. EMBO J. *32*, 970–981.

Galichet, C., Guillemot, F., and Parras, C.M. (2008). Neurogenin 2 has an essential role in development of the dentate gyrus. Development *135*, 2031–2041.

Goldstein, J., and Horsley, V. (2012). Home sweet home: skin stem cell niches. Cell. Mol. Life Sci. *69*, 2573–2582.

Han, H., Tanigaki, K., Yamamoto, N., Kuroda, K., Yoshimoto, M., Nakahata, T., Ikuta, K., and Honjo, T. (2002). Inducible gene knockout of transcription factor recombination signal binding protein-J reveals its essential role in T versus B lineage decision. Int. Immunol. *14*, 637–645.

Imayoshi, I., Isomura, A., Harima, Y., Kawaguchi, K., Kori, H., Miyachi, H., Fujiwara, T., Ishidate, F., and Kageyama, R. (2013). Oscillatory control of factors determining multipotency and fate in mouse neural progenitors. Science *342*, 1203–1208.

Jang, M.H., Bonaguidi, M.A., Kitabatake, Y., Sun, J., Song, J., Kang, E., Jun, H., Zhong, C., Su, Y., Guo, J.U., et al. (2013). Secreted frizzled-related protein 3 regulates activity-dependent adult hippocampal neurogenesis. Cell Stem Cell *12*, 215–223.

Kageyama, R., Ohtsuka, T., Hatakeyama, J., and Ohsawa, R. (2005). Roles of bHLH genes in neural stem cell differentiation. Exp. Cell Res. *306*, 343–348.

Kempermann, G., Jessberger, S., Steiner, B., and Kronenberg, G. (2004). Milestones of neuronal development in the adult hippocampus. Trends Neurosci. *27*, 447–452.

Kim, E.J., Ables, J.L., Dickel, L.K., Eisch, A.J., and Johnson, J.E. (2011). Ascl1 (Mash1) defines cells with long-term neurogenic potential in subgranular and subventricular zones in adult mouse brain. PLoS ONE *6*, e18472.

Klempin, F., Beis, D., Mosienko, V., Kempermann, G., Bader, M., and Alenina, N. (2013). Serotonin is required for exercise-induced adult hippocampal neurogenesis. J. Neurosci. *33*, 8270–8275.

Knobloch, M., Braun, S.M., Zurkirchen, L., von Schoultz, C., Zamboni, N., Araúzo-Bravo, M.J., Kovacs, W.J., Karalay, O., Suter, U., Machado, R.A., et al. (2013). Metabolic control of adult neural stem cell activity by Fasn-dependent lipogenesis. Nature *493*, 226–230.

Kronenberg, G., Reuter, K., Steiner, B., Brandt, M.D., Jessberger, S., Yamaguchi, M., and Kempermann, G. (2003). Subpopulations of proliferating cells of the adult hippocampus respond differently to physiologic neurogenic stimuli. J. Comp. Neurol. *467*, 455–463.

Ku, C.J., Hosoya, T., Maillard, I., and Engel, J.D. (2012). GATA-3 regulates hematopoietic stem cell maintenance and cell-cycle entry. Blood *119*, 2242–2251.

Kuang, S., Gillespie, M.A., and Rudnicki, M.A. (2008). Niche regulation of muscle satellite cell self-renewal and differentiation. Cell Stem Cell *2*, 22–31.

Lavado, A., Lagutin, O.V., Chow, L.M., Baker, S.J., and Oliver, G. (2010). Prox1 is required for granule cell maturation and intermediate progenitor maintenance during brain neurogenesis. PLoS Biol. *8*, e1000460.

Lee, S.W., Clemenson, G.D., and Gage, F.H. (2011). New neurons in an aged brain. Behav. Brain Res. *227*, 497–507.

Lie, D.C., Colamarino, S.A., Song, H.J., Désiré, L., Mira, H., Consiglio, A., Lein, E.S., Jessberger, S., Lansford, H., Dearie, A.R., and Gage, F.H. (2005). Wnt signalling regulates adult hippocampal neurogenesis. Nature *437*, 1370–1375.

Lien, W.H., Guo, X., Polak, L., Lawton, L.N., Young, R.A., Zheng, D., and Fuchs, E. (2011). Genome-wide maps of histone modifications unwind in vivo chromatin states of the hair follicle lineage. Cell Stem Cell *9*, 219–232.

Lieu, Y.K., and Reddy, E.P. (2009). Conditional c-myb knockout in adult hematopoietic stem cells leads to loss of self-renewal due to impaired proliferation and accelerated differentiation. Proc. Natl. Acad. Sci. USA *106*, 21689–21694.

Lugert, S., Basak, O., Knuckles, P., Haussler, U., Fabel, K., Götz, M., Haas, C.A., Kempermann, G., Taylor, V., and Giachino, C. (2010). Quiescent and active hippocampal neural stem cells with distinct morphologies respond selectively to physiological and pathological stimuli and aging. Cell Stem Cell *6*, 445–456.

Lugert, S., Vogt, M., Tchorz, J.S., Müller, M., Giachino, C., and Taylor, V. (2012). Homeostatic neurogenesis in the adult hippocampus does not involve amplification of Ascl1(high) intermediate progenitors. Nat. Commun. *3*, 670.

Machida, Y.J., Hamlin, J.L., and Dutta, A. (2005). Right place, right time, and only once: replication initiation in metazoans. Cell *123*, 13–24.

Martynoga, B., Mateo, J.L., Zhou, B., Andersen, J., Achimastou, A., Urbán, N., van den Berg, D., Georgopoulou, D., Hadjur, S., Wittbrodt, J., et al. (2013). Epigenomic enhancer annotation reveals a key role for NFIX in neural stem cell quiescence. Genes Dev. *27*, 1769–1786.

Ming, G.L., and Song, H. (2011). Adult neurogenesis in the mammalian brain: significant answers and significant questions. Neuron *70*, 687–702.

Mira, H., Andreu, Z., Suh, H., Lie, D.C., Jessberger, S., Consiglio, A., San Emeterio, J., Hortigüela, R., Marqués-Torrejón, M.A., Nakashima, K., et al. (2010). Signaling through BMPR-IA regulates quiescence and long-term activity of neural stem cells in the adult hippocampus. Cell Stem Cell *7*, 78–89.

Molofsky, A.V., Slutsky, S.G., Joseph, N.M., He, S., Pardal, R., Krishnamurthy, J., Sharpless, N.E., and Morrison, S.J. (2006). Increasing p16INK4a expression decreases forebrain progenitors and neurogenesis during ageing. Nature *443*, 448–452.

Mori, T., Tanaka, K., Buffo, A., Wurst, W., Kühn, R., and Götz, M. (2006). Inducible gene deletion in astroglia and radial glia—a valuable tool for functional and lineage analysis. Glia *54*, 21–34.

Nagy, A., Moens, C., Ivanyi, E., Pawling, J., Gertsenstein, M., Hadjantonakis, A.K., Pirity, M., and Rossant, J. (1998). Dissecting the role of N-myc in

development using a single targeting vector to generate a series of alleles. Curr. Biol. *8*, 661–664.

Nakashima, K., Takizawa, T., Ochiai, W., Yanagisawa, M., Hisatsune, T., Nakafuku, M., Miyazono, K., Kishimoto, T., Kageyama, R., and Taga, T. (2001). BMP2-mediated alteration in the developmental pathway of fetal mouse brain cells from neurogenesis to astrocytogenesis. Proc. Natl. Acad. Sci. USA *98*, 5868–5873.

Namihira, M., Kohyama, J., Semi, K., Sanosaka, T., Deneen, B., Taga, T., and Nakashima, K. (2009). Committed neuronal precursors confer astrocytic potential on residual neural precursor cells. Dev. Cell *16*, 245–255.

Niu, W., Zou, Y., Shen, C., and Zhang, C.L. (2011). Activation of postnatal neural stem cells requires nuclear receptor TLX. J. Neurosci. *31*, 13816–13828.

Oishi, K., Watatani, K., Itoh, Y., Okano, H., Guillemot, F., Nakajima, K., and Gotoh, Y. (2009). Selective induction of neocortical GABAergic neurons by the PDK1-Akt pathway through activation of Mash1. Proc. Natl. Acad. Sci. USA *106*, 13064–13069.

Orford, K.W., and Scadden, D.T. (2008). Deconstructing stem cell self-renewal: genetic insights into cell-cycle regulation. Nat. Rev. Genet. *9*, 115–128.

Osada, H., Tomida, S., Yatabe, Y., Tatematsu, Y., Takeuchi, T., Murakami, H., Kondo, Y., Sekido, Y., and Takahashi, T. (2008). Roles of achaete-scute homologue 1 in DKK1 and E-cadherin repression and neuroendocrine differentiation in lung cancer. Cancer Res. *68*, 1647–1655.

Osorio, K.M., Lee, S.E., McDermitt, D.J., Waghmare, S.K., Zhang, Y.V., Woo, H.N., and Tumbar, T. (2008). Runx1 modulates developmental, but not injury-driven, hair follicle stem cell activation. Development *135*, 1059–1068.

Pacary, E., Heng, J., Azzarelli, R., Riou, P., Castro, D., Lebel-Potter, M., Parras, C., Bell, D.M., Ridley, A.J., Parsons, M., and Guillemot, F. (2011). Proneural transcription factors regulate different steps of cortical neuron migration through Rnd-mediated inhibition of RhoA signaling. Neuron *69*, 1069–1084.

Parras, C.M., Galli, R., Britz, O., Soares, S., Galichet, C., Battiste, J., Johnson, J.E., Nakafuku, M., Vescovi, A., and Guillemot, F. (2004). Mash1 specifies neurons and oligodendrocytes in the postnatal brain. EMBO J. *23*, 4495–4505.

Pastrana, E., Cheng, L.C., and Doetsch, F. (2009). Simultaneous prospective purification of adult subventricular zone neural stem cells and their progeny. Proc. Natl. Acad. Sci. USA *106*, 6387–6392.

Ponti, G., Obernier, K., Guinto, C., Jose, L., Bonfanti, L., and Alvarez-Buylla, A. (2013). Cell cycle and lineage progression of neural progenitors in the ventricular-subventricular zones of adult mice. Proc. Natl. Acad. Sci. USA *110*, E1045–E1054.

Qu, Q., Sun, G., Li, W., Yang, S., Ye, P., Zhao, C., Yu, R.T., Gage, F.H., Evans, R.M., and Shi, Y. (2010). Orphan nuclear receptor TLX activates Wnt/beta-catenin signalling to stimulate neural stem cell proliferation and self-renewal. Nat. Cell Biol. *12*, 31–40.

Qu, Q., Sun, G., Murai, K., Ye, P., Li, W., Asuelime, G., Cheung, Y.T., and Shi, Y. (2013). Wnt7a regulates multiple steps of neurogenesis. Mol. Cell. Biol. *33*, 2551–2559.

Ramachandran, R., Zhao, X.F., and Goldman, D. (2011). Ascl1a/Dkk/beta-catenin signaling pathway is necessary and glycogen synthase kinase-3beta inhibition is sufficient for zebrafish retina regeneration. Proc. Natl. Acad. Sci. USA *108*, 15858–15863.

Renault, V.M., Rafalski, V.A., Morgan, A.A., Salih, D.A., Brett, J.O., Webb, A.E., Villeda, S.A., Thekkat, P.U., Guillerey, C., Denko, N.C., et al. (2009). FoxO3 regulates neural stem cell homeostasis. Cell Stem Cell *5*, 527–539.

Rheinbay, E., Suva, M.L., Gillespie, S.M., Wakimoto, H., Patel, A.P., Shahid, M., Oksuz, O., Rabkin, S.D., Martuza, R.L., Rivera, M.N., et al. (2013). An aberrant transcription factor network essential for Wnt signaling and stem cell maintenance in glioblastoma. Cell Rep. *3*, 1567–1579.

Seib, D.R., Corsini, N.S., Ellwanger, K., Plaas, C., Mateos, A., Pitzer, C., Niehrs, C., Celikel, T., and Martin-Villalba, A. (2013). Loss of Dickkopf-1 restores neurogenesis in old age and counteracts cognitive decline. Cell Stem Cell *12*, 204–214.

Simons, B.D., and Clevers, H. (2011). Strategies for homeostatic stem cell self-renewal in adult tissues. Cell *145*, 851–862.

Slezak, M., Göritz, C., Niemiec, A., Frisén, J., Chambon, P., Metzger, D., and Pfrieger, F.W. (2007). Transgenic mice for conditional gene manipulation in astroglial cells. Glia *55*, 1565–1576.

Souroullas, G.P., Salmon, J.M., Sablitzky, F., Curtis, D.J., and Goodell, M.A. (2009). Adult hematopoietic stem and progenitor cells require either Lyl1 or Scl for survival. Cell Stem Cell *4*, 180–186.

Srinivas, S., Watanabe, T., Lin, C.S., William, C.M., Tanabe, Y., Jessell, T.M., and Costantini, F. (2001). Cre reporter strains produced by targeted insertion of EYFP and ECFP into the ROSA26 locus. BMC Dev. Biol. *1*, 4.

Stoeber, K., Tlsty, T.D., Happerfield, L., Thomas, G.A., Romanov, S., Bobrow, L., Williams, E.D., and Williams, G.H. (2001). DNA replication licensing and human cell proliferation. J. Cell Sci. *114*, 2027–2041.

Suh, H., Consiglio, A., Ray, J., Sawai, T., D'Amour, K.A., and Gage, F.H. (2007). In vivo fate analysis reveals the multipotent and self-renewal capacities of Sox2+ neural stem cells in the adult hippocampus. Cell Stem Cell *1*, 515–528.

Venezia, T.A., Merchant, A.A., Ramos, C.A., Whitehouse, N.L., Young, A.S., Shaw, C.A., and Goodell, M.A. (2004). Molecular signatures of proliferation and quiescence in hematopoietic stem cells. PLoS Biol. *2*, e301.

Vooijs, M., Jonkers, J., and Berns, A. (2001). A highly efficient ligand-regulated Cre recombinase mouse line shows that LoxP recombination is position dependent. EMBO Rep. *2*, 292–297.

Walker, T.L., Wierick, A., Sykes, A.M., Waldau, B., Corbeil, D., Carmeliet, P., and Kempermann, G. (2013). Prominin-1 allows prospective isolation of neural stem cells from the adult murine hippocampus. J. Neurosci. *33*, 3010–3024.

Webb, A.E., Pollina, E.A., Vierbuchen, T., Urbán, N., Ucar, D., Leeman, D.S., Martynoga, B., Sewak, M., Rando, T.A., Guillemot, F., et al. (2013). FOXO3 shares common targets with ASCL1 genome-wide and inhibits ASCL1-dependent neurogenesis. Cell Rep. *4*, 477–491.

Yang, N., Ng, Y.H., Pang, Z.P., Südhof, T.C., and Wernig, M. (2011). Induced neuronal cells: how to make and define a neuron. Cell Stem Cell *9*, 517–525.

Zhang, C.L., Zou, Y., He, W., Gage, F.H., and Evans, R.M. (2008). A role for adult TLX-positive neural stem cells in learning and behaviour. Nature *451*, 1004–1007.

Zhang, K., Sha, J., and Harter, M.L. (2010). Activation of Cdc6 by MyoD is associated with the expansion of quiescent myogenic satellite cells. J. Cell Biol. *188*, 39–48.

Neuron, Volume *83*

Supplemental Information

A Transcriptional Mechanism Integrating Inputs from Extracellular Signals to Activate Hippocampal Stem Cells

Jimena Andersen, Noelia Urbán, Angeliki Achimastou, Ayako Ito, Milesa Simic, Kristy Ullom, Ben Martynoga, Mélanie Lebel, Christian Göritz, Jonas Frisén, Masato Nakafuku, and François Guillemot

Figure S1

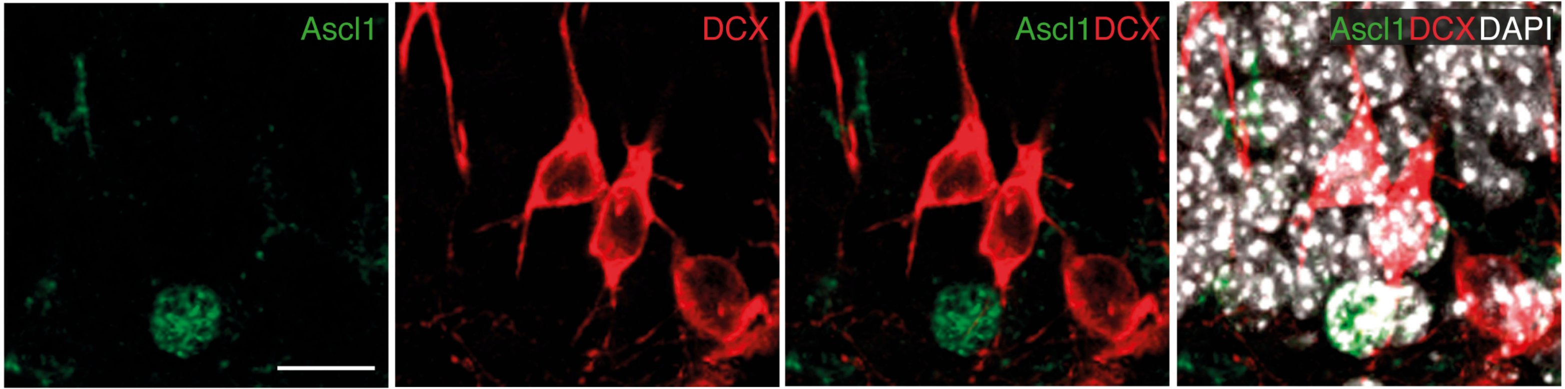

Figure S2

A

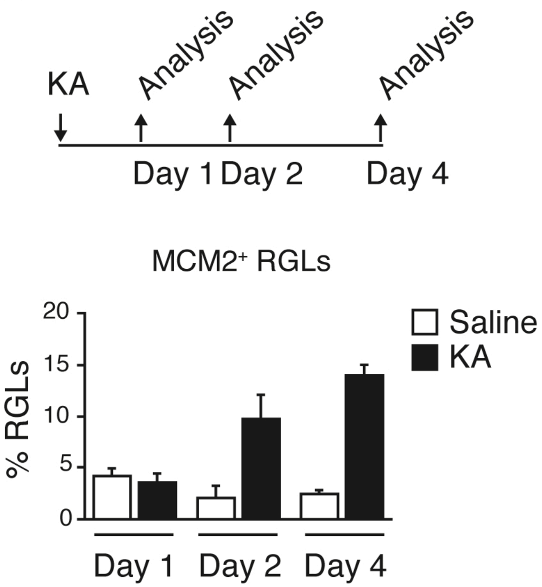

B

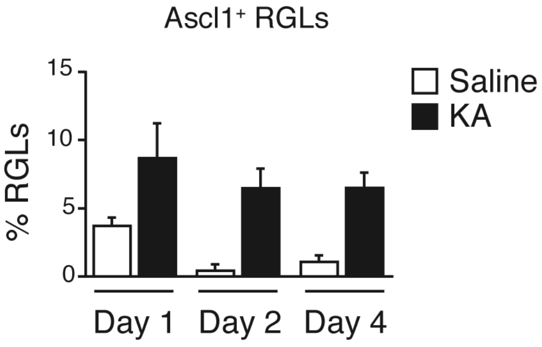

C

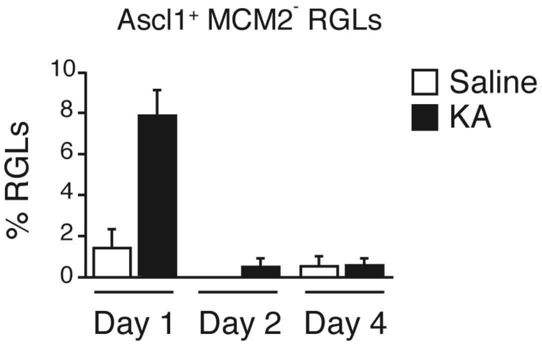

Figure S3 A-H

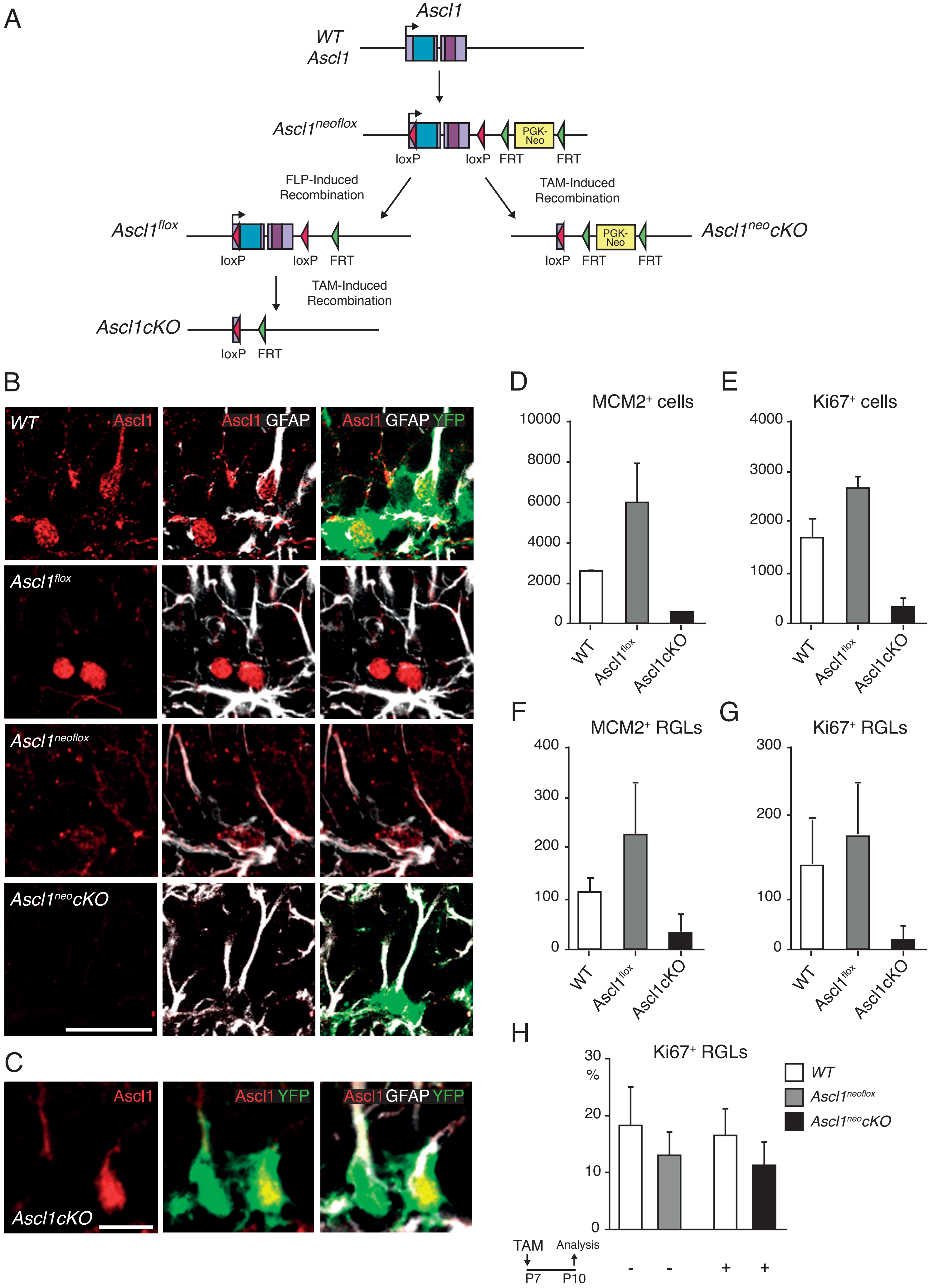

A
Ascl1
WT Ascl1
Ascl1neoflox
PGK-Neo
loxP
loxP
FRT
FRT
FLP-Induced Recombination
TAM-Induced Recombination
Ascl1flox
Ascl1neocKO
TAM-Induced Recombination
Ascl1cKO
B
WT
Ascl1
Ascl1 GFAP
Ascl1 GFAP YFP
Ascl1flox
Ascl1neoflox
Ascl1neocKO
C
Ascl1
Ascl1 YFP
Ascl1 GFAP YFP
Ascl1cKO
D
MCM2+ cells
E
Ki67+ cells
F
MCM2+ RGLs
G
Ki67+ RGLs
WT
Ascl1flox
Ascl1cKO
H
Ki67+ RGLs
%
WT
Ascl1neoflox
Ascl1neocKO
TAM
Analysis
P7
P10

Figure S3 I-K

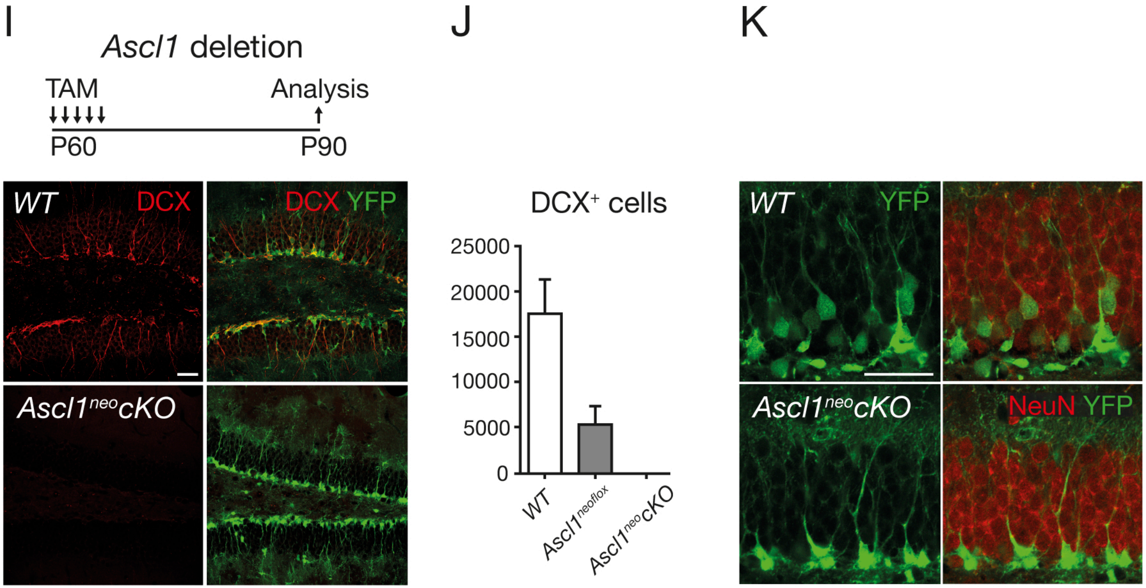

I
Ascl1 deletion
TAM
Analysis
P60
P90
WT
DCX
DCX YFP
Ascl1neocKO
J
DCX+ cells
25000
20000
15000
10000
5000
0
WT
Ascl1neoflox
Ascl1neocKO
K
WT
YFP
Ascl1neocKO
NeuN YFP

Figure S4

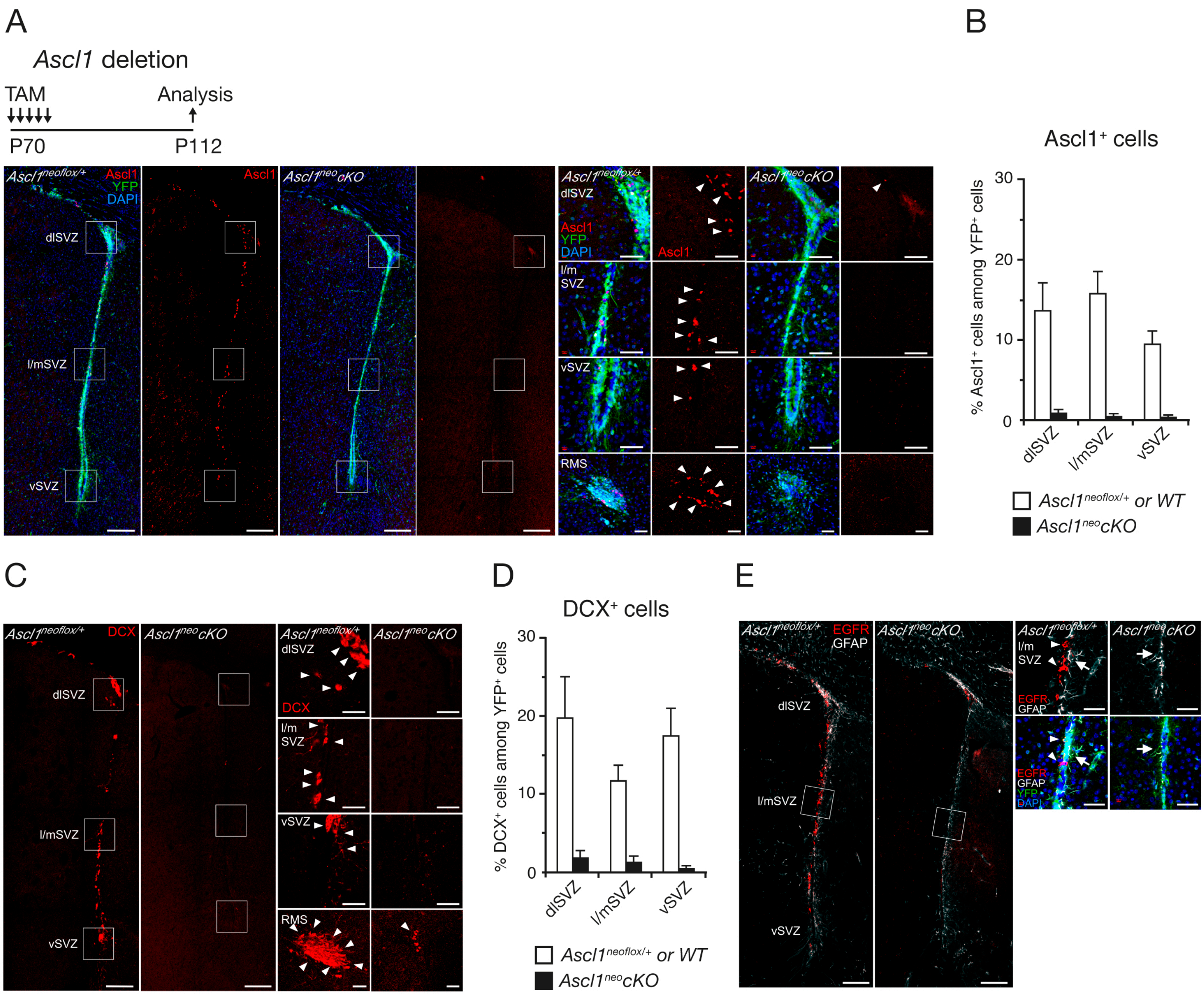

A
Ascl1 deletion
TAM
Analysis
P70
P112
Ascl1neoflox/+
Ascl1
YFP
DAPI
Ascl1neocKO
dlSVZ
l/mSVZ
vSVZ
l/m
SVZ
RMS
B
Ascl1+ cells
% Ascl1+ cells among YFP+ cells
30
20
10
0
Ascl1neoflox/+ or WT
Ascl1neocKO
C
DCX
D
DCX+ cells
% DCX+ cells among YFP+ cells
E
EGFR
GFAP

Figure S5

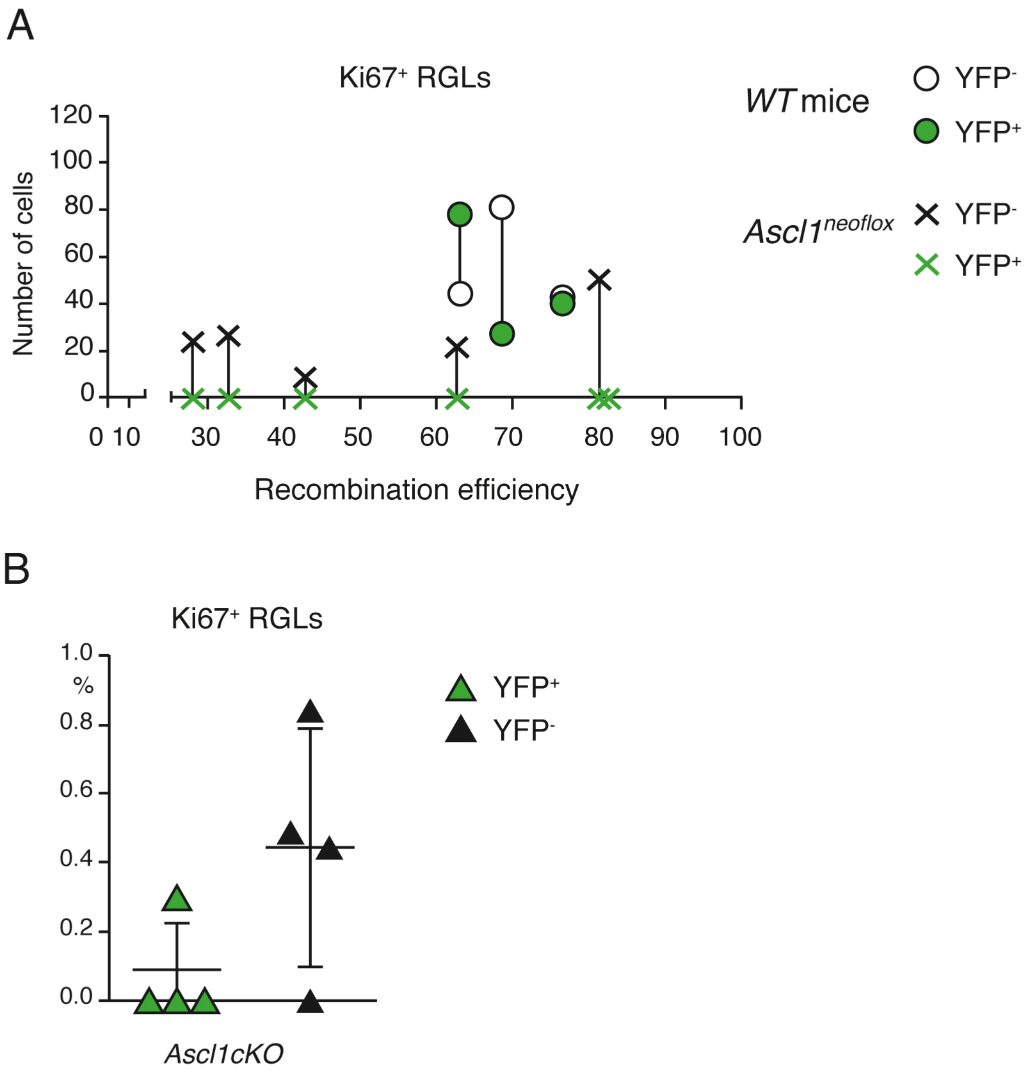
A
Ki67+ RGLs
WT mice
YFP-
YFP+
Ascl1neoflox
YFP-
YFP+
Number of cells
120
100
80
60
40
20
0
0 10 30 40 50 60 70 80 90 100
Recombination efficiency
B
Ki67+ RGLs
1.0
%
0.8
0.6
0.4
0.2
0.0
YFP+
YFP-
Ascl1cKO

Figure S6

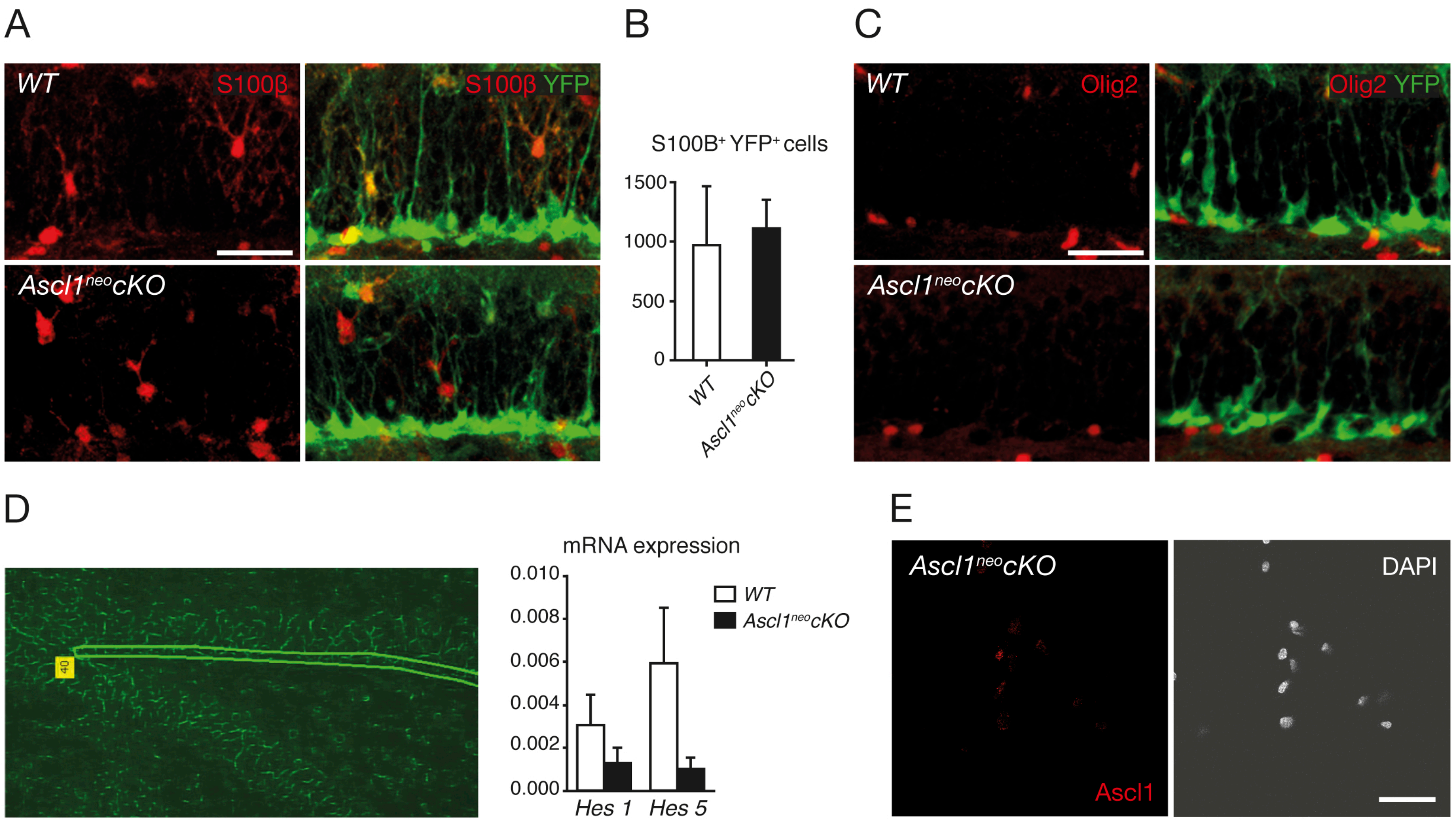
A
WT
S100β
S100β YFP
Ascl1neocKO
B
S100B+ YFP+ cells
1500
1000
500
0
WT
Ascl1neocKO
C
WT
Olig2
Olig2 YFP
Ascl1neocKO
D
40
mRNA expression
0.010
0.008
0.006
0.004
0.002
0.000
WT
Ascl1neocKO
Hes 1
Hes 5
E
Ascl1neocKO
DAPI
Ascl1

Figure S7

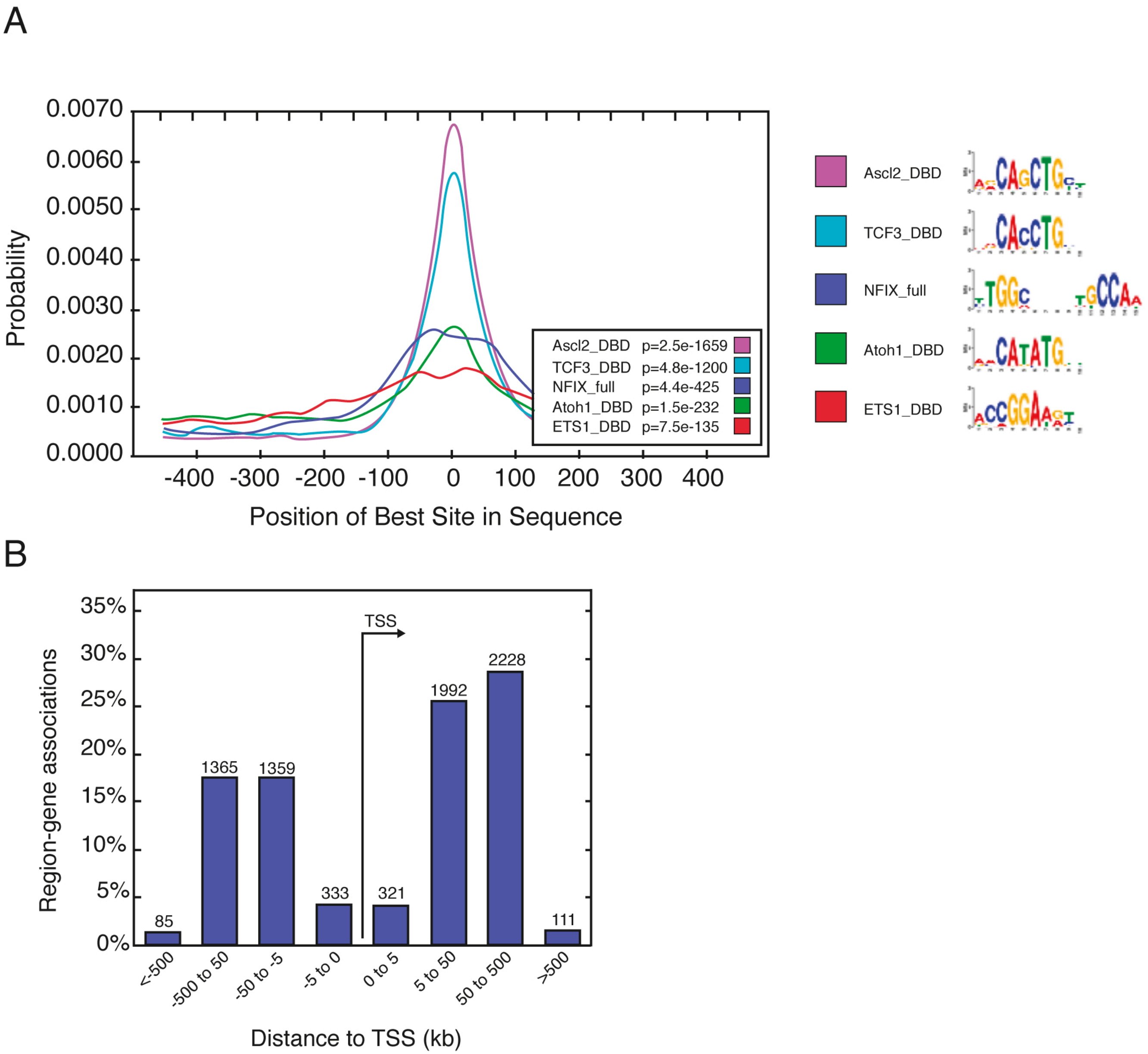
A
0.0070
0.0060
0.0050
0.0040
0.0030
0.0020
0.0010
0.0000
Probability
-400 -300 -200 -100 0 100 200 300 400
Position of Best Site in Sequence
Ascl2_DBD p=2.5e-1659
TCF3_DBD p=4.8e-1200
NFIX_full p=4.4e-425
Atoh1_DBD p=1.5e-232
ETS1_DBD p=7.5e-135
Ascl2_DBD
TCF3_DBD
NFIX_full
Atoh1_DBD
ETS1_DBD
B
35%
30%
25%
20%
15%
10%
5%
0%
Region-gene associations
TSS
85
1365
1359
333
321
1992
2228
111
<-500
-500 to 50
-50 to -5
-5 to 0
0 to 5
5 to 50
50 to 500
>500
Distance to TSS (kb)

SUPPLEMENTAL INFORMATION

Supplemental Information includes six Supplemental Figures, Supplemental Experimental Procedures and Supplemental References.

Supplemental figure legends

Figure S1. Ascl1 is not expressed in DCX^+ neuroblasts

Labelling for Ascl1 and the neuroblast marker DCX in the DG of a P60 *WT* mouse shows that Ascl1 is not expressed by DG neuroblasts. Scale bar represents 20μm.

Figure S2. Induction of Ascl1 expression and stem cell activity in the DG of KA-injected mice

(A and B) The percentages of RGLs that express Ascl1 (A) or express MCM2 (B) 24 hours, 2 days and 4 days after saline and KA injections show that KA induces Ascl1 expression more rapidly than it promotes RGL activation. p-values: $MCM2^+$ RGLs in saline vs KA at 1 day = 0.6492, 2 days = 0.0578, 4 days = 0.0005; $Ascl1^+$ RGLs in saline vs KA at 1 day = 0.1567, 2 days = 0.0224, and 4 days = 0.0149. n = 3 (saline day 2 and day 4), 4 (saline day 1, KA day 2 and 4), 5 (KA day 1).

(C) The percentages of Ascl1-expressing quiescent ($MCM2^-$) RGLs 24 hours, 2 days and 4 days after saline and KA injections show that KA induces Ascl1 expression in RGLs that are still quiescent. p-values: $Ascl1^+MCM2^-$ RGLs in saline vs KA at 1 day = 0.0074, 2 days = 0.4366, 4 days = 0.9385. Values represent mean values, and error bars represent standard deviations.

Figure S3. Expression of Ascl1 and proliferation of SGZ cells and RGLs in *Ascl1^{flox}* and *Ascl1cKO* mice

(A) Scheme showing how the different mutant alleles of *Ascl1* used in this study were generated.

(B) Labelling for Ascl1, GFAP and YFP shows that Ascl1 expression is markedly reduced in the non-recombined RGLs of *Ascl1^{neoflox}* DG and is absent in recombined RGLs of *Ascl1^{neo}cKO* DG.

(C) Labelling for Ascl1, YFP and GFAP in *Ascl1cKO* DG shows that some RGLs express YFP and have therefore recombined the *Rosa26 YFP* reporter allele but also express Ascl1 and have therefore not recombined the *Ascl1* gene.

(D to G) Total numbers per DG of SGZ cells (D, E) and RGLs (F, G) expressing MCM2 (D, F) and Ki67 (E, G) in WT, *Ascl1^{flox}*, and *Ascl1cKO* DG. Note the small number of proliferating SGZ cells and activated RGLs remaining in the *Ascl1cKO* DG due to the lack of complete recombination of the *Ascl1^{flox}* allele. Note also the different numbers of proliferating RGLs found in *WT* and *Ascl1^{flox}* mice, which might be due to expression of Cre in *WT* but not *Ascl1^{flox}* RGLs (see Figure 3C). p-values: *WT* vs *Ascl1cKO*: Mcm2^{+} cells = 0.0001, Ki67^{+} cells = 0.0003; Mcm2^{+} RGLs = 0.0254, Ki67^{+} RGLs = 0.0108; *Ascl1^{flox}* vs *Ascl1cKO*, MCM2^{+} cells = 0.0005, Ki67^{+} cells = 0.0001, MCM2^{+} RGLs p = 0.0075, Ki67^{+} RGLs = 0.0056. n = 3 in *WT* and *Ascl1^{flox}* and n = 5 in *Ascl1cKO*.

(H) Percentages of proliferating RGLs in the DG of P10 *WT* and *Ascl1^{neoflox}* mice, untreated with tamoxifen (left bars) and in the DG of P10 *WT* and *Ascl1^{neo}cKO* mice

injected with tamoxifen at P7 (right bars). In contrast with the requirement for *Ascl1* in adult RGL proliferation, the hypomorphic allele of *Ascl1* ($Ascl1^{neoflox}$) and the deletion of *Ascl1* ($Ascl1^{neo}cKO$) do not significantly reduce RGL proliferation at this postnatal stage. p-values: *WT* vs $Ascl1^{neoflox}$ = 0.2159. N = 5 for *WT* and n = 4 for $Ascl1^{neoflox}$. *WT* vs $Ascl1^{neo}cKO$ = 0.2309. n = 3 for each.

(I and J) DCX^+ neuroblasts are missing in the DG of $Ascl1^{neo}cKO$ mice. Total numbers of DCX^+ cells per DG. p-value: DCX^+ cells in *WT vs* $Ascl1^{neo}cKO$ = 0.0010. n = 3 in each genotype.

(K) The recently born YFP^+ $NeuN^+$ granule cells seen in the *WT* DG (green cells above the SGZ) are absent in the $Ascl1^{neo}cKO$ DG. Scale bars represent 20μm in B, 10μm in C, and 40μm in I and K. Values represent mean values, and error bars represent standard deviations.

Figure S4. Block of activation and proliferation of V-SVZ stem cells by conditional inactivation of *Ascl1*

(A,B) Labelling for Ascl1 (A) and percentages of $Ascl1^+$ cells among recombined YFP^+ cells in the dorsal (dlSVZ), medial and lateral (l/mSVZ) and ventral (vSVZ) V-SVZ show the efficiency of *Ascl1* deletion in the V-SVZ of $Ascl1^{neo}cKO$ mice. p-values in *WT vs* $Ascl1^{neo}cKO$ $Ascl1^+$ cells = 0.0015 in dlSVZ, = 0.0003 in l/mSVZ, = 0.0003 in vSVZ; n = 5.

(C,D) Labelling for the neuroblast marker DCX (C) and percentages of DCX^+ cells among YFP^+ cells in the dlSVZ, l/mSVZ and vSVZ (D) show that RGLs are not activated and do not generate neuroblasts in the V-SVZ of P112 $Ascl1^{neo}cKO$ mice. p-

values in *WT vs Ascl1^{neo}cKO* DCX$^+$ cells = 0.0022 in dlSVZ, = 0.0004 in l/mSVZ, = 0.0004 in vSVZ; n = 5.

(E) Labelling for the RGL marker GFAP, the activation marker EGFR and YFP to identify recombined cells (right panels are enlargements of the areas boxed in left panels; arrowheads point to GFAP$^+$, EGFR$^+$ activated RGLs and arrows point to GFAP$^+$ EGFR$^-$ inactive RGLs). Scale bars represent 200μm in whole panels and 50μm in enlarged panels. Values represent mean values, and error bars represent standard deviations.

Figure S5. RGLs that have recombined in mosaic *Ascl1^{neo}cKO* and *Ascl1cKO* mice do not proliferate

(A) Mosaic analysis in *Ascl1^{neo}cKO* mice**.** For each *WT* and *Ascl1^{neo}cKO* mouse studied, the numbers of Ki67$^+$ RGLs (vertical axis) have been plotted separately for recombined (YFP$^+$) and non-recombined (YFP$^-$) RGLs and are shown linked by a vertical line. The recombination efficiency (horizontal axis) is the ratio of YFP$^+$ RGLs to total RGLs in each mouse. Note that recombined RGLs fail to proliferate in all the *Ascl1^{neo}cKO* mice.

(B) Mosaic analysis in *Ascl1cKO* mice**.** For each *WT* and *Ascl1cKO* mouse studied, the numbers of Ki67$^+$ RGLs have been plotted separately for recombined (green triangles) and non-recombined (black triangles) RGLs. Recombination efficiency was between 12% and 40% of all RGLs. Note that recombined RGLs fail to proliferate in the *Ascl1cKO* mice. Expression of Ki67$^+$ in one YFP$^+$ RGL is likely due to the uncoupling between recombination of *Ascl1* and of the *Rosa26 YFP* reporter

allele (Figure S3C, see Results section). Values represent mean values, and error bars represent standard deviations.

Figure S6. *Ascl1^{neo}cKO* RGLs remain undifferentiated and do not generate neurospheres

(A-C) Labelling for YFP and the astrocytic marker S100β (A) shows that *Ascl1^{neo}cKO* RGLs do not express this marker. The total numbers of YFP^{+} S100β^{+} astrocytes in the DG (B) show that there is no increase in number in astrocytes in *Ascl1^{neo}cKO* compared with *WT* mice. Labelling for YFP and the oligodendrocyte progenitor marker Olig2 shows that RGLs do not express this marker in *Ascl1^{neo}cKO* mice (C).

(D) Quantitative RT-PCR analysis in laser capture-microdissected SGZ tissue (top panel) of transcripts for the Notch transcriptional effectors *Hes1* and *Hes5* (bottom panel) shows that Notch signalling activity is reduced in the *Ascl1^{neo}cKO* SGZ. Expression levels normalized to *Gapdh.* n = 3.

(E) Clonal neurosphere cultures of dissociated DG cells shows that the few neurospheres that are generated from *Ascl1^{neo}cKO* DG contain Ascl1^{+} cells demonstrating that they originate from DG cells that did not undergo recombination. Scale bars represent 40μm. Values represent mean values, and error bars represent standard deviations.

Figure S7. Analysis of Ascl1-binding events in the genome of AH-NSCs

(A) Frequencies of motif occurrence around the summit of Ascl1-binding peaks in AH-NSCs. A bHLH factor binding E-box motif is found most frequently near Ascl1-binding peaks.

(B) Distribution of Ascl1-binding sites relative to transcription start sites (TSS) in the AH-NSC genome. Most Ascl1-binding sites are between 5 kb and 500 kb away from a TSS.

Supplemental experimental procedures

Animals

All procedures were performed in accordance with a UK Home Office Project Licence and approved by the local ethics committee. Mice were housed in standard cages under a 12 h light/dark cycle, and had *ad libitum* access to food and water. *RBPJk*$^{loxp/loxp}$ animals were generated as previously described (Han et al., 2002). *Glast-CreERT2* mice, which allow for tamoxifen-inducible expression of Cre recombinase under the astrocyte-specific glutamate aspartate transporter (GLAST, Mori et al., 2006) promoter, were crossed with *Ascl1*$^{neoflox/neoflox}$ mice, in which exon 1 of the *Ascl1* gene is flanked by loxP sites, and with *Rosa26-floxed stop-YFP* reporter mice (R26 YFP, Srinivas et al., 2001) to generate *Glast-CreERT2/Ascl1*^{neoflox}*/R26 YFP*. In order to remove the PGK promoter-neo cassette from the *Ascl1* locus, we crossed *Ascl1*^{neoflox} animals with an *actβ-Flp* mouse line (The Jackson Laboratories, Maine). Both male and female transgenic mice were included in the analysis.

Tamoxifen and BrdU administration

For activation of the CreERT2 recombinase, postnatal day 60 (P60) animals were administered 4- Hydroxytamoxifen (TAM, Sigma-Aldrich) for 5 consecutive days (intraperitoneally, ip; 2 mg/day, stock solution 10 mg/ml dissolved in 20% EtOH/80% sunflower oil). For mosaic experiments, P60 animals received a single TAM injection at the same concentration (based on preliminary studies with a range of concentrations that showed a single injection to produce adequate mosaic recombination). For deletion of *Ascl1* during postnatal life, animals received one

injection of TAM at P7 (i.p.; 50 µl, stock solution 20 mg/ml). Analysis was performed 3 days after TAM injection. All *WT*, *Ascl1^{neoflox} a*nd *Ascl1^{neo}cKO* animals received TAM injections to avoid injection-related differences between *Ascl1^{neoflox} a*nd experimental animals. To examine progenitor proliferation, mice were given a single bromodeoxyuridine (BrdU, Sigma-Aldrich) i.p. injection (2 mg, stock solution 10 mg/ml dissolved in 0.9% saline) 2 hours prior tissue collection. In order to examine slowly-dividing progenitor proliferation, a long-term BrdU paradigm was performed. Mice received 5 daily i.p. BrdU injections (2 mg/day) followed by 5 consecutive days of BrdU-containing drinking water (1 mg/ml). Mice were sacrificed 20 days after the last day of receiving BrdU in the drinking water.

Kainic acid administration

To induce seizures, 8-9 week-old MF1 mice or P86 *Ascl1 WT* and *Ascl1^{neo}cKO* male mice received kainic acid (KA, Sigma-Aldrich) as a single i.p. injection at 19 mg/kg dissolved in 0.9% saline (sub-seizure concentration based on preliminary studies showing that progenitor proliferation is induced without animals undergoing observable convulsions). Animals were monitored for 90 minutes after KA injection, and their behaviour was scored every 10 min from 0-7 based on (Monory et al., 2006) (0- no response, 1- immobility and staring, 2- forelimb and/or tail extension, rigid posture, 3- repetitive movements, 4- rearing and falling, 5- continuous rearing and falling, 6- severe clonic-tonic seizures, 7- death). Animals reaching stage 4 were immediately killed and were not used for the experiment. Around 15% of all animals receiving KA reached stage 4. Remaining animals were then sacrificed 1, 2

or 4 days after KA injection, and processed as described below.

Tissue preparation

Animals were terminally anesthetized and transcardially perfused with 0.9% saline for 3 min followed by 4% paraformaldehyde (PFA) in PBS for 12 min. Brains were post-fixed with 4% PFA for 2 hours at 4°C and sectioned coronally at 40μm using a vibratome (Leica). These sections were kept at 4°C on 0.02% Azide until used. For $p57^{Kip2}$ staining, brains were cryo-protected in sucrose overnight (30% sucrose in PBS) before being embedded in gelatin and sucrose (7.5 and 15%, respectively, in PBS) and frozen in isopentane. Frozen brains were cut in a Microm Cryostat (Zeiss) at 30μm and kept at minus 20°C until used.

Histology

For immunofluorescence staining, free-floating or frozen sections were blocked in 10% normal donkey serum and 1% Triton X-100 in PBS for 2 hours. Sections were then incubated overnight at 4°C with primary antibodies diluted at appropriate concentrations in incubation solution (10% normal donkey serum and 0.1% Triton X-100 in PBS). The following primary antibodies were used: mouse anti Ascl1 (1/100, BD Biosciences), rat anti BrdU (1/1000, AbD Serotec), goat anti doublecortin (1/50, Santa Cruz Biotechnology), rabbit anti GFAP (1/1000, Dako), rat anti GFAP (1/1000, Invitrogen Life Technologies), rabbit anti GFP (1/1000, Invitrogen Life Technologies), rat anti GFP (1/1000, Fine Chemicals), sheep anti GFP (1/1000, AbD Serotec), rabbit anti Ki67 (1/200, Leica), goat anti MCM2 (1/50, Santa

Cruz Biotechnology), mouse anti NeuN (1/1000, Chemicon), mouse anti Nestin (1/200, Millipore), rabbit anti S100β (1/500, Dako), goat anti Sox2 (1/500, Acris Antibodies), rabbit anti Olig2 (1/500, Millipore), rabbit anti $p57^{Kip2}$ (1/200, Sigma), and rabbit anti Tbr2 (1/500, Abcam). After 3 washes with PBT (PBS + 0.1% Triton X-100), sections were incubated in incubation solution with corresponding secondary antibodies for 2 hours at a dilution of 1/1000. Secondary antibodies used were conjugated to Alexa-488, Alexa-568 (Invitrogen Life Technologies), Cy3 or Cy5 (The Jackson Laboratory). Following 3 washes with PBT, sections were incubated with 6-diamidino-2-phenylindole (DAPI, 1/10000, Sigma-Aldrich) for 20 min to obtain nuclear staining, and finally mounted in Aqua PolyMount (Polysciences). For BrdU GFP GFAP triple staining, GFP GFAP staining was performed as explained above. Sections were then fixed for 30 min with 4% PFA, washed 3 times with PBS and then pre-treated with pre-warmed 2N HCL for 30 min. BrdU primary antibody was then added after two 15 min borate buffer (0.1 M sodium tetraborate, pH 8.5 in PBS) washes to continue immunostaining as normal. For immunocytochemistry of attached neurospheres, the same protocol was used except for the initial blocking step, which was performed with 10% normal donkey serum and 0.1% Triton X-100 in PBS for 30 minutes at room temperature.

Microscopic analysis and quantification

The total number of single, double or triple antigen-positive cells was counted in every ninth 40μm section through the entire rostrocaudal length of the DG (-0.82 mm to -4.24 mm from bregma). Images were acquired using an SP5 confocal

microscope (Leica). 10 to 15 z-plane images separated by a 1μm step were obtained and counted. To present total numbers per dentate gyrus, cells counted were divided by the number of z-planes counted to obtain the number of cells per 1μm, and then multiplied by the total length of the dentate gyrus. For counts of $GFAP^+$ RGLs, cells were deemed to be radial if the cell body, clearly associated with a DAPI-positive nucleus, was located in the SGZ and had a single radial process extending through at least the length of two cell bodies from the bottom of the subgranular zone. In each experiment the DG of 3 or more mice per group were analysed. In all figures, the cell numbers counted in *WT* and *Ascl1^{neo}cKO* mice are numbers of YFP^+ $marker^+$ double-labelled cells, while the numbers counted in *Ascl1^{neoflox}* mice are for $marker^+$ cells only since YFP is not expressed in these mice.

Laser Capture microdissection

For laser capture microdissection tissue collection, brains were removed after sacrifice, rapidly frozen in OCT on dry ice and stored at -80°C until further use. 14μm coronal sections spanning the length of the dentate gyrus were cut in a cryostat (CM3050S, Leica) and placed on MembraneSlides (Zeiss). The subgranular zone of *WT*, *Ascl1^{neoflox}* and *Ascl1^{neo}cKO* was excised by a PLAM laser-capture microdissection system (Zeiss) and collected in an adhesive cap (AdhesiveCap 200, ZEISS).

Fluorescence-activated cell sorting, RNA isolation and cDNA production and quantitative real-time PCR analysis

Glast-CreERT2, *Ascl1^{neoflox}*, *R26 YFP* and *Glast-CreERT2*, *Ascl1^{WT}*, *R26 YFP* mice were treated with tamoxifen during 5 days and the DG were microdissected 4 days after the last tamoxifen injection. The protocol used was as described (Walker et al., 2013), with the exception that un-stained pellets were re-suspended in 1 ml of DMEM:F12 without phenol red. The YFP+ cells were analyzed using a FACS Aria Cell Sorter (BD Biosciences). DGs of *Glast-CreERT2* negative animals were used for YFP gating and dead cells were excluded by propidium iodide staining (1ug/ml). The DGs of three to five animals of the same genotype were pooled for each sorting and considered as one n. RNA was extracted using the RNeasy Mini Kit (Qiagen) and following manufacturer's instructions. RNA was reverse transcribed using the High Capacity cDNA Reverse Transcription Kit (Applied Biosystems) following manufacturer's instructions. Gene expression was detected using TaqMan Gene expression assays (Applied Biosystems) as described by the manufacturer and performed on a 7500 real time PCR system (Applied Biosystems). Data were analysed using standard protocols to calculate relative expression with the dCT method with *Gapdh* serving as an endogenous control. Each probe was performed in duplicates for at least 3 independent samples per group.

Neurospheres

Primary neurospheres were obtained from 7 to 8 weeks old mice as previously described (Walker et al., 2013). Briefly, animals received a daily injection of

tamoxifen for five days and were killed four days after the last injection, the brains were removed and the DG were dissected. Isolated DG of each animal were processed separately. Tissue was enzymatically dissociated using the Neural Tissue Dissociation Kit (Milteny) according to manufacturer's instructions. The resulting cell suspension was diluted in HBSS with Calcium and Magnesium (Gibco, Life Technologies) and filtered through a 40μm cell sieve (Falcon; BD Bioscience). The single cell suspension was then pelleted and resuspended at a density of 1 dentate gyrus per P96-well plate. The neurosphere media consisted of DMEM:F12 (Gibco, Life Technologies), supplemented with NeuroCult NSC Proliferation Supplements (Stem Cell Techonologies), 2% bovine serum albumin (Sigma), 15mm potassium chloride (Sigma), 2μg/ml heparin (Sigma), 10ng/ml FGF2 (Peprotech) and 20ng/ml EGF (Peprotech). Cells were incubated for 12 days in a humidified incubator (5% CO_2) at 37°C and then the number of spheres formed was counted.

For passaging of the spheres, media was almost completely removed from the wells containing single spheres and 100 μl of trypsin EDTA (Gibco, Life Sciences) was added and incubated for 2 minutes at room temperature. Then an equal volume of trypsin inhibitor (Sigma) was added and neurospheres were mechanically dissociated with a P-200 tip. Cells were re-seeded in P24-well plates with 1ml of fresh medium. The number of neurospheres per well was counted 10 days after plating. Wells containing two or more spheres were considered as having successfully self-renewed. For immunocytochemistry analysis, the neurospheres were allowed to attach to a matrigel (Growth Factor reduced, BD Biosciences) - coated coverslip during 1 hour. They were then fixed with 4% PFA in PBS for 10

minutes and stored in PBS at 4 degrees until the immunocytochemistry was performed.

Statistical analysis

Statistical analyses were conducted using a two-sample t test with equal variance in Prism software. A p-value of <0.05 was considered significant. All values represent mean values, and error bars represent standard deviations.

ChIP-seq data generation and processing

Adult hippocampus-derived neural stem cells were a kind gift from S. Jessberger and were cultured on laminin-coated flasks in DMEM with Ham's F12, supplemented with N2 supplement plus EGF, FGF and heparin. For chromatin immunoprecipitation, cells were fixed sequentially with di(N-succimidyl) glutarate and 1% formaldehyde in phosphate-buffered saline and then lysed, sonicated and immunoprecipitated as described previously (Castro et al., 2011), using a rabbit anti-Ascl1 antibody (Abcam, ab74065, 4.5μg per ChIP sample).

DNA libraries were prepared from 20ng of immunoprecipitated DNA according to the standard Illumina ChIP-seq protocol. Libraries were sequenced with the Genome Analyzer IIx (Illumina). The raw reads were mapped to the mouse genome (mm9 including random chromosomes) with Bowtie2 (Langmead et al., 2012) version 2.1.0. An input chromatin sample was mapped in the same way and used as the control sample. Significant binding peaks were called using MACS (Zhang et al., 2008) version 2.0.10, after balancing the number of reads in treatment

and control samples, as described previously (Martynoga et al., 2013). 13.5 million non-redundant reads were used to call peaks and only peaks with an FDR-corrected q-value<=$1x10^{-5}$ were used for this analysis. p300 and H3K27ac data and active enhancer definitions in NSCs were from (Martynoga et al., 2013). Ascl1 binding events in GBMSCs were obtained from (Rheinbay et al., 2013) and for comparison of binding event locations were converted to the mouse mm9 genome co-ordinates with the UCSC liftOver tool.

Supplemental references

Castro, D.S., Martynoga, B., Parras, C., Ramesh, V., Pacary, E., Johnston, C., Drechsel, D., Lebel-Potter, M., Garcia, L.G., Hunt, C.*, et al.* (2011). A novel function of the proneural factor Ascl1 in progenitor proliferation identified by genome-wide characterization of its targets. Genes Dev *25*, 930-945.

Han, H., Tanigaki, K., Yamamoto, N., Kuroda, K., Yoshimoto, M., Nakahata, T., Ikuta, K., and Honjo, T. (2002). Inducible gene knockout of transcription factor recombination signal binding protein-J reveals its essential role in T versus B lineage decision. Int Immunol *14*, 637-645.

Langmead, B., and Salzberg, S.L. (2012). Fast gapped-read alignment with Bowtie 2. Nat Methods *9*, 357-359.

Martynoga, B., Mateo, J.L., Zhou, B., Andersen, J., Achimastou, A., Urban, N., van den Berg, D., Georgopoulou, D., Hadjur, S., Wittbrodt, J.*, et al.* (2013). Epigenomic

enhancer annotation reveals a key role for NFIX in neural stem cell quiescence. Genes Dev *27*, 1769-1786.

Monory, K., Massa, F., Egertova, M., Eder, M., Blaudzun, H., Westenbroek, R., Kelsch, W., Jacob, W., Marsch, R., Ekker, M.*, et al.* (2006). The endocannabinoid system controls key epileptogenic circuits in the hippocampus. Neuron *51*, 455-466.

Mori, T., Tanaka, K., Buffo, A., Wurst, W., Kuhn, R., and Gotz, M. (2006). Inducible gene deletion in astroglia and radial glia--a valuable tool for functional and lineage analysis. Glia *54*, 21-34.

Rheinbay, E., Suva, M.L., Gillespie, S.M., Wakimoto, H., Patel, A.P., Shahid, M., Oksuz, O., Rabkin, S.D., Martuza, R.L., Rivera, M.N.*, et al.* (2013). An Aberrant Transcription Factor Network Essential for Wnt Signaling and Stem Cell Maintenance in Glioblastoma. Cell Rep *3*, 1567-1579.

Srinivas, S., Watanabe, T., Lin, C.S., William, C.M., Tanabe, Y., Jessell, T.M., and Costantini, F. (2001). Cre reporter strains produced by targeted insertion of EYFP and ECFP into the ROSA26 locus. BMC Dev Biol *1*, 4.

Walker, T.L., Wierick, A., Sykes, A.M., Waldau, B., Corbeil, D., Carmeliet, P., and Kempermann, G. (2013). Prominin-1 Allows Prospective Isolation of Neural Stem Cells from the Adult Murine Hippocampus. J Neurosci *33*, 3010-3024.

Zhang, Y., Liu, T., Meyer, C.A., Eeckhoute, J., Johnson, D.S., Bernstein, B.E., Nusbaum, C., Myers, R.M., Brown, M., Li, W.*, et al.* (2008b). Model-based analysis of ChIP-Seq (MACS). Genome Biol *9*, R137.
